# Two Differential Binding Mechanisms of FG-Nucleoporins and Nuclear Transport Receptors

**DOI:** 10.1016/j.celrep.2018.03.022

**Published:** 2018-03-27

**Authors:** Piau Siong Tan, Iker Valle Aramburu, Davide Mercadante, Swati Tyagi, Aritra Chowdhury, Daniel Spitz, Sarah L. Shammas, Frauke Gräter, Edward A. Lemke

**Affiliations:** 1 Departments of Biology and Chemistry, Pharmacy and Geosciences, Johannes Gutenberg-University Mainz, Johannes-von-Mullerweg 6, 55128 Mainz, Germany; 2 Institute of Molecular Biology (IMB), Ackermannweg 4, 55128 Mainz, Germany; 3 Structural and Computational Biology Unit & Cell Biology and Biophysics Unit, European Molecular Biology Laboratory (EMBL), Meyerhofstrasse 1, 69117 Heidelberg, Germany; 4 Heidelberg Institute for Theoretical Studies (HITS), Schloß-Wolfsbrunnenweg 35, 69118 Heidelberg, Germany; 5 Interdisciplinary Center for Scientific Computing, Heidelberg University, Mathematikon, Im Neuenheimer Feld 205, 69120 Heidelberg, Germany; 6 Department of New Biochemistry, University of Oxford, South Parks Road, Oxford OX1 3QU, UK

**Keywords:** intrinsically disordered protein, glycosylation, FG-Nup, nuclear transport receptors, binding mechanism, single-molecule FRET, molecular dynamics simulations

## Abstract

Phenylalanine-glycine-rich nucleoporins (FG-Nups) are intrinsically disordered proteins, constituting the selective barrier of the nuclear pore complex (NPC). Previous studies showed that nuclear transport receptors (NTRs) were found to interact with FG-Nups by forming an “archetypal-fuzzy” complex through the rapid formation and breakage of interactions with many individual FG motifs. Here, we use single-molecule studies combined with atomistic simulations to show that, in sharp contrast, FG-Nup214 undergoes a coupled reconfiguration-binding mechanism when interacting with the export receptor CRM1. Association and dissociation rate constants are more than an order of magnitude lower than in the archetypal-fuzzy complex between FG-Nup153 and NTRs. Unexpectedly, this behavior appears not to be encoded selectively into CRM1 but rather into the FG-Nup214 sequence. The same distinct binding mechanisms are unperturbed in O-linked β-N-acetylglucosamine-modified FG-Nups. Our results have implications for differential roles of distinctly spatially distributed FG-Nup⋅NTR interactions in the cell.

## Highlights

• Identification of two differential binding mechanisms in the nuclear transport pathway
• FG-Nup214 does not bind CRM1 via an archetypal-fuzzy complex
• Glycosylated FG-Nups maintain their NTR-binding mechanisms
• Linker regions of FG-Nups may have functional relevance to the binding mechanism

## Graphical Abstract

**Figure:**
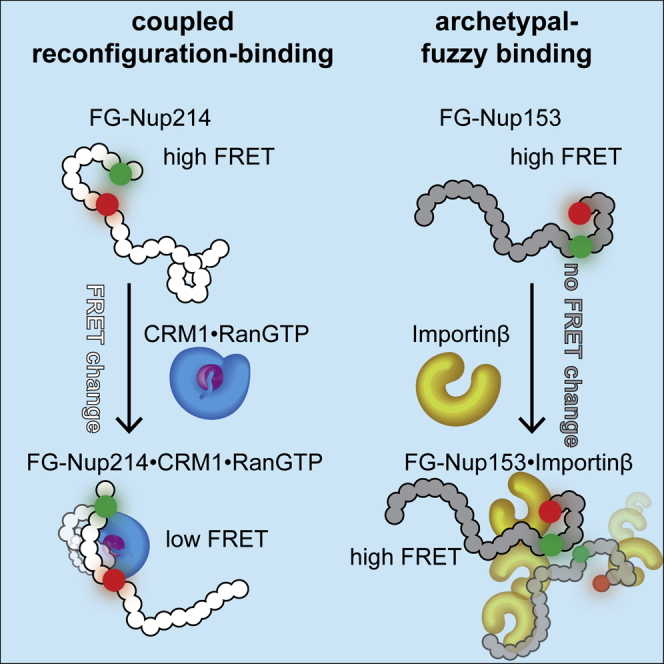

## Teaser

Archetypal-fuzzy complexes found in most FG-Nucleoporin⋅nuclear transport receptor complexes allow fast yet specific nuclear transport. Tan et al. show that FG-Nup214, located at the periphery of the nuclear pore complex, binds to CRM1⋅RanGTP via a coupled reconfiguration-binding mechanism, which can enable different functionalities e.g., cargo release.

## Introduction

Metazoan nuclear pore complexes (NPCs) are giant molecular complexes (∼120 MDa) that are located at the nuclear envelope facilitating nucleocytoplasmic traffic of cargoes. They are formed by multiple copies of around 30 distinct proteins known as nucleoporins (Nups). Approximately one-third of the Nups contain disordered regions of variable lengths rich in phenylalanine-glycine motifs (FG motifs) (Hurt, 1988, Ori et al., 2013, Wente et al., 1992). These intrinsically disordered proteins (IDPs), also known as FG-Nups, form the permeability barrier of the NPC, which acts as a selective filter, allowing the free passage of smaller cargoes (∼40 kDa) while hindering cargoes with increasing size (Timney et al., 2016). Active transport of cargoes across the NPC can only occur when they are bound to adaptor molecules known as nuclear transport receptors (NTRs) (Cook et al., 2007, Görlich and Kutay, 1999). There is still a limited understanding of how the permeability barrier is formed and of how NTRs and FG-Nups orchestrate nucleocytoplasmic transport. *In vitro* equilibrium dissociation constant measurements (K_D_) between FG-Nups and most NTRs obtain high-affinity complexes (K_D_ in the nanomolar [nM] to micromolar [μM] range; for a review, see Aramburu and Lemke, 2017). A confounding issue has been the apparently paradoxical limit on how rapid the complex can in principle dissociate (k_off_ = K_D_·k_on_), a certain requirement for transport, which is at odds with how fast in cells NTRs can pass the permeability barrier (Kubitscheck et al., 2005, Milles et al., 2015, Sun et al., 2013, Tu et al., 2013, Yang et al., 2004).

We previously showed that the multivalent interaction between FG-Nups and NTRs takes place via the binding of multiple low-affinity binding sites, where, despite being hydrophobic, the F residues of the FG-Nups remain surface and solvent exposed and, thus, binding prone. This permits the Nup to engage with the NTR without undergoing a strong conformational change, ultimately giving rise to an “archetypal-fuzzy” complex. Distinct features of such a complex were the absence of substantial conformational changes in structure and dynamics on the length scale as detected by single-molecule fluorescence, molecular dynamics simulations, and nuclear magnetic resonance (NMR) by several labs for even different Nup⋅NTR complexes from different species (Hough et al., 2015, Milles et al., 2015, Raveh et al., 2016). In addition, kinetic measurements revealed very high association rate constants (∼10^9^ M^−1^s^−1^), which are on a par with the described values for diffusion-limited reactions between protein pairs. The permeability barrier also contains high concentrations (⪆ 50 mM) of FG-binding sites, so transport is essentially limited by breakage of individual FG-to-NTR-binding sites (k_off,individual_). Several unbinding events must take place in order for the NTR to cross the (>30 nm-thick) barrier. Combining our measurements for the K_D_ and the association rate constants for constructs with different numbers of motifs, we were able to account for the effects of multivalency in order to estimate k_off,individual_. The multivalency, combined with a high association rate constant, allows a tight complex to be formed between partners *in vitro*, despite a very high k_off,individual_. Thus, inside the permeability barrier, an NTR can migrate by a constant, rapid exchange of individual FG motif⋅NTR interactions, the multiplicity of which gives rise to a form of proofreading that contributes to high selectivity of the Nup⋅NTR interactions. Extensive computer simulations for short peptides give a visual idea of how such an exchange might occur (Raveh et al., 2016).

The archetypal-fuzzy-binding mechanism we observed appears distinct from other well-known binding mechanisms in which the IDP undergoes a substantial conformational change upon binding by, e.g., an induced fit or conformational selection process (Csermely et al., 2010, Wright and Dyson, 2009). To include complexes where conformational changes are not substantial, we here collectively term those coupled reconfiguration-binding mechanisms.

These results, obtained for a variety of FG-Nup⋅NTR systems and species, might seem at odds with recent crystal structures showing a 117-amino acid (aa) fragment of FG-Nup214 (and analogously also observed for yeast) (Koyama et al., 2017) apparently docked in an extended state to the exportin CRM1 (Port et al., 2015). Ultimately one might expect this to translate into overall lower association rate constants than for a fuzzy complex, since substantial conformational changes are likely to add to the energetic barrier for binding and, depending on the binding mechanism, proper orientation of the complex might be required. Ultimately, this brings us back to the transport paradox mentioned above, which drew our attention. Crystallization might trap a specific structure, such that the X-ray structure shows a snapshot of an otherwise dynamic state. However, it is also true that FG-Nup214⋅CRM1⋅RanGTP behaves biochemically differently from other FG-Nup⋅NTR interactions. For instance, in many biochemical approaches (such as size exclusion, ultracentrifugation, and pull-down assays), many FG-Nup⋅NTR complexes cannot be stably captured, due to the high dynamics of the complex, while for FG-Nup214, it has been known that a stretch in the C-terminal domain can form what appears to be a more stable complex with CRM1 (Hutten and Kehlenbach, 2006, Koyama et al., 2017, Labokha et al., 2013).

To better understand the different experimental observations, it is thus important to know if the crystal structure trapped a specific formation of an archetypal-fuzzy complex or if the FG-Nup214⋅CRM1 behaves distinctly compared to the large array of FG-Nup⋅NTR shown to form archetypal-fuzzy complexes. In this work, we combined multi-parameter single-molecule fluorescence resonance energy transfer (smFRET), kinetic measurements, and all atom molecular dynamics (MD) simulations to obtain a more comprehensive understanding of the interaction between the FG domain of Nup214 and the NTRs. We found that this cytoplasmic Nup extensively expands upon engaging with CRM1, thus undergoing a coupled reconfiguration-binding mechanism when interacting with the NTR. Unexpectedly, we found strong indications that this unique feature of the FG-Nup214⋅CRM1 interaction is encoded not only into CRM1 but also into the FG-Nup214 itself. Indeed, we found that FG-Nup214 can undergo a coupled reconfiguration-binding mechanism also when bound to the canonical import receptor Importinβ. We discuss these results in the context of how the NPC exploits conceptually different binding mechanism for spatially different functions.

In addition, we know that IDPs are major targets of post-translational modifications (PTMs), which can cause a change in the structure, net charge, stability, or binding surface, regulating the interaction with their binding partners (Bah and Forman-Kay, 2016). However, the possible role that O-linked β-N-acetylglucosamine (O-GlcNAc) modifications may play in the binding of FG-Nups to NTRs is poorly understood so far. Here we provide further insights into the FG-Nup⋅NTR interaction mechanism of O-GlcNAc-modified FG-Nups. We observed that glycosylated FG-Nups have a more expanded conformation than the unmodified form. However, despite detectable changes to the native state, our results showed that both fundamental binding mechanisms (archetypal-fuzzy and coupled reconfiguration-binding) were not substantially altered by glycosylation, fitting into the framework that the basic transport mechanism is very robust and conserved across species (Holt et al., 1987, Labokha et al., 2013, Zhu et al., 2016).

## Results

### Disordered FG-Nup214 Undergoes a Conformational Reconfiguration upon Binding with the NTR CRM1 and the CRM1⋅RanGTP Complex

**Figure 1:**
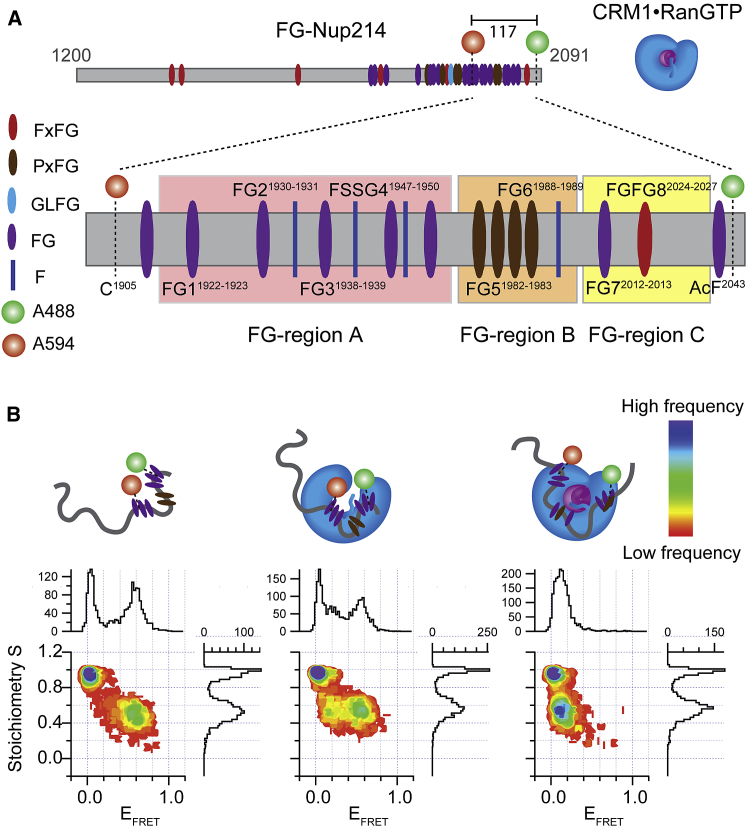
Conformational Change of FG-Nup214 upon Binding with CRM1 and RanGTP (A) Scheme of FG-Nup214 construct and FG-Nup214^117^, labeling sites, and CRM1⋅RanGTP complex. Eight FG motifs (FG1–FG8) binding to CRM1 pockets are indicated. The nomenclature of binding regions is adapted from Port et al. (2015). F residues are only shown in the zoom-in region. (B) S versus E_FRET_ histograms of 50 pM FG-Nup214 in the absence and presence of CRM1 and CRM1⋅RanGTP (from left to right, at 1 μM for both CRM1 and RanGTP concentrations). In the presence of CRM1, the E_FRET_ population (at S = 0.5) shows two populations corresponding to bound and unbound forms. In the presence of RanGTP, one distinct conformation with very low E_FRET_ is seen, suggesting a much more expanded conformation than in the unbound state. See also Figures S1 and S2.

We designed FG-Nup214 mutants for FRET measurements to probe the FG domain of Nup214 involved in CRM1 binding, as seen from the reported crystal structure (Port et al., 2015). FG-Nup214 was site-specifically labeled at a Cys introduced at residue position 1,905 with Alexa594-maleimide (acceptor dye) and at an incorporated unnatural aa *p*-acetylphenylalanine (AcF, aa 2,043) with Alexa488-hydroxylamine (donor dye; Figure 1A). SmFRET experiments were performed to monitor, among other parameters, changes in the FRET efficiency values (E_FRET_), which report on the distance-dependent changes in the efficiency of energy transferred from the donor to the acceptor dye due to, for example, a conformational change (refer to the Supplemental Information for details). The 2D S versus E_FRET_ plots, obtained from pulse-interleaved excitation (PIE) (Müller et al., 2005), show the populations according to the stoichiometry (S) of the dyes (y axis, S = 1 for molecules labeled with donor only; S = 0.5 for molecules containing a donor and an acceptor dye with a 1:1 ratio; and S = 0 for molecules labeled with acceptor only). Donor- or acceptor-only population can arise from dye photophysics like bleaching and/or incomplete labeling. The population at S = 0.5 is thus the one to monitor possible changes in the E_FRET_ values (x axis). E_FRET_ value shifting toward zero indicates an increase of the distance between the dyes, leading to a decrease in the efficiency of energy transferred from the donor to the acceptor dye.

Double-labeled FG-Nup214 molecules, in the absence of CRM1, showed a single FRET population with an E_FRET_ value of 0.6 (Figure 1B; Figure S1). The single peak was in line with the known fact that most IDPs are very dynamic, so that only an average distance of the rapidly fluctuating conformational ensemble was measured in smFRET (Mukhopadhyay et al., 2007). Surprisingly, if CRM1 was added in excess, we observed two FRET populations likely corresponding to an unbound (E_FRET_ = 0.6 and S = 0.5) and a bound (E_FRET_ = 0.2 and S = 0.5) state, as confirmed by photon distribution analysis (PDA; Figure S1A) (Kalinin et al., 2010). Apparently the unbound FG-Nup214 ensemble transitioned into an extended state upon binding CRM1. We note that, for signal-to-noise reasons, we could not further increase the concentration of CRM1 beyond ∼4 μM in smFRET experiments. The bound fraction was substantially higher populated in the presence of RanGTP due to higher affinity of the Nup214⋅CRM1⋅RanGTP complex. The E_FRET_ value of the bound population as well as the results from the PDA were in line with the results from the crystal structure showing the probed Nup214 segment docked to the CRM1 surface (Port et al., 2015). This behavior was distinct from all of the previously measured smFRET data for various other FG-Nup⋅NTR complexes, which did not show any substantial change in the peak positions upon binding (see below and Milles et al., 2015). A core signature of the archetypal-fuzzy complex is, thus, not fulfilled, and this directly speaks for a binding mechanism involving a large-scale conformational change in the ensemble of the IDP, i.e., a coupled reconfiguration-binding mechanism.

### Kinetic Rate Constants of FG-Nup214⋅CRM1 and FG-Nup214⋅CRM1⋅RanGTP Are Lower Than Other FG-Nup⋅NTR Binding Reactions

**Figure 2:**
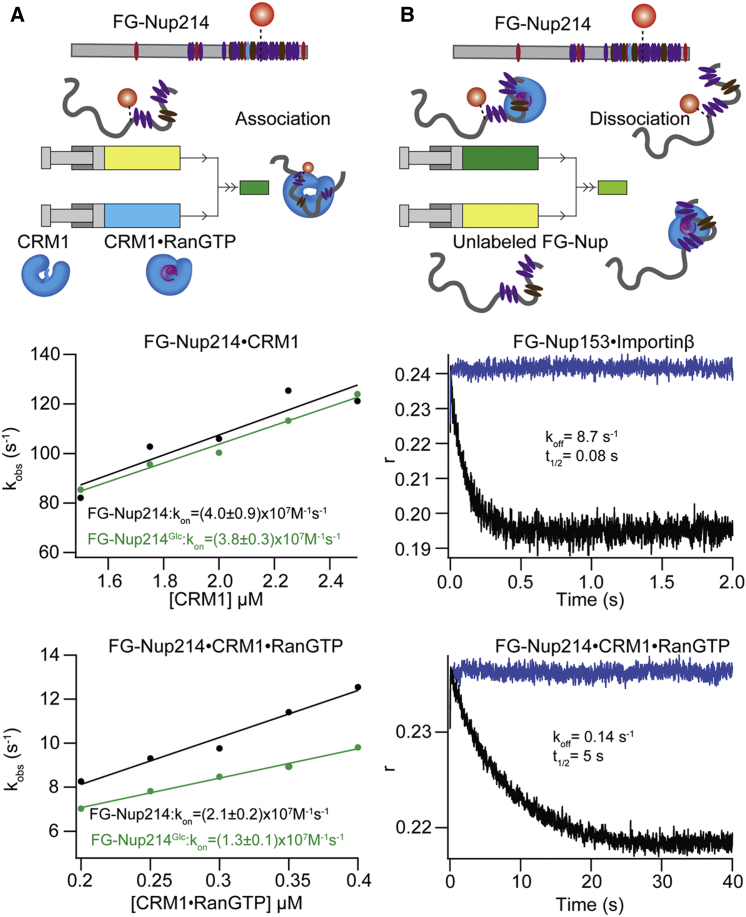
FG-Nup214 Has Lower Association and Dissociation Rate Constants Than FG-Nup153 (A) Kinetic association stopped-flow anisotropy measurements. Schematic drawings of mixing within stopped-flow fluorescence measurement are presented. Observed rates (k_obs_) from anisotropy association experiments were plotted for different CRM1 concentrations, and the data were linearly fitted to obtain the association rate constant (k_on_). (B) Dissociation experiment. Kinetic traces obtained from the dissociation of preformed FG-Nup⋅NTR complex upon rapid mixing with 100× (2 μM) excess of unlabeled FG-Nup. The k_off_ for FG-Nup153⋅Importinβ and FG-Nup214⋅CRM1⋅RanGTP was of 8.7 s^−1^ and 0.14 s^−1^, respectively. See also Figure S3.

Stopped-flow kinetic measurements monitoring complex formation using anisotropy under pseudo-first order conditions (i.e., >10-fold excess of the NTR) were used to extract the association rate constant (k_on_) of FG-Nup214 binding to CRM1 and CRM1⋅RanGTP (Figures 2A and S3, analogously to previously reported measurements for FG-Nup153⋅NTR; Milles et al., 2015). The observed rate (k_obs_) at different NTR concentrations was obtained by fitting the measured traces with a single exponential decay function. The k_on_ was obtained from the slope of the k_obs_ versus NTR concentration plots. We extracted a k_on_ = (4.0 ± 0.9) × 10^7^ M^−1^s^−1^ (Figures 2 and S3A) describing the binding of CRM1 with FG-Nup214. This was altered only slightly when RanGTP was pre-bound to CRM1 to (2.1 ± 0.2) × 10^7^ M^−1^s^−1^ (Figure 2A). Using donor signal change for a FRET-labeled sample rather than anisotropy gave roughly consistent results ((3.0 ± 0.5) × 10^7^ M^−1^s^−1^) (Figure S3C). Notably, these rate constants were roughly an order of magnitude lower than the ones reported for most of the other FG-Nup⋅NTR interactions (∼10^9^ M^−1^s^−1^) (Milles et al., 2015).

We further studied the FG-Nup⋅NTR-binding mechanism by performing dissociation kinetic measurements (Figure 2B). The rate of complex dissociation obtained using 100× excess (2 μM) of unlabeled FG-Nup showed a k_off_ of 8.7 and 0.14 s^−1^ for our FG-Nup153⋅Importinβ and FG-Nup214⋅CRM1⋅RanGTP complexes, respectively, which corresponds to a ∼60-fold difference in the complex half-life of 0.08 and 5 s under the same concentration of unlabeled FG-Nup (see Supplemental Information).

### Glycosylated Nups Maintain Their NTR-Binding Mechanisms

In contrast to yeast, metazoan Nups are highly glycosylated. As NPC transport appears robust across species, the exact role of this omnipresent PTM remains to be better understood. Having identified two conceptually different binding mechanisms between NTRs and unglycosylated FG-Nups, we studied the influence of this PTM in the FG-Nup⋅NTR binding. We *in vitro* glycosylated FG-Nup214 (FG-Nup214^Glc^) and FG-Nup153 (FG-Nup153^Glc^) following a procedure previously developed for FG-Nup98 (Labokha et al., 2013). The *in vitro* glycosylation of FG-Nups was confirmed by SDS-PAGE, western blots, and peptide digest mass spectrometry (Figure S4).

**Figure 3:**
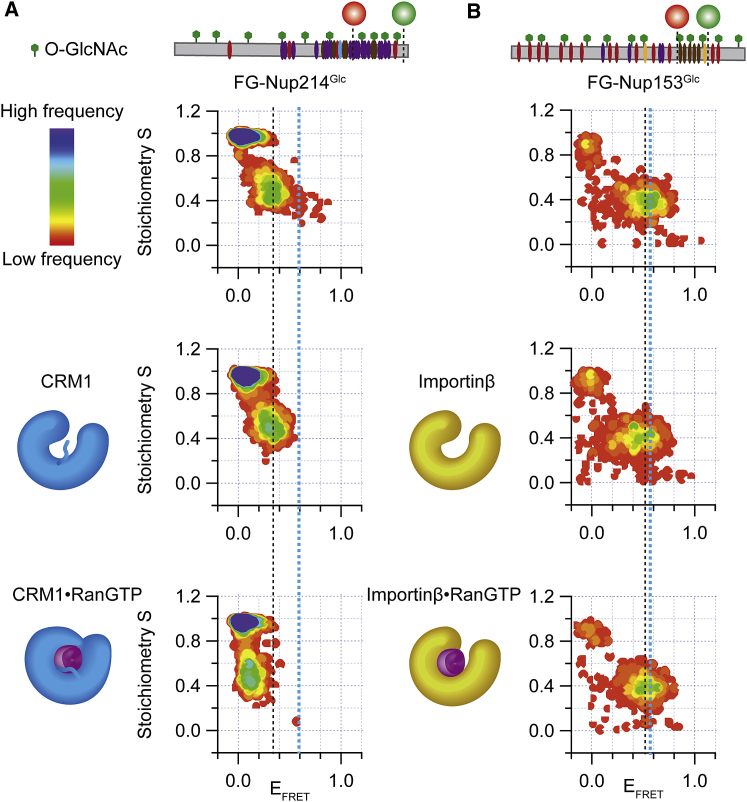
Conformational Features of Glycosylated FG-Nup upon Interaction with NTR (A) E_FRET_ versus S histograms of FG-Nup214^Glc^ in the absence and presence of CRM1 and CRM1⋅RanGTP (from top to bottom, at 1 μM for both CRM1 and RanGTP concentrations). In the presence of CRM1, the E_FRET_ population had a similar E_FRET_ value to unbound FG-Nup. In presence of RanGTP, one distinct population with very low E_FRET_ was seen. The E_FRET_ value was remarkably similar to the bound unglycosylated case (compare to Figures 1 and 5). (B) S versus E_FRET_ histograms of FG-Nup153^Glc^ in the absence and presence of Importinβ and Importinβ⋅RanGTP (from top to bottom, at 1 μM for both Importinβ and RanGTP concentrations). In particular for FG-Nup214^Glc^, a lower E_FRET_ compared to the unglycosylated state (black dotted line versus blue dotted line) in the unbound form was detected, indicating that the glycosylated Nup is more extended. However, glycosylated and unglycosylated FG-Nups behave remarkably similarly in their respective binding modes, indicating that glycosylation only mildly tunes the binding mechanism between FG-Nups and NTRs. See also Figures S1 and S4.

We performed smFRET experiments under the same conditions of Figure 1 by using FG-Nup214^Glc^. Figure 3 shows that, in particular, FG-Nup214^Glc^ (E_FRET_ = 0.3; FG-Nup153^Glc^ E_FRET_ = 0.5) had lower E_FRET_ compared to the unglycosylated FG-Nup in the unbound form, indicating expansion upon glycosylation. In contrast to the unglycosylated form, FG-Nup214^Glc^ in the presence of CRM1 yielded only a single E_FRET_ population, as validated by PDA (Figure S1A), which was similar to its unbound form (Figure 3A), indicating a reduced affinity of the complex (so that no bound fraction was populated under the chosen experimental conditions). In the presence of CRM1⋅RanGTP, we detected again a single population virtually identical to the unglycosylated and bound state. In contrast, FG-Nup153 yielded similar smFRET signals in the presence of Importinβ for both the glycosylated and unglycosylated forms (Figure 3B).

We then performed all measurements for the labeled fragment (117 aa, termed FG-Nup214^117^), which probed the same region. Despite the full-length FG-Nup being approximately 55 kDa larger and containing 45 more Fs, the E_FRET_ values measured for the fragment (FG-Nup214^117^) and the same region probed for full-length FG-Nup214 were remarkably similar in the glycosylated and unglycosylated forms (Figures 3A, S4A, and S4B). This showed that the detected effect was largely encoded into the region sandwiched between the FRET labels.

We also compared the kinetics for FG-Nup214^Glc^ and FG-Nup153^Glc^ (Figure 2; Figures S3D–S3F) binding to NTRs. We obtained a k_on_ = (3.8 ± 0.3) × 10^7^ M^−1^s^−1^ for the FG-Nup214^Glc^⋅CRM1 interaction, (1.3 ± 0.1) × 10^7^ M^−1^s^−1^ for the FG-Nup214^Glc^⋅CRM1⋅RanGTP interaction, and (8.5 ± 1.1) × 10^8^ M^−1^s^−1^ for the FG-Nup153^Glc^⋅Importinβ interaction. These data indicated that there was no substantial difference between glycosylated FG-Nup and unglycosylated FG-Nup in terms of association rate constants with NTRs.

### MD Simulations Support a Coupled Reconfiguration-Binding Mechanism between FG-Nup214^117^ and CRM1⋅RanGTP

Previous MD simulations were key in understanding the molecular architecture of the dynamic FG-Nup⋅NTR complexes (Milles et al., 2015, Raveh et al., 2016). To elucidate the role of different FG repeats and CRM1-binding pockets in determining the binding of the FG-Nup214^117^ to CRM1⋅RanGTP, we employed all-atom MD simulations. After reconstructing the parts of the complex that X-ray crystallography did not resolve (see the Experimental Procedures) (Port et al., 2015), FG-Nup214^117^ was simulated in isolation and in complex with the CRM1⋅RanGTP heterodimer. We performed all simulations with AMBER99sb^∗^-ILDN and TIP4PD (Piana et al., 2015) and with the Kirkwood-Buff protein force field (Ploetz et al., 2010), both of which have been shown to yield dimensions of unfolded proteins or IDPs in line with experimental findings (Mercadante et al., 2015, Piana et al., 2015).

The analysis of the end-to-end distance (R_E_) and radius of gyration (R_G_) of FG-Nup214^117^ revealed a remarkable difference between the bound and unbound states for the two force fields (compare Figures S5A and S5C with S5B and S5D). Unbound FG-Nup214^117^ mostly assumed semi-compacted conformations when compared to the bound segment (Figures S5A–S5D). The expansion of the bound FG-Nup214^117^ directly agreed with the E_FRET_ increase observed upon binding of FG-Nup214^117^ to CRM1⋅RanGTP (Figure 1B). Our simulations confirmed that F1915, which lies at the N terminus of the FG-Nup214^117^ fragment, did not interact with CRM1, as also previously reported (Port et al., 2015). The unbound state included only a minority of conformers with R_E_, R_G_, and α-helical content (Figure S5E) overlapping with the range sampled in the bound state. Notably, the sequence-based prediction of intrinsic disorder for FG-Nup214^117^, as profiled by a range of disorder predictors, suggested a lower tendency for disorder at the termini (Figure S5F). Concordantly, the X-ray crystal structure of the FG-Nup214^117^ bound to CRM1⋅RanGTP revealed a tendency to assume an α-helical conformation (Port et al., 2015). Our MD data corroborated the α helix propensity of FG3 in these regions in both bound and unbound states (25% versus 13%) (Figure S5G). We also found another stretch lying at the C terminus of FG-Nup214^117^, ahead of F2024-G2025 (FG8), to form an α helix in both bound (∼35%) and unbound (∼15%) states (Figure S5G). Hence, both the FG3 and FG8 motifs experience strong conformational restraints because of the immediately flanking secondary structure elements of the otherwise disordered peptide.

### MD Simulations Reveal Dynamics of FG Repeats in the FG-Nup214^117^⋅CRM1⋅RanGTP Complex

**Figure 4:**
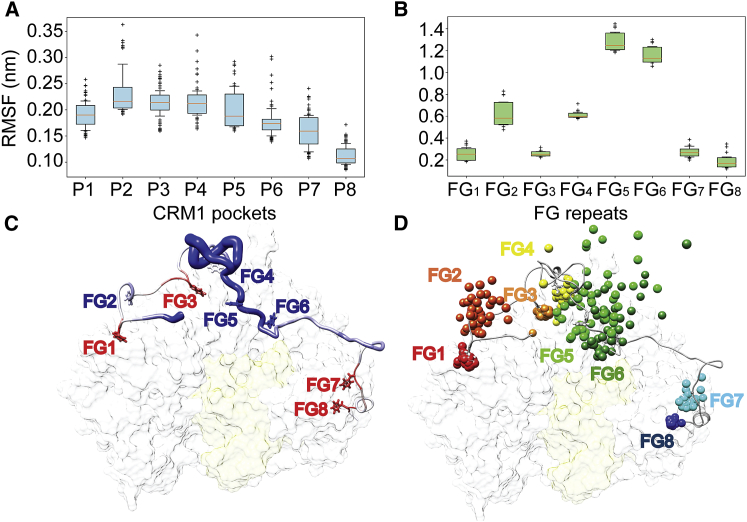
Dynamics of FG Repeats in the FG-Nup214^117^⋅CRM1⋅RanGTP Complex (A) Root-mean-squared fluctuations (RMSFs) of the CRM1 residues forming the pockets binding the FG-Nup214^117^ FG repeats. (B) RMSFs of the FG repeats binding the CRM1 pockets (P1–P8) shown in (A). Each box indicates the range (interquartile range [IQR]) between the lower (Q1) and upper (Q3) quartile whereas the solid line represents the distribution’s median. The whiskers report values that fall below Q1–Q1.5^∗^IQR or above Q3 + 1.5^∗^IQR. Outliers are shown as crosses below or above whiskers. (C) Representative structure of the FG-Nup214^117^⋅CRM1 complex. The width and coloring of the FG-Nup214^117^ backbone is proportional to the B factor as obtained from MD simulations, with blue for a low and red for a high B factor. The labels of the different FG repeats refer to the FG1–FG8 range described by Port et al. (2015). (D) Dynamics of FG-Nup214^117^ FG repeats bound to CRM1. The positions of the Cζ atoms, which are part of the F rings, are represented as spheres in frames collected every 5 ns along the simulated trajectories. Spheres of different FG repeats are colored from red (N terminus) to blue (C terminus) as follows: F1922 (part of FG1), red; F1930 (part of FG2), dark orange; F1938 (part of FG3), orange; F1947 (part of FG4), yellow; F1982 (part of FG5), green; F1988 (part of FG6), dark green; F2012 (part of FG7), cyan; and F2024 (part of FG8), blue. See also Figures S5 and S6 and Movie S2.

In MD simulations of the FG-Nup214^117^⋅CRM1⋅RanGTP complex, we observed a diverse dynamical behavior for the different pockets of CRM1 and interacting FG repeats. We observed the root-mean-squared fluctuations (RMSFs) of the CRM1 P1- to P8-binding pockets of the NTR (Port et al., 2015) to be significantly lower with the simulated fragment docked (Figure 4A), which is in line with the experimental B factors from previously collected X-ray crystallographic data (Port et al., 2015) (Figures S6A and S6B). The RMSF of the FG repeats (FG1–FG8) showed a similar trend, with FG1 and FG7/FG8 having low fluctuations and the FG repeats lying in the middle of the chain (FG4, FG5, and FG6) being characterized by a higher degree of dynamics (Movie S1). In particular, the hydrogen bond between the backbone carbonyl and amide groups of T1981 and G1984, respectively, which have been suggested to stabilize the docking of FG5 into P5 of CRM1 (Port et al., 2015), were not retained during the simulations (Figures S6C–S6F). It is worth noticing that the RMSF values recorded for FG2 suggested intermediate dynamics, compared with the FG repeats at the termini (FG1, FG7, and FG8) and FG5 or FG6 (Figure 4B), but were still incompatible with a stable binding of CRM1. This was in line with the previous observation that FG2 (F1931–G1932) interacted with CRM1 only superficially (Port et al., 2015). Conversely, FG3 (F1938–G1939) remained located in a deep pocket of CRM1, resulting in comparably little fluctuations. Fluctuations of this FG repeat were further diminished by a short α-helical segment involving this ^1937^SFGE^1940^ stretch. Thus, only a distinct subset of FG repeats (FG1, FG3, FG7, and FG8) stably interacted with the NTR. For the others (FG2, FG4, FG5, and FG6), which retained highly fluctuating dynamics also in the bound state, we estimated k_off,individual_ in the submicrosecond range and K_D,individual_ in the range of 0.1–0.7 mM based on the bound and unbound fractions (Figure S6F). This range is comparable to the local K_D,individual_ for FG-Nup153⋅Importinβ (Milles et al., 2015, Tu et al., 2013), and it underscores the dynamic nature of these FG motifs.

### The FG-Nup Rather Than the NTR Dictates the Binding Mechanism

**Figure 5:**
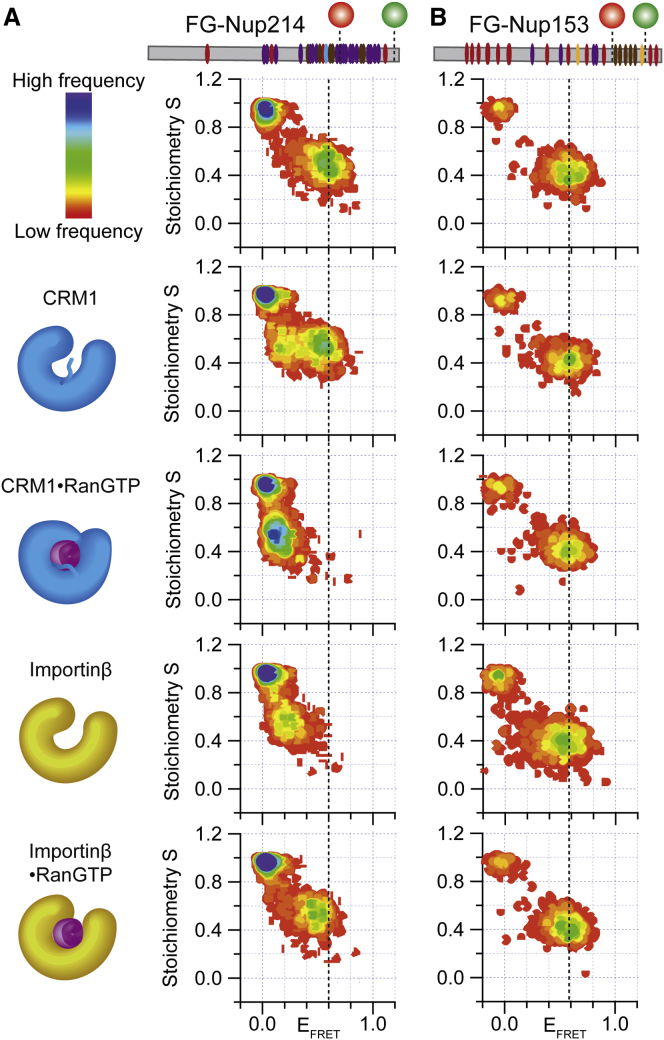
Conformational Feature of Different NTRs in the Presence and Absence of Different NTRs Probed by smFRET (A and B) S versus E_FRET_ histograms of (A) FG-Nup214 and (B) FG-Nup153 in the absence and presence of different NTRs (with/without RanGTP, at 1 μM for both NTR and RanGTP concentrations). Left panel rows 1–3 are repeated from Figure 1 for comparative reasons. The black dotted line visualizes the shift of the E_FRET_ peak. The smFRET data show a distinct conformational feature of FG-Nup214 upon interaction with CRM1, Importinβ, and the CRM1⋅RanGTP complex. In contrast to FG-Nup214, FG-Nup153 does not undergo any conformational change, as detectable by smFRET, in the presence of Importinβ or CRM1. See also Figure S7.
(2) FG-Nup sequences contain low-complexity regions and are evolutionarily not well conserved. As such, insights from bioinformatics analysis to reveal specific design features or assign motifs within FG domains are still limited compared to other classes of proteins (Ando et al., 2013, Schmidt and Görlich, 2015, Yamada et al., 2010). This is highlighted by the facts that physiological NPCs can tolerate even massive deletions of FG-Nups and encode a high level of functional redundancy (Hülsmann et al., 2012, Strawn et al., 2004). As such, mutational studies targeting only a few sites are unlikely to have a strong effect on FG-Nup function. However, prompted by the simulations, which pointed toward the relevance of α helicity in the unbound and bound states, we aimed to perturb any residual helical structure using helix-breaking mutations (by inserting proline) (Bienkiewicz et al., 2002, Schulman and Kim, 1996). We assessed the binding of FG-Nup214^117^ proline mutants A1927P/A2017P and A1927P/A2017P/A2019P, as shown in Figure 6A, to the CRM1⋅RanGTP complex using smFRET spectroscopy. Bound and unbound subpopulations were observed by smFRET measurements, and these subpopulations were directly monitored as a function of CRM1 concentration to obtain a K_D_ of the bound complex (Figure 6B). Although A2017^FG-Nup214^ interacted with Y105^CRM1^ as shown by (Port et al., 2015), the double-proline mutant (A1927P/A2017P) showed only a slight increase in K_D_ value (from 35 ± 5 nM to 53 ± 7 nM). However, when A2019, which lies within the α helix structure, was further mutated (giving the triple mutant A1927P/A2017P/A2019P), K_D_ increased 5-fold over that for wild-type (WT) FG-Nup214^117^ (K_D_ = 222 ± 19 nM), in agreement with predictions by our MD simulations.

Our MD data indicated that specifics about the binding mechanism were encoded into the FG-Nup rather than into CRM1. To gain experimental support for this, we followed two approaches:

(1) The smFRET data (Figure 1B) showed a distinct conformational change of FG-Nup214 upon interacting with CRM1 and CRM1⋅RanGTP that did not occur in other pairs we tested (Figure 5B). We now extended our smFRET study by comparing the binding of FG-Nup153 to CRM1, as well as FG-Nup214 together with Importinβ and both with RanGTP (Figure 5A). Similar to CRM1, Importinβ also contains HEAT repeats and is a prototypical import receptor (Cook et al., 2007). Importantly, we detected a significant shift in the E_FRET_ value of FG-Nup214 when adding Importinβ, suggesting that it populated more expanded conformers. This observation is qualitatively similar to the results obtained for the FG-Nup214⋅CRM1 and FG-Nup214⋅CRM1⋅RanGTP complexes. In stark contrast to FG-Nup214, FG-Nup153 did not undergo any detectable conformational change in the presence of either Importinβ or CRM1, highlighting the differential behavior of FG-Nup153 and FG-Nup214 and the role of the Nups in influencing the binding mechanism to NTRs.

**Figure 6:**
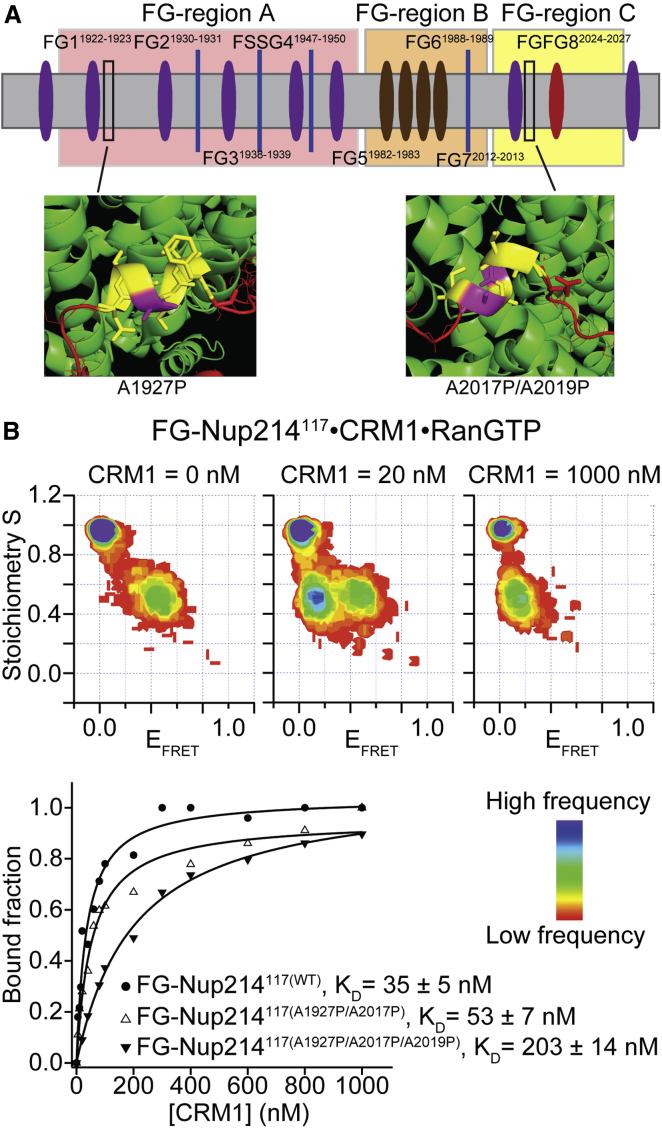
K_D_ Determination of FG-Nup214^117^ Proline Mutant CRM1⋅RanGTP Complex (A) Scheme of FG-Nup214^117^ proline mutant construct and mutation sites of FG-Nup214^117^(A1927P/A2019P) and FG-Nup214^117^(A1927P/A2017P/A2019P). (B) S versus E_FRET_ histograms of FG-Nup214^117^ in the presence of different concentration of CRM1 in the presence of (1 μM) RanGTP. In the presence of a low concentration of CRM1, two FRET populations (bound and unbound) were observed, and the fraction of these populations (low E_FRET_, bound complex; high E_FRET_, unbound complex) was directly monitored as a function of CRM1 concentration to obtain K_D_ of the bound complex, as shown in the lower panel.

## Discussion

Active transport through the NPC requires at least three distinct steps: docking to the NPC, passage through the permeability barrier, and undocking. Export proceeds analogously in the other direction. Since electron tomography has provided a very detailed snapshot of the NPC scaffold and the stoichiometry of Nups constituting the NPC is known, the concentration of F residues in the roughly 30-nm-wide barrier is very high (≳ 50 mM; for reviews, see Aramburu and Lemke, 2017 and Beck and Hurt, 2017). This barrier can be passed within ∼3–5 ms, as known from single-particle tracking studies that follow the trajectory of an individual NTR or cargo molecule and can, in principle, even distinguish docking, barrier passage, and undocking steps (Kubitscheck et al., 2005, Milles et al., 2015, Sun et al., 2013, Tu et al., 2013, Yang et al., 2004). Molecularly, barrier passage must thus require rapid formation and rapid breakage of Nup⋅NTR bonds.

Inside the permeability barrier, the concentration of Fs is so high that, even for a typical complex k_on_ of 10^5^ M^−1^s^−1^, the NPC transport time will be almost independent of the motif k_on,individual_. However, a naive estimate of 1 s^−1^ for k_off_, based on typical *in vitro* K_D_ measurements of isolated Nup⋅NTR complexes (100 nM), and a k_on_ not exceeding 10^7^ M^−1^s^−1^ cannot explain how even a single Nup⋅NTR bond can be broken during the 5 ms transport time. Recently, we found an ultrafast binding modality of FG-Nups involving multiple low-affinity binding motifs engaging in a highly dynamic manner with the different binding sites of the NTRs (Milles et al., 2015). The observed binding mechanism, termed archetypal-fuzzy, which was found for diverse NTRs and FG-Nups across different species (human and yeast; Hough et al., 2015, Milles et al., 2015, Raveh et al., 2016), forms a dynamic archetypal-fuzzy complex in which any adopted conformation of the unbound FG-Nup is binding prone and can bind NTRs without observing a detectable change in the multiple rapidly interconverting conformations upon binding (shown as no change in the E_FRET_ values) (Milles et al., 2015). Remarkably, we observed association rate constants approaching the theoretical diffusion limit. This allows a higher k_off_ estimate inferred from the observed K_D_. However, it is critical to take multivalency into account. FG-Nups like FG-Nup153 or FG-Nup214 have 60 and 62 Fs, respectively, and NTRs like Importinβ and CRM1 have 19 and 21 HEAT repeats tentatively capable of binding FG. Taking both the measured diffusion limited k_on_ and multivalency into account can bring estimates of k_off,individual_ orders of magnitude higher than the measured global k_off_, and it can explain how NTRs can pass through the permeability barrier of the NPC in millisecond timescales.

Such a fuzzy complex-binding mechanism could not have been deduced easily from available FG-Nup (peptide)⋅NTR crystal structures (Bayliss et al., 2000a, Bayliss et al., 2000b, Bayliss et al., 2002a, Bayliss et al., 2002b) as those rather showed snapshots of specific trapped states. Thus, a major question that now arose was, how does the detailed crystal structure showing a 117-aa Nup214 fragment docked to the CRM1⋅RanGTP complex fit into this picture? In this paper, we show that at least two distinct binding mechanisms exist on how an FG-Nup can interact with an NTR, i.e., an archetypal-fuzzy one and a coupled reconfiguration-binding mechanism.

### Coupled Reconfiguration Binding versus Archetypal-Fuzzy Complex Formation in the NPC

In this work, we compared side by side the FG-Nup153⋅Importinβ with the FG-Nup214⋅CRM1 complex. Our smFRET data indicated that FG-Nup214 in the presence of CRM1 and the CRM1⋅RanGTP complex adopts a specific structure that is more extended (i.e., lower FRET and larger R_E_ in MD; Figures 1 and S5) than the average conformation of the disordered native ensemble of FG-Nup214 on its own. Notably, the probed 117-aa FG region behaves in this respect similarly independent if studied on its own, or within the context of the 699-residue-long region of the FG domain of Nup214 (Figures 1 and S4A). The detected differences in the smFRET data in the absence of RanGTP were due to a lower affinity of the short fragment for CRM1 (Figure S1). In the presence of CRM1 and RanGTP, a clear and distinct FRET shift occurs for both, the FG-Nup214 short fragment and full-length FG domain. This strong shift in the E_FRET_ signal was absent in all previously measured Nup⋅NTR complexes (Milles et al., 2015), and it argues against formation of an equally fuzzy complex.

Previously, we observed formation of a fuzzy complex characterized by extremely high association rate constants. Interestingly here, despite similar sizes and numbers of Fs, we did indeed observe a lower k_on_. Perhaps more biologically interesting is any potential difference in the k_off,individual_ that can be inferred from our experiments, since this is critical to permeability barrier passage time. The complex half-life of FG-Nup214⋅CRM1⋅RanGTP is more than 60-fold higher than FG-Nup153⋅Importinβ. k_off,global_ is thus more than one order of magnitude lower in the case where structural rearrangements take place. This appears at odds with experimental data that suggest coupled folding and binding processes are generally typified by faster dissociation rates than those without structural rearrangement (Huang and Liu, 2009, Shammas et al., 2012). However, this comparison is with pairs of structured proteins; data for fuzzy complexes are too limited for comparison as of yet. It is also important to note that only k_off,global_ is accessible to us experimentally. This is much lower than any estimated k_off,individual_, and highly dependent on the number of, and distances between, F-binding sites (see the Supplemental Experimental Procedures). Extreme caution should also be exercised when comparing rate constants for two different pairs of proteins. Our conclusion of two differential binding mechanisms is based on the synergistic results from different experimental technologies and evidences, rather than only from kinetic measurements.

MD simulations also captured the same conformational change of FG-Nup214 upon interacting with CRM1⋅RanGTP, providing further evidence that FG-Nup214 undergoes a conformational reconfiguration, which is substantially different from the other tested FG-Nup⋅NTR cases investigated before (Milles et al., 2015). Notably, the simulations (Figures S5A–S5E) show an overlapping conformational space both in terms of extent of collapse and residual secondary structure between the unbound and bound state ensembles. More specifically, already in the unbound state, FG-Nup214 samples to a minor extent also more extended conformations and structures with partial α helicity, both of which appear to be relevant for FG-Nup214⋅CRM1⋅RanGTP complex formation. We next tested with our MD simulations if anchorage and conformational propensity of FG-Nup214 at the distant CRM1 pockets might cause the observed expansion of the IDP ensemble. Indeed, the bound conformation showed differential dynamics across the binding interface, with lower dynamics and consequently stronger binding found for the moieties that anchor the terminal FG repeats of the simulated FG-Nup214 fragment. The large central part of the bound FG-Nup214 fragment seems to instead play a minor role in defining formation and stability of the complex. This dynamic and very heterogeneous central region also included the three repeats FG4–FG6, which were among those well resolved within CRM1 pockets in the X-ray structure (Port et al., 2015) (see Movie S2 and Figures S6C–S6F). This underlines that, despite all FG regions being resolved in the crystal structure, their dynamic behavior can be completely different. We conclude that coupled reconfiguration binding in the case of FG-Nup214 and CRM1 also includes fuzzy and transient FG⋅CRM1 interactions, with local K_D,individual_ similar to those of FG-Nup153⋅Importinβ. Movie S2 visualizes how an FG motif in the middle of the 117-aa fragment constantly binds and unbinds on the very short nanosecond timescale, in agreement with ultrafast kinetics. The termini of the 117-aa Nup214 appear largely responsible for locking the peptide onto the CRM1 surface. The co-existence of these two binding modes in very close proximity probably compensates for the entropic penalty that may be generated from the reduced conformational freedom of FG-Nup214 upon CRM1 binding (Marlow et al., 2010, Tzeng and Kalodimos, 2012).

### Spatial Segregation of Distinct Binding Mechanisms in the NPC

In the central channel of the NPC, where the permeability barrier is formed by high densities of FG-Nups, a tight clamping mechanism with its associated kinetics is not favorable, because it would reduce the transport efficiency of cargo passage substantially. This points to a unique role of the FG-Nup214⋅CRM1 interaction in the NPC mechanism in line with previous observations (Hutten and Kehlenbach, 2006, Labokha et al., 2013). FG-Nup214 localizes to the cytoplasmic side of the NPC, and it is most likely not a key component of the permeability barrier of the central channel at the NPC but rather may have other functionalities. For example, the N-terminal folded domain of Nup214 has been shown to be required for the recruitment of the DEAD-box helicase Ddx19 involved in the messenger ribonucleoprotein particles (mRNPs) remodeling (Napetschnig et al., 2009). Moreover, it has been suggested that Nup214 takes part in the last steps of the nuclear export of cargoes (Kehlenbach et al., 1999). In addition, depletion of Nup214 has been reported to cause the inhibition of some CRM1 export cargoes (Bernad et al., 2006), and RNAi downregulation of FG-Nups in S2 *D. melanogaster* cells showed that different FG domains play distinct roles in the nucleocytoplasmic transport (Sabri et al., 2007).

Altogether, we can speculate that free CRM1 can bind and unbind and move rapidly through Nups in the central channel. However, when CRM1 is forming part of the export complex (bound to RanGTP), it will specifically and tightly bind to the C-terminal region of FG-Nup214 once it reaches the cytoplasmic face of the NPC and form a longer-lived complex, which is in good agreement with our dissociation kinetic experiment. In this way, CRM1⋅RanGTP may dock on FG-Nup214 at the C-terminal position, and they may get in close proximity to Ran-binding protein 2 (RanBP2), which has also been shown to bind strongly to CRM1⋅RanGTP with two FG regions 300 aas apart (Ritterhoff et al., 2016), in order to undergo GTP hydrolysis and cargo release (Port et al., 2015). In addition, competition experiments have shown that the Ran-binding protein RanBP3 that facilitates the formation of the export complex is also able to displace FG-Nup214 from the CRM1⋅RanGTP complex (Port et al., 2015). Further studies showed that two FG regions located at the disordered domain of Yrb2p, the yeast homolog of RanBP3, bind to the C- and N-terminal sites of Xpo1p, the yeast homolog of CRM1 (Koyama et al., 2014). This might indicate that RanBP3 interacts with CRM1⋅RanGTP in a similar binding mechanism as FG-Nup214. Thus, CRM1 release from FG-Nup214 is subject to tight biochemical control instead of ultrafast spontaneous dissociation.

### Coupled Reconfiguration Binding of Nup214 Is Not Unique to CRM1 Complex Formation

The NPC is made from several FG-Nups, but what sequence characteristics give rise to a specific function is not well understood, and, indeed, minimal functional pores can even be built from few FG-Nups (Hülsmann et al., 2012, Strawn et al., 2004). FG-Nups are only poorly evolutionarily conserved (Ando et al., 2013, Denning and Rexach, 2007, Schmidt and Görlich, 2015, Yamada et al., 2010), and many have large low-complexity regions. Identifying unique features in these large disordered molecules is thus a complex venture. Our MD simulations showed that one factor determining tight binding of FG repeats appears to be the formation of short helical structures adjacent to the FG (FG3 and FG8) in both bound and unbound states. This suggests that anchorage, i.e., coupled reconfiguration binding, might only to a minor extent depend on the nature of the CRM1 pockets but primarily be achieved through unique characteristics of the Nup214, such as a sequence-encoded propensity for transient secondary structure. Such a propensity in exploring α-helical conformations that would lock more tightly FG repeats at the terminal ends of the fragment bound to CRM1 is predicted based on the FG-Nup214 sequence as well as on the sampling of the bound and unbound state dynamics (Figures S5 and S6). The relevance of α-helical propensity in regions next to the two terminal FG motifs was further supported by our proline mutational analysis (Figure 6). While further experiments and ideally more systems need to be identified and compared, our current data put forward the notion that the FG-Nup and its sequence propensity more strongly dictate the binding mechanism than the nature of the NTR pockets. Indeed and unexpectedly, we observed that FG-Nup214 undergoes a conformational change also when binding to Importinβ (Figure 5A), yielding even a very similar E_FRET_ value as when binding to CRM1. In the Supplemental Experimental Procedures, we show further experiments that reveal that we can also detect time- and concentration-dependent aggregation phenomena in the FG-Nup⋅NTR complex (Figure S7), highlighting that, in such multivalent systems, more complex processes (such as phase separation) can occur as well. However, the very low concentrations (in the picomolar [pM] range) used in our single-molecule assays provide a means to deal with this complexity under the chosen experimental conditions.

### The Coupled Reconfiguration-Binding Mechanism and the Archetypal-Fuzzy Complex Formation Are Robust with Respect to Glycosylation

Recent studies showed that glycosylation, an omnipresent PTM in metazoan NPCs but not yeast, may play a role in regulating nucleocytoplasmic transport, and FG-Nup214 has been shown to be heavily glycosylated in the NPC (Favreau et al., 1996, Khidekel et al., 2007, Zhu et al., 2016). If and how glycosylation affects the FG-Nup⋅NTR-binding mechanism is still not well understood. From the smFRET measurement shown in Figure 3, we observed that FG-Nup214^Glc^ and FG-Nup153^Glc^ in the unbound state are more expanded. This expansion upon glycosylation is likely due to an increased steric hindrance by the multiple bulky sugar chains, an effect that here overcompensates any impaired solvation of the semi-collapsed Nup214 (Cheng et al., 2010). Despite the conformational change upon glycosylation, a shift in the FRET population of FG-Nup214^Glc^ upon binding to CRM1⋅RanGTP was detected (Figures 3 and S4). This suggests that, while glycosylation might tune the affinity of the complex, the binding mechanism itself is robust. Analogously, we did not observe a substantial change in the data for the complex formation of FG-Nup153 to Importinβ (Figure 3B). We conclude that, for the study of the binding mechanism, unglycosylated proteins appear to be a reasonable mimic.

**Figure 7:**
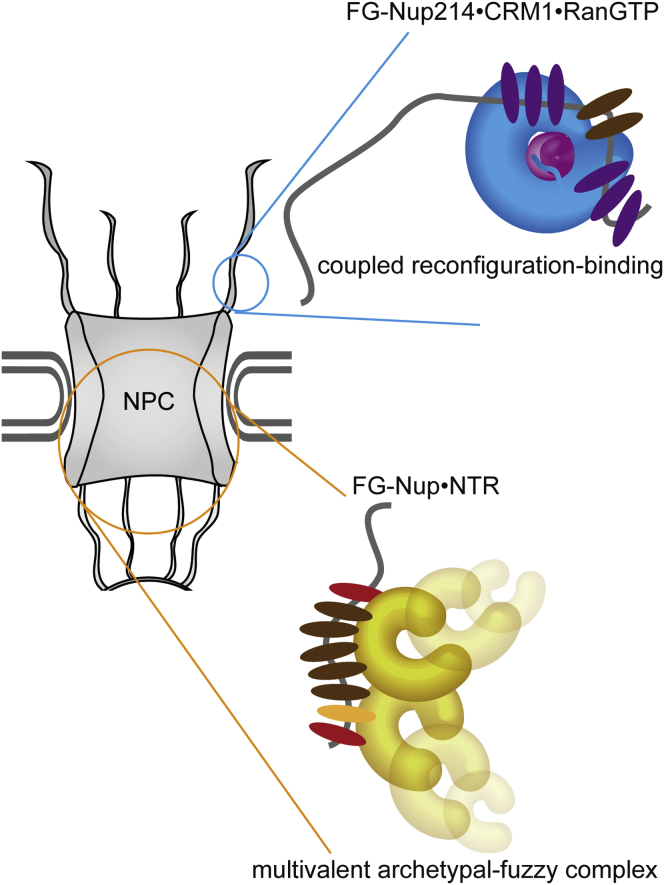
Differential Binding Modes of FG-Nup•NTR Interactions Disordered FG-Nup214 undergoes a coupled reconfiguration binding with the NTR CRM1⋅RanGTP complex, in contrast to the previously reported archetypal-fuzzy binding mechanism of FG-Nup153⋅Importinβ, indicating that variations in the type and extent of individual FG⋅NTR interactions can drastically change the mechanism of binding between the FG-Nup and its NTR.

We summarize that in the NPC at least two fundamentally different binding mechanisms can exist between FG-Nups and NTRs (Figure 7). Formation of archetypal-fuzzy complexes is associated with fast yet selective transport through highly concentrated FG-rich channels. Potentially FG-Nup214, which forms a complex with CRM1⋅RanGTP by a coupled reconfiguration-binding mechanism, can help to achieve a stable spatial localization that helps undocking processes.

From an IDP biophysics perspective, our study sheds light on the diverse dynamics of IDPs, and it provides an example of how different FG-rich disordered stretches can use completely different binding mechanisms to bind to NTRs in order to gain a new functionality. However, it is not yet clear what is the basis of such biphasic behavior, whether it is subtleties in sequence, motif spacing, or other dynamic parameters. A better understanding of the architectural design of FG-Nups and what gives a certain sequence a specific role is currently complicated by our generally limited understanding of sequence space design. In particular, the high enrichment of low-complexity regions in FG-Nups and many other IDPs, which in many cases are also not evolutionarily conserved, makes identifying specific key residues in fact unlikely, as it is rather a collective property emerging for a specific area or even during assembly of a larger complex that can give rise to unique function. Larger systematic efforts in which many parameters are varied to find the essential ingredients that make an FG-Nup sequence special will be needed. This will be complicated but also intriguing because physiological NPCs can tolerate massive deletions of FG-Nups and encode a high level of redundancy.

## Experimental Procedures

### Protein Expression, Purification, Glycosylation, and Labeling

Proteins were expressed in *E. coli* BL21 DE3 AI cell and purified using standard Nickel affinity and size exclusion chromatography purification procedures. *In vitro* glycosylation and labeling were performed as described in the Supplemental Experimental Procedures.

### smFRET Measurement

The smFRET experiments were performed using a home-built confocal-based microscope, and the data analysis was performed by using a custom written Igor Program (Wavemetrics, USA). The detailed information is described in the Supplemental Experimental Procedures.

### Fluorescence Stopped-Flow Measurement

The kinetic experiments were performed using stopped-flow fluorescence spectroscopy (SFM-3000, Bio-logic, France) with the uFC-08 micro-cuvette accessory. Excitation was performed with a custom polarized LASER excitation source (532 nm), and polarized emission was detected using emission filters with a 538- to 642-nm bandwidth. The detailed information is described in the Supplemental Experimental Procedures.

### Molecular Dynamics Simulations of FG-Nup214^117^ and FG-Nup214^117^⋅CRM1⋅RanGTP Complex

The dynamics of the intrinsically disordered Nup alone and in complex with the CRM1⋅RanGTP complex were sampled using GROMACS 2016, as described in the Supplemental Experimental Procedures.

## Supplemental Information

Document S1. Supplemental Experimental Procedures and Figures S1–S7

Movie S1. Contact Matrix between FG-Nup214^117^ and CRM1, Related to Figure 4 Time-resolved contact matrix between CRM1 and Nup214, as estimated from 10 different replicates performed using AMBER99-sb^∗^-ILDN force field in combination with the TIP4PD water model. Contacts were estimated choosing a cutoff of 1.2 nm and colored as a function of distance between each atom of the two partners, from red (short distances) to white (distances above the cutoff). The movie shows ten different trajectories collated, with the timer at the top of the graph resetting to 0 ns when each run reaches the maximum simulated time (250 ns).

Movie S2. Collated Molecular Dynamics Simulations Trajectories of the FG-Nup214^117^•CRM1•RanGTP Complex, Related to Figure 4 The video shows ten different MD replicates, each 250 ns long with the starting point set at 50 ns for each replicate (the first 50 ns of each run have been considered as equilibration time). To enhance clarity, although being part of the complex, RanGTP is not shown and CRM1 is shown as a gray surface. The backbone of FG-Nup214117 is shown as a yellow ribbon with the investigated FG-repeats shown using the van der Waals radius of atoms composing the repeats.

Document S2. Article plus Supplemental Information
