# Supplementary material for: Two Differential Binding Mechanisms of FG-Nucleoporins and Nuclear Transport Receptors
Source: Cell Rep. 2018 Mar 27;22(13):3660–71. doi: 10.1016/j.celrep.2018.03.022 (PMC5898484; doi:10.1016/j.celrep.2018.03.022)
Supplement: Document S1. Supplemental Experimental Procedures and Figures S1–S7 [file mmc1.pdf]

Cell Reports, Volume *22*

Supplemental Information

Two Differential Binding Mechanisms

of FG-Nucleoporins and Nuclear Transport Receptors

Piau Siong Tan, Iker Valle Aramburu, Davide Mercadante, Swati Tyagi, Aritra Chowdhury, Daniel Spitz, Sarah L. Shammas, Frauke Gräter, and Edward A. Lemke

Figure S1

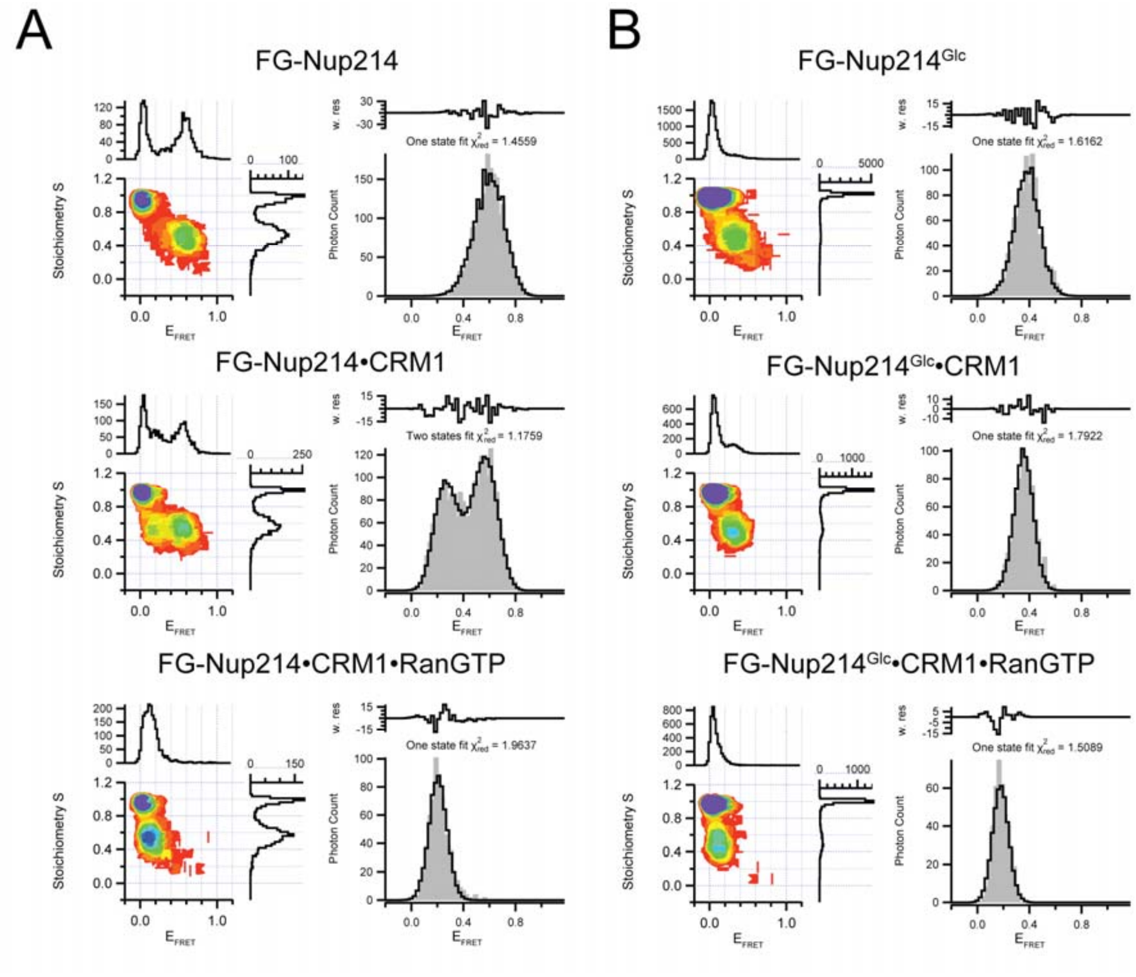

Figure S1. Photon distribution analysis (PDA) histograms, to estimate number of species corresponding to a FRET population, Related to Figure 1 and 3.

Photon distribution analysis (PDA) approach, a method that quantitatively and precisely describes the smFRET distribution, was implemented to analyse data with potentially coexisting multiple species (Kalinin et al., 2010). PDA histograms (expressed as uncorrected E_{FRET}) of (A) FG-Nup214 and (B) FG-Nup214^{Glc} in the presence of CRM1 and CRM1•RanGTP (from top to bottom). PDA confirms that two species are hidden behind the broad population of FG-Nup214•CRM1 interaction and a single species upon adding RanGTP. This suggests that FG-Nup214 undergoes a drastic conformational change upon binding the CRM1•RanGTP and the ensemble collapses into conformations with a rather fixed dye to dye distance in the bound state in agreement with the crystal structure of (Port et al., 2015). Upon interacting with CRM1, FG-Nup214^{Glc} shows a single population, which is similar to its unbound form, in terms of E_{FRET}, while in the additional presence of RanGTP, the same E_{FRET} is reached as for the unglycosylated case. This indicates lower affinity of FG-Nup214^{Glc} to CRM1, but that the overall binding mechanism is conserved.

Figure S2

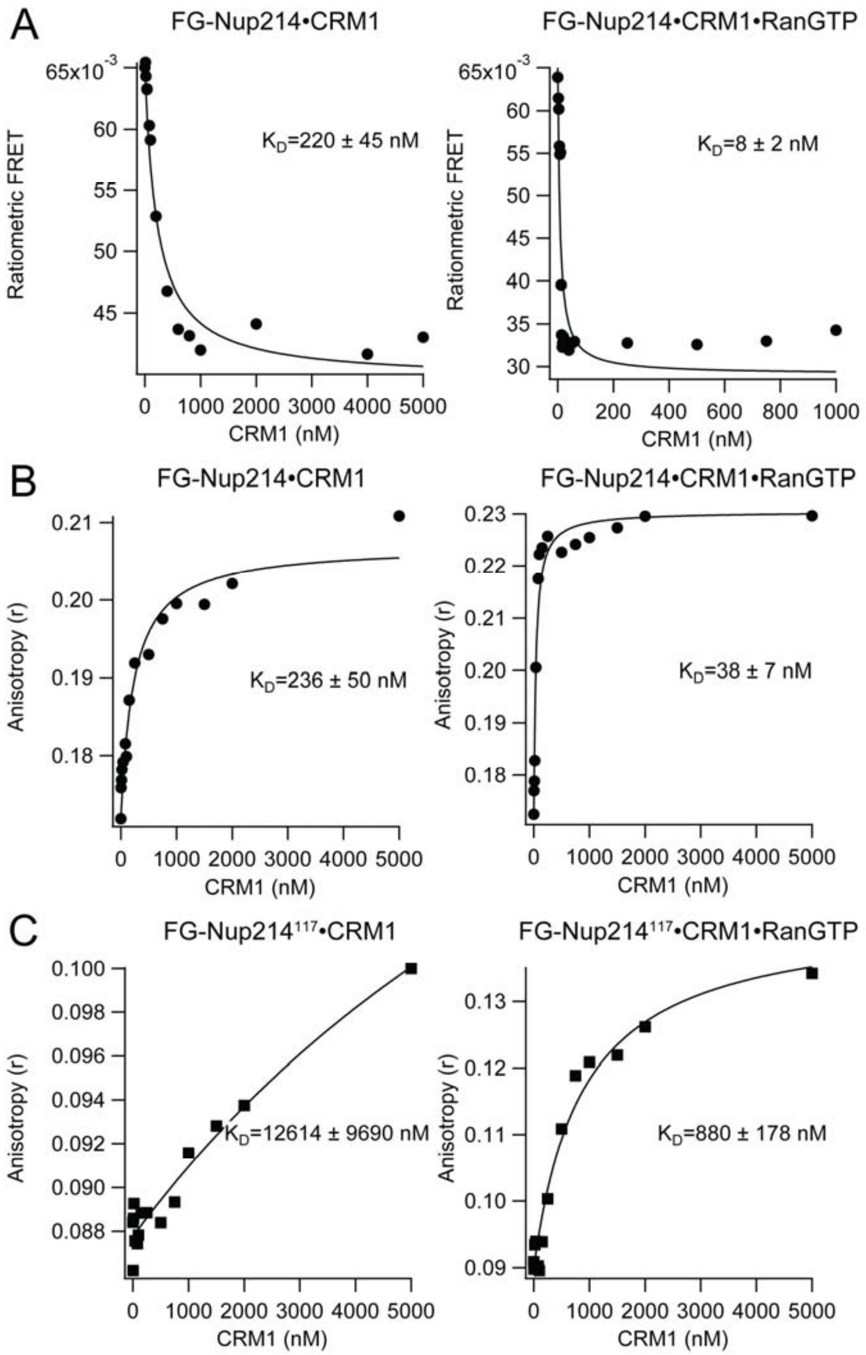

Figure S2. K_D determination of FG-Nup214 with CRM1 and CRM1•RanGTP complex, Related to Figure 1.

(A) FRET titration and (B) anisotropy titration for FG-Nup214. At a concentration of 10 nM FG-Nup214, CRM1 was titrated from 0 nM up to 5000 nM. Both measurements showed that RanGTP (added at a concentration of 1000 nM or same concentration of CRM1, whichever is higher) increases the binding affinity of FG-Nup214 to CRM1. This agrees well with previous findings that CRM1 forms a high affinity, RanGAP-resistant complex with RanGTP and FG-Nup214 (Hutten and Kehlenbach, 2006; Port et al., 2015), (C) Anisotropy titration for FG-Nup214^{117} showed that the affinity of CRM1 on its own for the short fragment is lower so that despite excess of CRM1, the majority of labeled FG-Nup214^{117} can be expected to be unbound in the smFRET experiments.

Figure S3

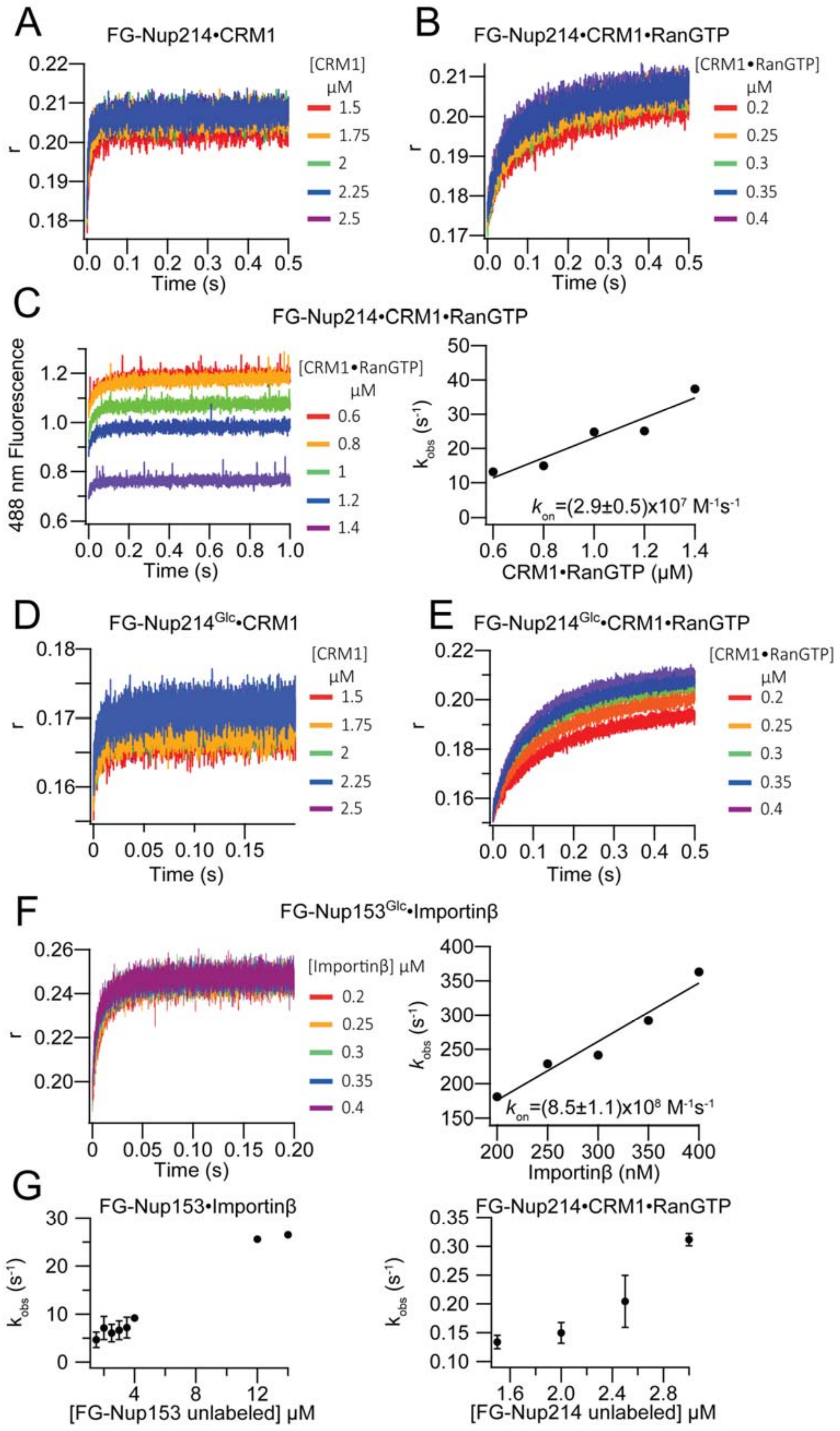

A
FG-Nup214•CRM1
[CRM1] μM
1.5
1.75
2
2.25
2.5
r
Time (s)
B
FG-Nup214•CRM1•RanGTP
[CRM1•RanGTP] μM
0.2
0.25
0.3
0.35
0.4
Time (s)
C
FG-Nup214•CRM1•RanGTP
488 nm Fluorescence
[CRM1•RanGTP] μM
0.6
0.8
1
1.2
1.4
Time (s)
k_{obs} (s^{-1})
k_{on}=(2.9±0.5)x10^{7} $M^{-1}s^{-1}$
CRM1•RanGTP (μM)
D
FG-Nup214^{Glc}•CRM1
[CRM1] μM
1.5
1.75
2
2.25
2.5
Time (s)
E
FG-Nup214^{Glc}•CRM1•RanGTP
[CRM1•RanGTP] μM
0.2
0.25
0.3
0.35
0.4
Time (s)
F
FG-Nup153^{Glc}•Importinβ
[Importinβ] μM
0.2
0.25
0.3
0.35
0.4
Time (s)
k_{on}=(8.5±1.1)x10^{8} $M^{-1}s^{-1}$
Importinβ (nM)
G
FG-Nup153•Importinβ
[FG-Nup153 unlabeled] μM
FG-Nup214•CRM1•RanGTP
[FG-Nup214 unlabeled] μM

Figure S3. Stopped-flow kinetic measurements, Related to Figure 2.

Raw anisotropy traces (r vs time) of 20 nM FG-Nup214 upon mixing with excess of (A) CRM1, (B) CRM1•RanGTP under pseudo-first order conditions. (C) Raw donor fluorescence vs time traces (left) of the double labeled FRET sample upon binding to CRM1•RanGTP, k_{obs} vs [CRM1•RanGTP] plot obtained from fitting the raw traces (right). Raw anisotropy traces (anisotropy vs time) of glycosylated FG-$Nup214^{Glc}$ upon mixing with excess of (D) CRM1, (E) CRM1•RanGTP. (F) Raw anisotropy traces (anisotropy vs time) of FG-$Nup153^{Glc}$ upon mixing with excess of Importinβ. k_{obs} extracted from single exponential fit to these traces are plotted as a function of NTR concentration (right) and the gradient used to obtain the association rate constants (k_{on}). (G) k_{obs} vs [unlabeled FG-Nup] plots, the k_{obs} values were obtained from the stopped-flow dissociation experiments of FG-Nup153•Importinβ (left) and FG-Nup214•CRM1•RanGTP (right) at different concentrations of unlabeled FG-Nup153 and FG-Nup214 respectively. All NTRs were titrated with the range of values as shown in their corresponding colour legend in each experiment. Error bars indicate the standard deviation from the mean of 3 replicates under the same NTR concentration. We note, that for FG-Nup214 for experimental reasons (i.e. aggregation) it was not possible to increase the concentration any higher. For FG-Nup153 at concentrations above 4 μM only single data points are shown (i.e. no error bars), as each point consumed ~24L of expression culture.

Figure S4

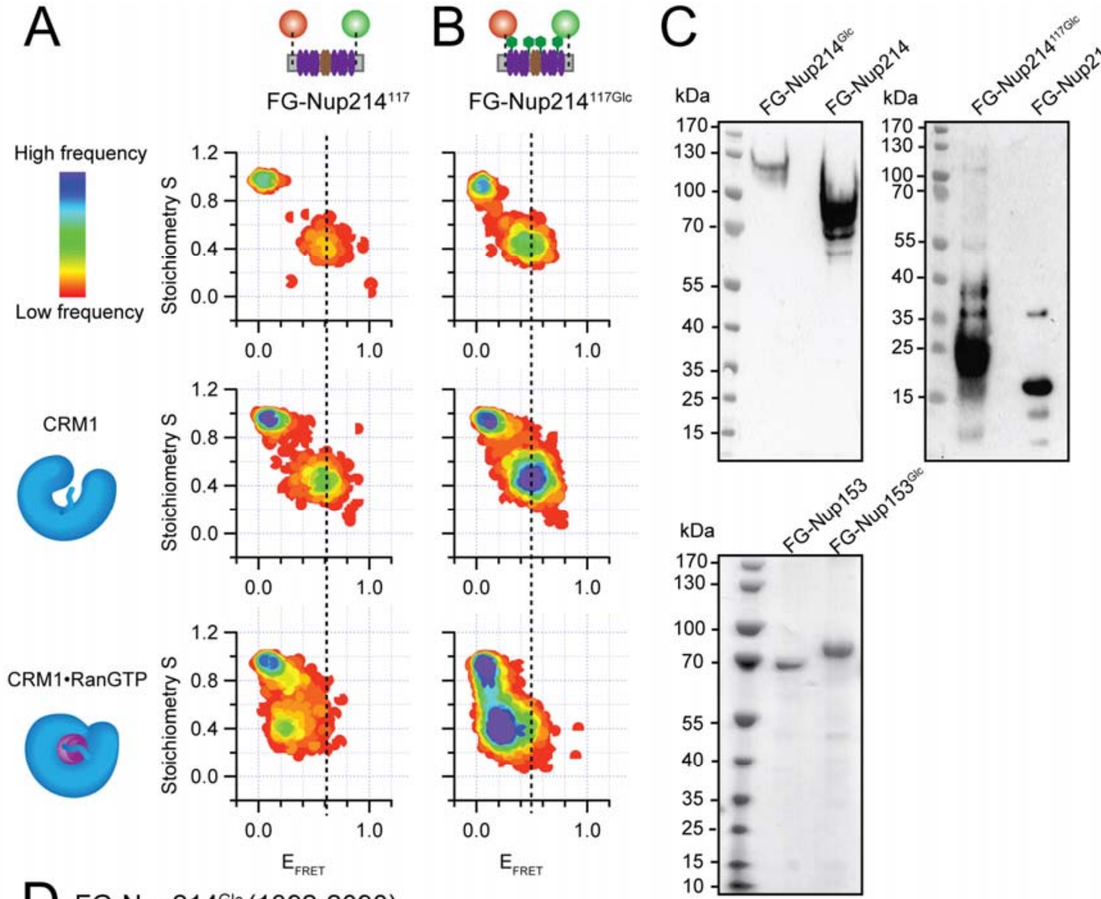

D FG-Nup214^{Glc} (1392-2090)

ATTTSVAPPAATSTSSTAVFGSLPVTSAGSSGVISFGGTSLSAGKTSFSFGSQQTNSTVPPSAPPPTTAATPLPTSFPTLSFGSLLS
SATTPSLPMSAGRSTEEATSSALPEKPGDSEVSASAASLLEEQQSAQLPQAPPQTSDSVKKEPVLAQPAVSNSGTAASSTSLVAL
SAEATPATTGVPDARTEAVPPASSFSVPGQTAVTAAAISSAGPVAVETSSTPIASSTTSIVAPGPSAEAAAFGTVTSGSSVFAQPPA
ASSSSAFNQLTNNTATAPSATPVFGQVAASTAPSLFGQQTGSTASTAAATPQVSSSGFSSPAFGTTAPGVFGQTTFGQASVFGQ
SASSAASVFSFSQPGFSSVPAFGQPASSTPTSTSGSVFGAASSTSSSSSFSFGQSSPNTGGGLFGQSNAPAFGQSPGFGQGG
SVFGGTSAATTTAATSGFSFCQASGFGSSNTGSVFGQAASTGGIVFGQQSSSSSGSVFGSGNTGRGGGFFSGLGGKPSQDAA
NKNPFSSASGGFGSTATSNTSNLFGNSGAKTFGGFASSSFGEQKPTGTFSSGGGSVASQGFGFSSPNKTGGFGAAPVFGSPPT
FGGSPGFGGVPAFGSAPAFTSPLGSTGGKVFGEGTAAASAGGFGFGSSSNTTSFGTLASQNAPTFGSLSQQTSGFGTQSSGF
SGFGSGTGGFSFGSNNSSVQGFGGWRS

E FG-Nup214^{117Glc} (1906-2042)

ASGGFGSTATSNTSNLFGNSGAKTFGGFASSSFGEQKPTGTFSSGGGSVASQGFGFSSPNKTGGFGAAPVFGSP
PTFGGSPGFGGVPAFGSAPAFTSPLGSTGGKVFGEGTAAASAGGFGFGSSSNTTSFGTLASQNA

F FG-Nup153^{Glc} (875-1475)

SAKPGTKSGFKGFDTSSSSSNSAASSSFKFGVSSSSSGPSQTLTSTGNFKFGDQGGFKIGVSSDSGSINPMSEGFKF
SKPIGDFKFGVSSESKPEEVKKDSKNDNFKFGLSSGLSNPVSLTPFQFGVSNLGQEEKKEELPKSSSAGFSFGTGVIN
STPAPANTIVTSENKSSFNLGTIETKSASVAPFTCKTSEAKKEEMPATKGGFSFGNVEPASLPSASVFVLGRTEEKQQE
PVTSTSLVFGKKADNEEPKCQPVFSFGNSEQTKDENSSKSTFSFSMTKPSEKESEQPAKATFAFGAQTSTTADQGAA
KPVFSFLNNSSSSSSTPATSAGGGIFGSSTSSSNPPVATFVFGQSSNPVSSSAFGNTAESSTSQSLLFSQDSKLATTSS
TGTAVTPFVFGPGASSNNTTTSGFGFGATTTSSSAGSSFVFGTGPSAPSASPAFGANQTPTFGQSQGASQPNPPGFG
SISSSTALFPTGSQPAPPTFGTVSSSSQPPVFGQQPSQSAFGSGTTPNSSSAFQFGSSTTNFNFTNNSPSGVFTFGAN
SSTPAASAQPSGSGGFPFNQSPAAFTVGSNGKNVFSSSGTSFSGRKIKTAVRRRK

Figure S4. Conformational change of FG-Nup214^{117} and FG-Nup214^{117Glc} in the presence and absence of CRM1 and CRM1•RanGTP probed by smFRET, Related to Figure 1 and 3.

Analog to Figure 1 and 3, this figure shows the same experiments for the fragment FG-Nup214^{117}. (A) unglycosylated and (B) glycosylated in the absence and presence of 1000 nM CRM1 and CRM1•RanGTP (at 1000 nM for each protein). The dotted line visualizes the shift of the E_{FRET} peak. FG-Nup214^{117Glc} does not show a conformational change upon interacting to CRM1. However, upon addition of CRM1•RanGTP there is a shift in the double labeled population (S= 0.5) showing an E_{FRET} value (= 0.2). A similar E_{FRET} change takes place in the unglycosylated and glycosylated form. This indicates overall conservation of the binding mechanism independent of the glycosylation status. (C) Western blot analysis of FG-Nup214 probed with mAB414 and SDS-PAGE of FG-Nup153. (D-F) The results of a LC-MS/MS analysis underlining the high amount of putative O-glycosylated sites. Protein sequence of (D) FG-Nup214^{Glc}, (E) FG-Nup214^{117Glc} and (F) FG-Nup153^{Glc} showing both FG-Nup214^{Glc} and FG-Nup153^{Glc} were heavily glycosylated and several potential glycosylation sites were identified. Proteins were digested using chymotrypsin in order to receive best coverage of the protein regions of interest. The respective proteins could be identified with a high confidence. The sequences for which peptide evidence were detected are underlined. Further, during the database search using Mascot, O-glycosylation of serine and threonine were selected and putative modified sites are indicated in red color. In order to receive a higher confidence in the predicted sites of modification the Mascot ion score was raised to 32.

Figure S5

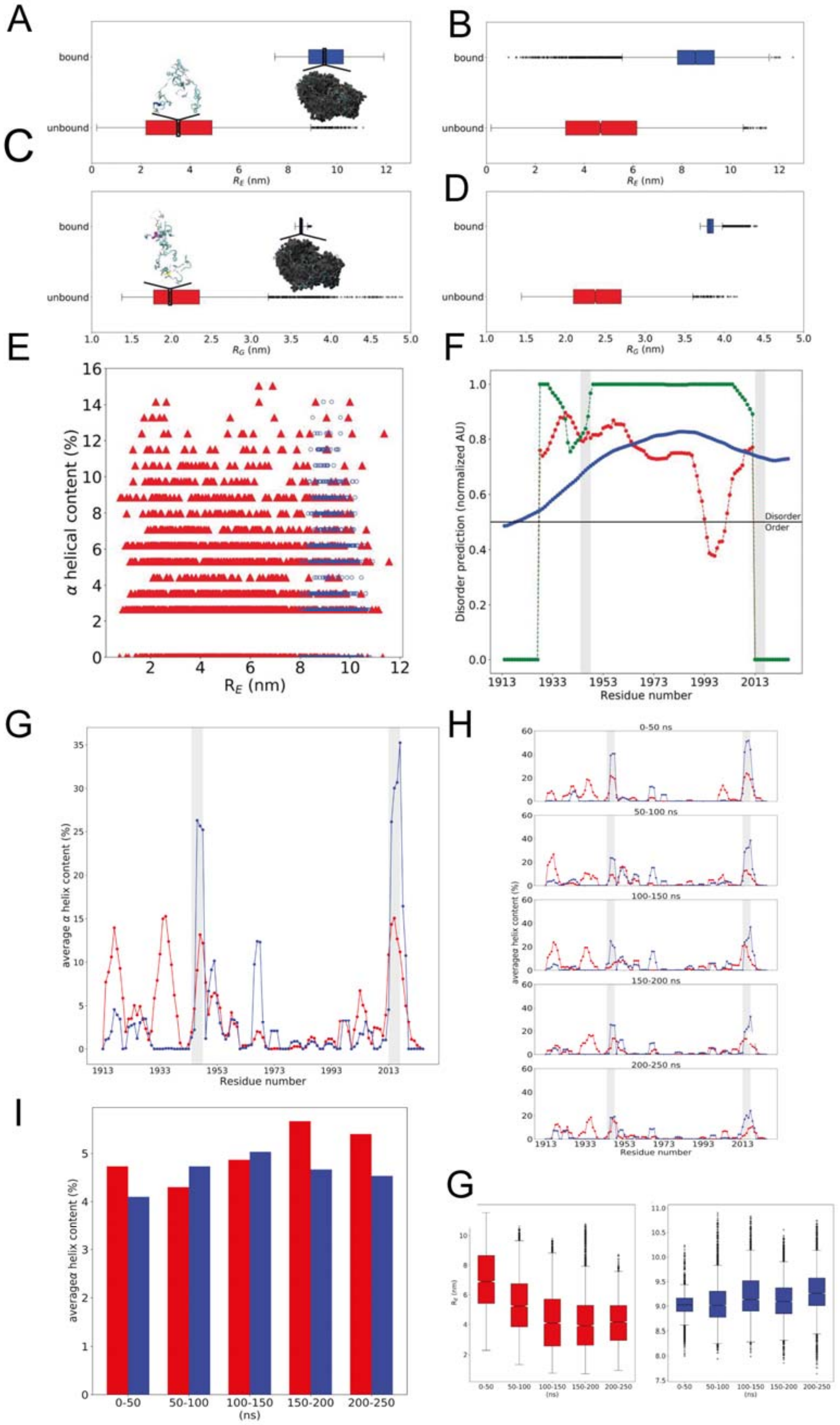
A
B
C
D
E
F
G
H
I
G
bound
unbound
R_E (nm)
R_G (nm)
α helical content (%)
Disorder prediction (normalized AU)
Disorder
Order
Residue number
average α helix content (%)
0-50 ns
50-100 ns
100-150 ns
150-200 ns
200-250 ns
0-50
50-100
100-150
150-200
200-250
(ns)

Figure S5. Analysis of FG-Nup214[117] dimensions in its bound and unbound state and computationally sampled and predicted secondary structure content and overall dimensions of FG-Nup214[117], Related to Figure 4.

Box and whisker plots showing the computed end-to-end (R_E) distance (A-B) and radius of gyration (R_G) (C-D) distributions for FG-Nup214[117] in the unbound (red) and bound states (blue) sampled with AMBER99-sb*-ILDN-TIP4PD (A and C) and KBFF-SPC/E (B and D) force fields and water models. The distributions were calculated considering the last 150 ns of a total of 250 ns production runs. Each box indicates the range (interquartile range; IQR) between the lower (Q1) and upper (Q3) quartile whereas the solid line represents the distribution's median. The whiskers report values that fall below Q1 – 1.5*IQR or above Q3 + 1.5*IQR. Outliers are shown as crosses below or above whiskers. (E) α-helical content of the FG-Nup214 protein as a function of the R_E for the unbound (red) and bound states (blue). (F) Sequence-based prediction of disorder-propensity for the simulated FG-Nup214 peptide using the XL1-XT (red (Romero, 1997)), VL3-BA (blue (Kehlenbach et al., 1999)) and CaN-XT (green (Garner, 1999)) algorithms available in PONDR (http://www.pondr.com/). The arbitrary threshold distinguishing ordered and disordered sequences is reported at 0.5 intrinsic disorder prediction score and shown as a horizontal black line. (G) Secondary structure analysis of the amount of α-helical conformation for each residue as found along the simulated trajectories of FG-Nup214[117] in isolation (red) and in complex to CRM1•RanGTP (blue). (H) Percentage of α-helical content along the FG-Nup214[117] sequence is reported for five different 50 ns-long time windows for the unbound (red) and bound (blue) state, collected for each of the 10 replicates. (I) Average α-helical content, shown in percentage per time window, for the FG-Nup214[117] bound and unbound state. (J) R_E distributions as a function of five different 50 ns-long time windows collected for each of the 10 replicates for the unbound (red) and bound (blue) state of FG-Nup214[117].

Figure S6

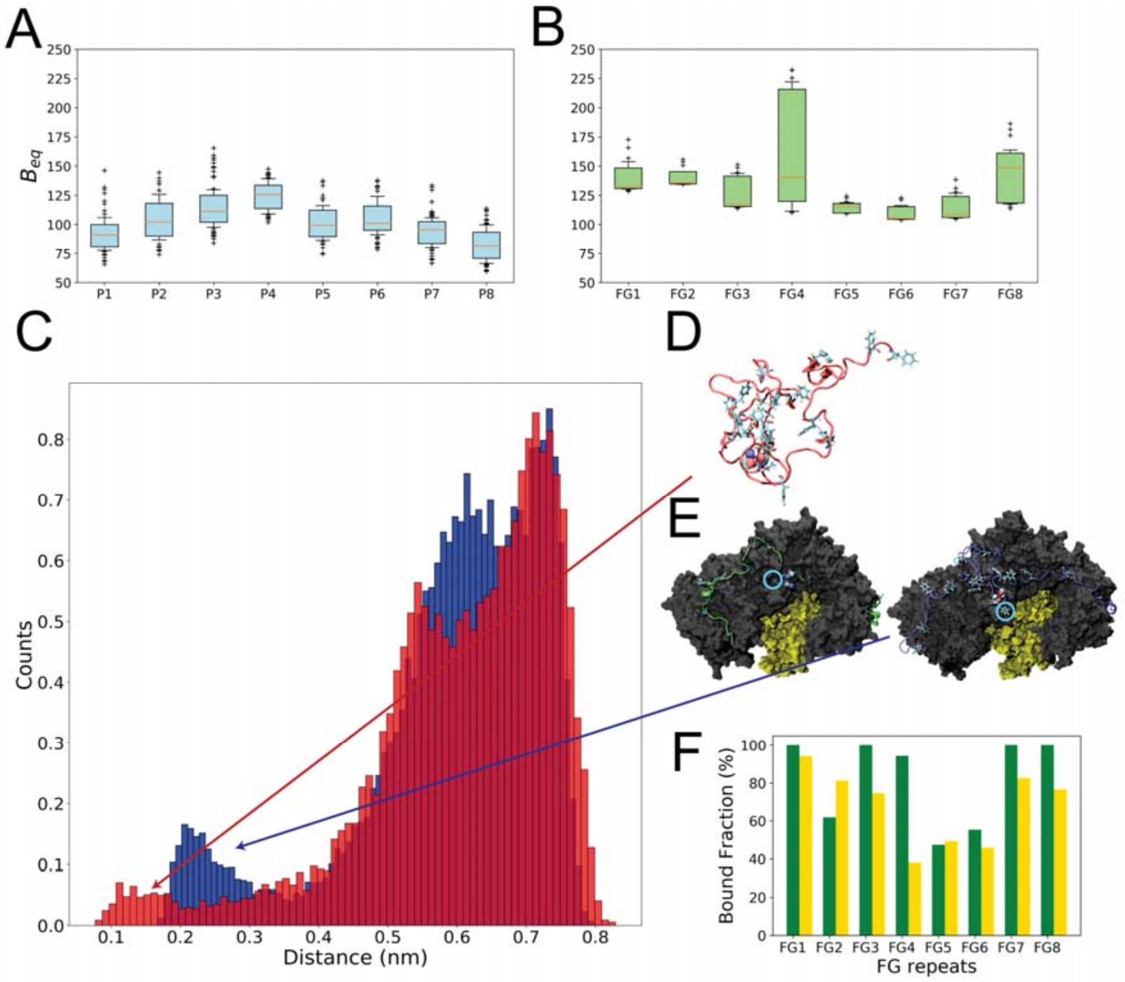

Figure S6. Equal B-factor (B_{eq}) of CRM1 FG-binding pockets/ co-crystallized FG-Nup214[117] FG repeats and distance distributions of the T1981-G1984 backbone hydrogen bond and fraction of FG-Nup214[117] FG repeats bound to CRM1, Related to Figure 4.

(A) B_{eq} distributions for the residues composing the CRM1 binding pockets allocating the eight FG repeats (FG1-FG8) as observed by (Port et al., 2015) and for which the B_{eq} distributions are reported in (B). Each box indicates the range (interquartile range; IQR) between the lower (Q1) and upper (Q3) quartile whereas the solid line represents the distribution's median. The whiskers report values that fall below Q1 – 1.5*IQR or above Q3 + 1.5*IQR. Outliers are shown as crosses below or above whiskers. Distributions have been computed considering the B_{eq} obtained for each atom of the residues, which are described as a part of the analysed pocket or FG repeats. The red lines show the median of each distribution. (C) Distribution of distances between the T1981@O---G1984@H hydrogen bond suggested to be functional to the binding of the FG5 (F1982-G1983) to CRM1. The distribution of distances sampled during MD simulations is reported for both the unbound (red) and bound states (blue) of FG-Nup214[117]. We note that the experimental B-factors for FG repeats follow neither the trend from simulations nor the trend of the B-factors of CRM1 pocket residues, which might be attributed to the challenge in the X-ray refinement of broad conformational ensembles (Kuzmanic et al., 2014). (D) A representative conformation recording the lowest distance for the analysed bond; in the majority of cases such a low distance is found in globule-like conformations, which are far from the bound-prone semi-extended pose observed in the complex. (E) Conformations of the FG-Nup214[117]•CRM1•RanGTP complex co-crystallized by Port *et al.* (left panel) and corresponding (right) to the lower end of the h-bond distance distribution sampled in the bound state (blue) (Port et al., 2015). F1982 is highlighted by a cyan circle. (F) The fraction of FG-Nup214[117] FG repeats bound to CRM1 is shown as observed from the simulations performed using the

AMBER99-sb*-ILDN (green bars) or the KB (yellow bars) force fields. The bound fraction of each FG repeat was calculated considering the number of contacts between a repeat and any atom of CRM1 within a radius of 0.4 nm. Frames in which the number of contacts were different from 0 were considered as ‘bound’, whereas frames in which no contacts were observed were considered as ‘unbound’. The percentage of bound fraction has then been retrieved by normalising the fraction of frames reporting binding over the total number of simulated frames.

Figure S7

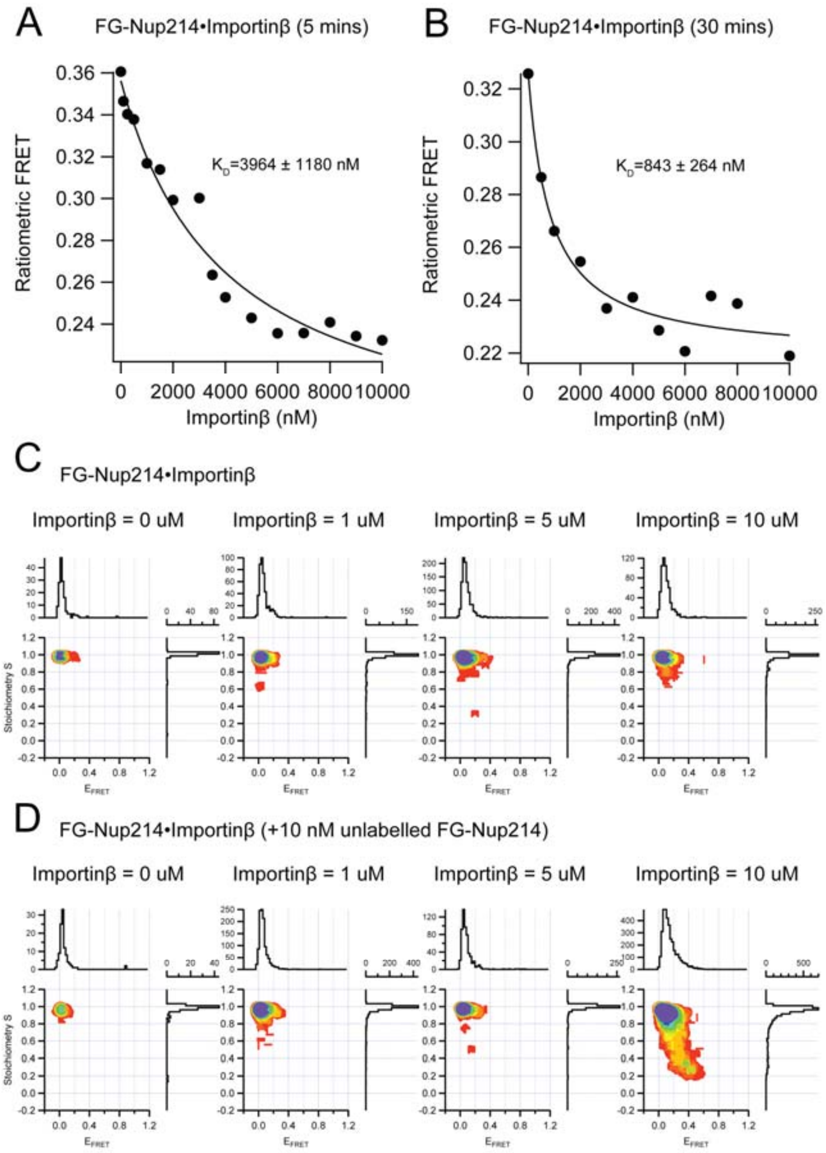

Figure S7. Time and concentration dependence of FG-Nup214•Importinβ, Related to Figure 5.

Ensemble FRET measurements to study incubation time dependent K_D of FG-Nup214•Importinβ interactions. We observed different K_D value for different titrations of Importinβ (ranging from 0 to 10000 nM) interacting with 10 nM FG-Nup214 performed after different incubations times of (A) 5 minutes and (B) 30 minutes. Next, we separately labeled FG-Nup214 with Alexa488 and Alexa594 and created 1:1 mixture at a total protein concentration of 50 pM. SmFRET measurements to detect coincide of the two dyes (i.e. population of S>0) were performed with 10 nM unlabeled FG-Nup214 (D) and without (C). Under both conditions Importinβ was titrated in ranging from 0 to 10 µM. Plots show clearly an appearance of a population with FRET and S higher than 0 in the present of high concentration of Importinβ ~(10 µM) when interacting with nM concentration of FG-Nup214. This clearly indicates aggregation, in which donor and acceptor dye (tentatively from different molecules) come even closer than 10 nm together and can undergo FRET. We learn from this that under smFRET conditions used in Figure 1 and 5, FRET is not originating from aggregation. However, FG-Nup214 and Importinβ has also a more complex phase diagram, so that at other time points and or higher concentrations, also aggregation phenomena can occur and lead to FRET, in line with previous observations for other Nup and NTR combinations (Kehlenbach et al., 1999; Milles et al., 2013).

Supplemental Experimental Procedures

Protein Expression, Purification and Labeling

FG-Nup214/ FG-Nup214^{117}: The codon-optimized (Mr. Gene, Regensburg, Germany) FG-Nup214 disordered region (amino acids 1392 to 2090 and 1906 to 2042 for FG-Nup214^{117}, numbering with respect to the full length protein Uniprot: P35658 protein) was recombinantly expressed in *Escherichia coli* (*E.coli*) strain BL21(AI) (Invitrogen, Carlsbad,CA). For 117 aa long FG-Nup214 fragment, the final purified sequence was 137 aa (1906 to 2042) to incorporate the FRET dye pair. Plasmids from the labeling mutants were cotransformed with pEvol-AcF, which encodes for the noncanonical amino acid p-acetylphenylalanine (AcF) Amber suppressor tRNA/tRNAsynthetase pair (Lemke, 2011). Cultures were grown in Terrific Broth (TB) medium at 37ºC in the presence of 1 mM AcF. Expression was induced at OD_{600}= 1 with 0.02% arabinose and 1 mM IPTG. The detailed protocol for FG-Nup214 purification was as previously described for FG-Nup153 in (Milles and Lemke, 2011).

CRM1: CRM1 was recombinantely expressed in *E.coli* BL21 (AI) from a pQE60-CRM1-intein-12His vector. Cultures were grown in TB medium at 37ºC and induced with 1 mM IPTG. After induction, temperature was reduced to 18ºC and incubated for 18 hours. *E.coli* cells were harvested and purified under native conditions according to standard purification protocols for His-tagged proteins.

RanQ69L (1-180): RanQ69L protein was expressed and purified in the same way as CRM1. His-tag was cleaved with by a TEV protease. GTP loading of RanQ69L was performed by incubating the purified protein with the non-hydrolyzable GTP (γ-S-GTP) nucleotide in a ratio 1:20 (protein: nucleotide) in presence of 10 mM EDTA, 1 µl of alkaline phosphatise per 10 nmoles protein. The reaction was incubated at 20^{0}C for 2 hours. The sample was centrifuged (13,000 rpm, 15 minutes) to remove aggregates. Then the supernatant was supplemented with $MgCl_2$ to a final concentration of 15 mM to stabilize the nucleotide bound to RanQ69L protein.

OGT: O-Linked N-Acetylglucosamine (GlcNAc) Transferase tagged with a 12His was cloned into a pTXB3 vector and expressed in BL21 (AI cells). Cells were induced at OD_{600}: 0.6-0.8 and OGT were expressed at 18ºC overnight under shacking conditions. Cells were pelleted, resuspended and lysed on lysis buffer (50 mM Tris, pH 7.4, 150 mM NaCl, 5 mM imidazole and the buffer was supplemented with 0.2 mM TCEP and 1 mM PMSF) After centrifugation for 1 h at 18.000 rpm the supernatant was incubated with Ni-beads. After extensive washes with 10-20 mM imidazole the protein was eluted with 400 mM imidazole. Eluted OGT1 was then concentrated and run over size exclusion chromatography (adapted protocol from (Labokha et al., 2013)). The selected fractions where then concentrated and buffer exchanged with 50 mMTris, pH 7.4, 150 mM NaCl, 0.2 mM TCEP ,1 mM PMSF and 20 % glycerol. The samples where flash frozen and kept at -80ºC.

Labeling of FG-Nup214/ FG-Nup214^{117} were performed by using standard protocol to site-specifically introduce Alexa488 as a donor and Alexa594 as an acceptor as described in (Milles and Lemke, 2011). Single cysteines and Amber stop codons (TAG) were introduced into the FG-Nup214 and sequence by site-directed mutagenesis. The purified labeling mutants were site-specifically labeled with Alexa594-maleimide as acceptor and the AcF was labeled with Alexa488-hydroxylamine. The labeling positions of FG-Nup214 were determined based on the architecture of the CRM1•SPN1•RanGTP•FG-Nup214 complex (PDB: 5DIS). Labeling of FG-Nup214 for stopped-flow measurements was performed with Cy3B-maleimide.

In vitro glycosylation of FG-Nup

FG-Nup214 and FG-Nup153 were glycosylated with UDP-GlcNAc (Sigma: U4375), which were attached to serine and threonine residues and the process was catalysed by the O-GlcNAc transferase (OGT1). The *in vitro* glycosylation was performed following the protocol described by (Labokha et al., 2013). FG-Nup from cell lysate where washed with washing buffer (as described in the Nucleoporin purification section mentioned above), then washed with glycosylation buffer (50 mM Tris, pH 7.5, 200 mM NaCl, 20 mM $MgCl_2$, 0.2 mM TCEP and 1 % Tween20). The beads where then resuspended in 20 bead volumes of glycosylation buffer containing 5 µM OGT and 1 mM UDP-GlcNAc. The beads where rotated at room temperature for 16 h. The beads where washed with glycosylation buffer and subsequently with the washing buffer used in the FG-Nup purification protocol.

Molecular modelling and molecular dynamics simulations of the FG-Nup214[117] and FG-Nup214[117]•CRM1•RanGTP complexes

Simulations were started after modelling parts of the bound partners that couldn't be resolved by X-ray crystallography. The lack of any known conformation of FG-Nup214[117] made the modelling of the protein starting from homology to a template impossible. Therefore, the missing segments of FG-Nup214 were modelled in a random conformation subsequently equilibrated during the first 150 ns of the molecular dynamics run. The nucleoporin were modelled by adding each residues in the most populated rotameric population according to the Dunbrack library (Dunbrack, 2002), making sure that any atom of the added conformers would not clash with any resolved atoms of the X-ray crystal structure. The complexes featuring the modelled Nup214[117] were then prepared for simulations as described below. Firstly, hydrogen atoms were added and molecular topologies were built adopting the parameters defined by the AMBER99-sb*-ILDN force field (Lindorff-Larsen et al., 2010). Molecules were then placed into a dodecahedron box filled with TIP4PD water molecules (Piana et al., 2015). The TIP4PD water model has been explicitly parameterized to avoid the artificial over-collapse of intrinsically disordered proteins but, at the same time, it has shown to avoid an excessive destabilization of structured proteins such as, in this case, CRM1 and RanGTP. Additionally, we performed simulations also employing the Kirkwood-Buff force field (Ploetz et al., 2010) and compared the results obtained for the FG-Nup214[117] ensembles. Importantly, the combination of AMBER99-sb*-ILDN with TIP4PD has been shown to yield realistic dimensions of IDPs and to destabilise structured proteins by only ~8.4 kJ mol^{-1} (Piana et al., 2015). Na^+ and Cl^- Ions were added to the solutions by replacing water molecules in the box to finally achieve systems' neutrality and a total ionic concentration of 0.1 M. The systems were then energy minimised using a steepest descent algorithm with a step size of 0.001 nm, stopped when the force was lower than 1 kJ mol^{-1}. Subsequently, solvent equilibration around the molecules was achieved in two steps. Firstly, a 0.5 ns-long run was performed at a constant number of particles, volume and temperature (NVT). In this step, temperature was coupled to a 300 K using a V-rescale thermostat (Bussi et al., 2007) and starting velocities were randomly generated for each particle following a Brownian distribution at 300 K. Secondly, a 0.5 ns-long NpT step, was carried out by coupling both pressure and temperature at 1 atm and 300 K respectively, using a Parrinello-Rahman barostat (Parrinello and Rahman, 1981) and a V-rescale thermostat. Pressure and temperature were coupled every 2.0 and 0.1 ps respectively. In both NVT and NpT steps, proteins were restrained in the three dimensional space by applying on each particle a potential of 1000 kJ mol^{-1}. Subsequently to equilibration, production runs were performed on 10 different replicates, differing for the initial random velocities assigned in the NVT equilibration step and each step was run for 125 million steps using a time step of 2 fs, finally yielding a production run time of 250 ns for each replicate and the first 150 ns were discarded for analysis as considered equilibration time. Interactions between the particles were computed using the Verlet scheme (Páll and Hess, 2013) for neighbouring search and a cutoff for Lennard-Jones and Coulomb interactions of 1 nm. The analysis of the trajectories was achieved using tools available within the GROMACS suite (Van Der Spoel et al., 2005) and routines implemented in the analysis suite called MDAnalysis (Michaud-Agrawal et al., 2011). Visualization and rendering of the structures was performed using visual molecular dynamics (VMD) version 1.9.3 (Humphrey et al., 1996). Data plotting and visualisation was finally performed using matplotlib (Hunter, 2007). Disorder predictions were performed using PONDR web server (http://pondr.com) and employing three main algorithms available in it: VL3-BA (Milles et al., 2013), CAN_XT (Garner, 1999) and XL1-XT (Romero, 1997).

SmFRET experiment and data analysis

The smFRET experiments were performed using a confocal-based microscope with a 1.27 numerical aperture (NA) and a 60x water immersion objective (Nikon). The fluorescence emission signal was split into donor and acceptor channels, with both parallel and perpendicular polarization orientation. The fluorescence signal was then detected on photon counting detectors (APD and hybrid PMA, Picoquant, Berlin) and directed to counting electronics (HydraHarp400, Picoquant, Berlin). The fluorescence intensities were used to calculate the FRET efficiencies (E_{FRET}) by the corrected FRET equation: $E_{FRET} = I_A/(I_A + \gamma I_D)$, where γ is the correction factor which accounts for the differences in quantum yield and detection efficiency between the donor and the acceptor, I_A and I_D are the acceptor and donor intensities, respectively. The data analysis was performed by a custom written Igor-Program (Fuertes et al., 2017).

The 2D S vs E_{FRET} plots obtained from pulse interleaved excitation (PIE) (Muller et al., 2005) show the populations according to the stoichiometry of the dyes (y axis) on the disordered protein being S= 1 for the molecules labeled with donor only dye and S= 0.5 for the molecules containing a donor and an acceptor dye

with 1:1 ratio and S= 0 for the molecule labeled with acceptor dye only. Donor or acceptor only population can arise from dye photophysics and/or incomplete labeling. The population at S=0.5 is thus the one in which we monitored possible changes in the E_{FRET} values (x axis). E_{FRET} value shifting towards 0 indicates an increase of the distance between the dyes, leading to a decrease of the efficiency of energy transferred from the donor to the acceptor dye. All the smFRET experiments in this paper were performed at 50 pM FG-Nup and 1 μM of excess NTR. Concentration ranges are given in the respective data sets. Excess of the NTR does not necessarily indicate that binding could be saturated. In particular in smFRET, due to the stringent signal to noise requirement, to high NTR concentration can lead to large background that would compromise signal quality too much.

Fluorescence stopped-flow measurement

Association kinetics were monitored by following the fluorescence anisotropy change of 20 nM FG-Nup214 labeled with Cy3B (at the position 1905 in relation to the full length) upon mixing with different concentrations, ranging from 0.2-2.4 μM, of CRM1 and RanGTP under pseudo-first order conditions. Due to the higher affinity, kinetic experiments were performed only for full length FG-Nup214 domain in all experiments. Traces were fit to a single exponential function and the concentration dependent observed rates (k_{obs}) were then plotted against their corresponding NTR concentration. From the slope of the linear fit the association rate constant (k_{on}) was obtained. Analogous to FG-Nup214, 20 nM of FG-Nup153 labeled at position 1391C with Cy3B was used for the association and dissociation measurements of FG-Nup153 and Importinβ. The dissociation experiments were performed with 20 nM FG-Nup and 0.5 μM CRM1/RanGTP or 1 μM Importinβ. The preformed complexes were then rapidly mixed with 1.5 to 14 μM unlabeled FG-Nup (see below). The estimated dissociation rate constants were obtained by fitting an exponential decay to the observed anisotropy traces.

Dissociation rate constant (k_{off}) determination in multivalent systems

The kinetic dissociation measurements show a k_{off} at least one order of magnitude difference between FG-Nup153•Importinβ and FG-Nup214•CRM1•RanGTP complexes. While we do acknowledge that some differences can also occur from different effects of multivalency and the intrinsic property of segmental motion of IDPs in anisotropy measurements (Milles and Lemke, 2014), FG-Nup153 has 60 Fs and FG-Nup214 has 62 Fs and the recorded complex half-life is at least 60 fold higher for the CRM1•RanGTP case (Figure 2B). Thus another major biophysical parameter (k_{off}) is different for the two FG-Nup•NTR complexes.

Our dissociation kinetic experiments of multivalent proteins like FG-Nup153 and FG-Nup214 report on the $k_{off,global}$ value which corresponds to when ~all of the binding motifs of the disordered nucleoporin are unbound from the multivalent NTR, and not on $k_{off,\ individual.}$ The relationship between $k_{off,\ individual}$ and $k_{off,global}$ depends on multivalency, linker length and effective concentration. In a multivalent system such as ours, the $k_{off,global}$ value obtained from a kinetic dissociation experiment will thus be orders of magnitude lower than those for each motif ($k_{off,\ individual}$) (Kramer and Karpen, 1998). Taking multivalency into account is of paramount importance if one aims to understand NTR transport and interactions with FG-Nups.

In addition, due to the reversible binding of FG-Nup•NTR complexes, we performed the dissociation kinetic experiments under different concentrations of unlabeled FG-Nup (Figure S3G). The obtained k_{obs} increased with increasing concentrations of unlabeled FG-Nup. Due to experimental constraints we were not able to reach the conditions at which the k_{off} of both systems reached a plateau since at very high concentrations of unlabeled protein, these long disordered proteins tend to aggregate. For this reason it is possible that the differences in the complex half-life are larger than the reported 60 fold obtained at 2 μM (100x excess) of unlabeled FG-Nup.

Supplemental references

Bussi, G., Donadio, D., and Parrinello, M. (2007). Canonical sampling through velocity rescaling. J Chem Phys *126*, 014101.

Dunbrack, R.L. (2002). Rotamer libraries in the 21(st) century. Curr Opin Struc Biol *12*, 431-440.

Fuertes, G., Banterlea, N., Ruff, K.M., Chowdhury, A., Mercadante, D., Koehler, C., Kachala, M., Girona, G.E., Milles, S., Mishra, A., *et al.* (2017). Decoupling of size and shape fluctuations in heteropolymeric sequences reconciles discrepancies in SAXS vs. FRET measurements. Proceedings of the National Academy of Sciences of the United States of America *114*, E6342-E6351.

Garner, E., Romero, P., Dunker, A.K., Brown, C. & Obradovic, Z (1999). Predicting Binding Regions within Disordered Proteins. Genome Inform Ser Workshop Genome Inform *10*, 41-50.

Humphrey, W., Dalke, A., and Schulten, K. (1996). VMD: visual molecular dynamics. J Mol Graph *14*, 33-38, 27-38.

Hunter, J.D. (2007). Matplotlib: A 2D graphics environment. Computing In Science & Engineering *9*, 90-95.

Hutten, S., and Kehlenbach, R.H. (2006). Nup214 is required for CRM1-dependent nuclear protein export in vivo. Molecular and Cellular Biology *26*, 6772-6785.

Kalinin, S., Valeri, A., Antonik, M., Felekyan, S., and Seidel, C.A.M. (2010). Detection of Structural Dynamics by FRET: A Photon Distribution and Fluorescence Lifetime Analysis of Systems with Multiple States. Journal of Physical Chemistry B *114*, 7983-7995.

Kehlenbach, R.H., Dickmanns, A., Kehlenbach, A., Guan, T.L., and Gerace, L. (1999). A role for RanBP1 in the release of CRM1 from the nuclear pore complex in a terminal step of nuclear export. Journal of Cell Biology *145*, 645-657.

Kramer, R.H., and Karpen, J.W. (1998). Spanning binding sites on allosteric proteins with polymer-linked ligand dimers. Nature *395*, 710-713.

Kuzmanic, A., Pannu, N.S., and Zagrovic, B. (2014). X-ray refinement significantly underestimates the level of microscopic heterogeneity in biomolecular crystals. Nature Communications *5*, 3220.

Labokha, A.A., Gradmann, S., Frey, S., Hulsmann, B.B., Urlaub, H., Baldus, M., and Gorlich, D. (2013). Systematic analysis of barrier-forming FG hydrogels from Xenopus nuclear pore complexes. Embo Journal *32*, 204-218.

Lemke, E.A. (2011). Site-specific labeling of proteins for single-molecule FRET measurements using genetically encoded ketone functionalities. Methods in molecular biology *751*, 3-15.

Lindorff-Larsen, K., Piana, S., Palmo, K., Maragakis, P., Klepeis, J.L., Dror, R.O., and Shaw, D.E. (2010). Improved side-chain torsion potentials for the Amber ff99SB protein force field. Proteins *78*, 1950-1958.

Michaud-Agrawal, N., Denning, E.J., Woolf, T.B., and Beckstein, O. (2011). MDAnalysis: a toolkit for the analysis of molecular dynamics simulations. J Comput Chem *32*, 2319-2327.

Milles, S., Huy Bui, K., Koehler, C., Eltsov, M., Beck, M., and Lemke, E.A. (2013). Facilitated aggregation of FG nucleoporins under molecular crowding conditions. EMBO reports *14*, 178-183.

Milles, S., and Lemke, E.A. (2011). Single Molecule Study of the Intrinsically Disordered FG-Repeat Nucleoporin 153. Biophysical Journal *101*, 1710-1719.

Milles, S., and Lemke, E.A. (2014). Mapping multivalency and differential affinities within large intrinsically disordered protein complexes with segmental motion analysis. Angew Chem Int Ed Engl *53*, 7364-7367.

Muller, B.K., Zaychikov, E., Brauchle, C., and Lamb, D.C. (2005). Pulsed interleaved excitation. Biophysical Journal *89*, 3508-3522.

Páll, S., and Hess, B. (2013). A flexible algorithm for calculating pair interactions on SIMD architectures. Computer Physics Communications *184*, 2641-2650.

Parrinello, M., and Rahman, A. (1981). Polymorphic transitions in single crystals: A new molecular dynamics method. J Appl Phys *52*, 7182-7190.

Piana, S., Donchev, A.G., Robustelli, P., and Shaw, D.E. (2015). Water dispersion interactions strongly influence simulated structural properties of disordered protein States. The journal of physical chemistry B *119*, 5113-5123.

Ploetz, E.A., Bentenitis, N., and Smith, P.E. (2010). Developing Force Fields from the Microscopic Structure of Solutions. Fluid phase equilibria *290*, 43.

Port, S.A., Monecke, T., Dickmanns, A., Spillner, C., Hofele, R., Urlaub, H., Ficner, R., and Kehlenbach, R.H. (2015). Structural and Functional Characterization of CRM1-Nup214 Interactions Reveals Multiple FG-Binding Sites Involved in Nuclear Export. Cell Rep *13*, 690-702.

Romero, O.D., K (1997). Sequence Data Analysis for Long Disordered Regions Prediction in the Calcineurin Family. Genome Inform Ser Workshop Genome Inform *8*, 110-124.

Van Der Spoel, D., Lindahl, E., Hess, B., Groenhof, G., Mark, A.E., and Berendsen, H.J. (2005). GROMACS: fast, flexible, and free. J Comput Chem *26*, 1701-1718.
