# Supplementary material for: Two Differential Binding Mechanisms of FG-Nucleoporins and Nuclear Transport Receptors
Source: Cell Rep. 2018 Mar 27;22(13):3660–71. doi: 10.1016/j.celrep.2018.03.022 (PMC5898484; doi:10.1016/j.celrep.2018.03.022)
Supplement: Document S2. Article plus Supplemental Information [file mmc4.pdf]

Cell Reports

Article

Two Differential Binding Mechanisms of FG-Nucleoporins and Nuclear Transport Receptors

Graphical Abstract

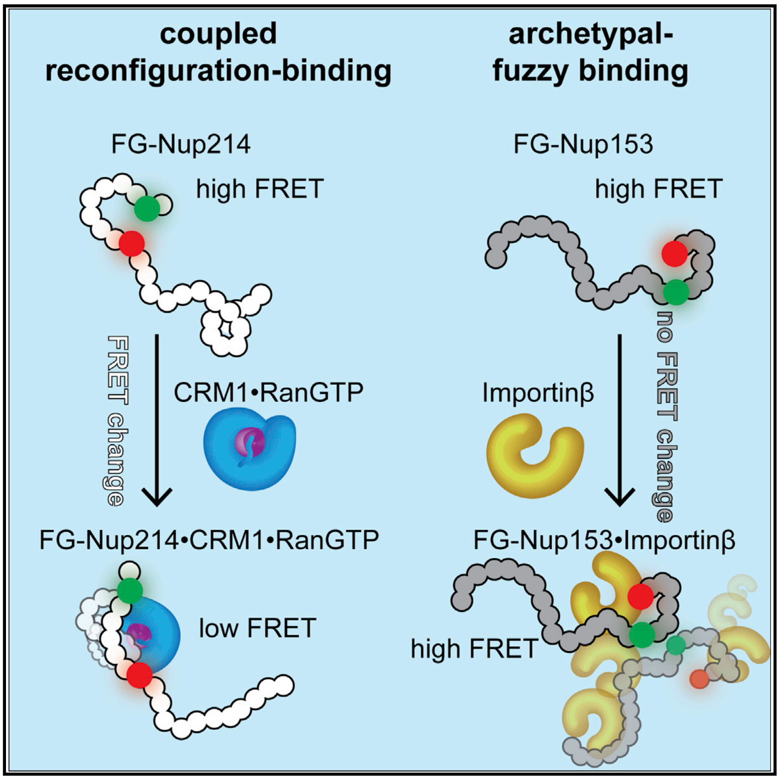

Authors

Piau Siong Tan, Iker Valle Aramburu, Davide Mercadante, ..., Sarah L. Shammas, Frauke Gräter, Edward A. Lemke

Correspondence

frauke.graeter@h-its.org (F.G.), edlemke@uni-mainz.de (E.A.L.)

In Brief

Archetypal-fuzzy complexes found in most FG-Nucleoporin•nuclear transport receptor complexes allow fast yet specific nuclear transport. Tan et al. show that FG-Nup214, located at the periphery of the nuclear pore complex, binds to CRM1•RanGTP via a coupled reconfiguration-binding mechanism, which can enable different functionalities e.g., cargo release.

Highlights

- Identification of two differential binding mechanisms in the nuclear transport pathway
- FG-Nup214 does not bind CRM1 via an archetypal-fuzzy complex
- Glycosylated FG-Nups maintain their NTR-binding mechanisms
- Linker regions of FG-Nups may have functional relevance to the binding mechanism

Tan et al., 2018, Cell Reports *22*, 3660–3671
March 27, 2018 © 2018 The Author(s).
https://doi.org/10.1016/j.celrep.2018.03.022

CellPress

OPEN ACCESS
CellPress

Cell Reports
Article

Two Differential Binding Mechanisms of FG-Nucleoporins and Nuclear Transport Receptors

Piau Siong Tan,[3,7] Iker Valle Aramburu,[3,7] Davide Mercadante,[4,5,7] Swati Tyagi,[3] Aritra Chowdhury,[3] Daniel Spitz,[3] Sarah L. Shammas,[6] Frauke Gräter,[4,5,*] and Edward A. Lemke[1,2,3,8,*]
[1]Departments of Biology and Chemistry, Pharmacy and Geosciences, Johannes Gutenberg-University Mainz, Johannes-von-Mullerweg 6, 55128 Mainz, Germany
[2]Institute of Molecular Biology (IMB), Ackermannweg 4, 55128 Mainz, Germany
[3]Structural and Computational Biology Unit & Cell Biology and Biophysics Unit, European Molecular Biology Laboratory (EMBL), Meyerhofstrasse 1, 69117 Heidelberg, Germany
[4]Heidelberg Institute for Theoretical Studies (HITS), Schloß-Wolfsbrunnenweg 35, 69118 Heidelberg, Germany
[5]Interdisciplinary Center for Scientific Computing, Heidelberg University, Mathematikon, Im Neuenheimer Feld 205, 69120 Heidelberg, Germany
[6]Department of New Biochemistry, University of Oxford, South Parks Road, Oxford OX1 3QU, UK
[7]These authors contributed equally
[8]Lead Contact
*Correspondence: frauke.graeter@h-its.org (F.G.), edlemke@uni-mainz.de (E.A.L.)
https://doi.org/10.1016/j.celrep.2018.03.022

SUMMARY

Phenylalanine-glycine-rich nucleoporins (FG-Nups) are intrinsically disordered proteins, constituting the selective barrier of the nuclear pore complex (NPC). Previous studies showed that nuclear transport receptors (NTRs) were found to interact with FG-Nups by forming an "archetypal-fuzzy" complex through the rapid formation and breakage of interactions with many individual FG motifs. Here, we use single-molecule studies combined with atomistic simulations to show that, in sharp contrast, FG-Nup214 undergoes a coupled reconfiguration-binding mechanism when interacting with the export receptor CRM1. Association and dissociation rate constants are more than an order of magnitude lower than in the archetypal-fuzzy complex between FG-Nup153 and NTRs. Unexpectedly, this behavior appears not to be encoded selectively into CRM1 but rather into the FG-Nup214 sequence. The same distinct binding mechanisms are unperturbed in O-linked β-N-acetylglucosamine-modified FG-Nups. Our results have implications for differential roles of distinctly spatially distributed FG-Nup•NTR interactions in the cell.

INTRODUCTION

Metazoan nuclear pore complexes (NPCs) are giant molecular complexes (~120 MDa) that are located at the nuclear envelope facilitating nucleocytoplasmic traffic of cargoes. They are formed by multiple copies of around 30 distinct proteins known as nucleoporins (Nups). Approximately one-third of the Nups contain disordered regions of variable lengths rich in phenylalanine-glycine motifs (FG motifs) (Hurt, 1988; Ori et al., 2013; Wente et al., 1992). These intrinsically disordered proteins (IDPs), also known as FG-Nups, form the permeability barrier of the NPC, which acts as a selective filter, allowing the free passage of smaller cargoes (~40 kDa) while hindering cargoes with increasing size (Timney et al., 2016). Active transport of cargoes across the NPC can only occur when they are bound to adaptor molecules known as nuclear transport receptors (NTRs) (Cook et al., 2007; Görlich and Kutay, 1999). There is still a limited understanding of how the permeability barrier is formed and of how NTRs and FG-Nups orchestrate nucleocytoplasmic transport. *In vitro* equilibrium dissociation constant measurements (K_D) between FG-Nups and most NTRs obtain high-affinity complexes (K_D in the nanomolar [nM] to micromolar [μM] range; for a review, see Aramburu and Lemke, 2017). A confounding issue has been the apparently paradoxical limit on how rapid the complex can in principle dissociate ($k_{off} = K_D \cdot k_{on}$), a certain requirement for transport, which is at odds with how fast in cells NTRs can pass the permeability barrier (Kubitscheck et al., 2005; Milles et al., 2015; Sun et al., 2013; Tu et al., 2013; Yang et al., 2004).

We previously showed that the multivalent interaction between FG-Nups and NTRs takes place via the binding of multiple low-affinity binding sites, where, despite being hydrophobic, the F residues of the FG-Nups remain surface and solvent exposed and, thus, binding prone. This permits the Nup to engage with the NTR without undergoing a strong conformational change, ultimately giving rise to an "archetypal-fuzzy" complex. Distinct features of such a complex were the absence of substantial conformational changes in structure and dynamics on the length scale as detected by single-molecule fluorescence, molecular dynamics simulations, and nuclear magnetic resonance (NMR) by several labs for even different Nup•NTR complexes from different species (Hough et al., 2015; Milles et al., 2015; Raveh et al., 2016). In addition, kinetic measurements revealed very high association rate constants (~10^9 $M^{-1}s^{-1}$), which are on a par with the described values for diffusion-limited reactions between protein

 © 2018 The Author(s).
This is an open access article under the CC BY license (http://creativecommons.org/licenses/by/4.0/).

pairs. The permeability barrier also contains high concentrations (≳ 50 mM) of FG-binding sites, so transport is essentially limited by breakage of individual FG-to-NTR-binding sites ($k_{off,individual}$). Several unbinding events must take place in order for the NTR to cross the (>30 nm-thick) barrier. Combining our measurements for the K_D and the association rate constants for constructs with different numbers of motifs, we were able to account for the effects of multivalency in order to estimate $k_{off,individual}$. The multivalency, combined with a high association rate constant, allows a tight complex to be formed between partners *in vitro*, despite a very high $k_{off,individual}$. Thus, inside the permeability barrier, an NTR can migrate by a constant, rapid exchange of individual FG motif•NTR interactions, the multiplicity of which gives rise to a form of proofreading that contributes to high selectivity of the Nup•NTR interactions. Extensive computer simulations for short peptides give a visual idea of how such an exchange might occur (Raveh et al., 2016).

The archetypal-fuzzy-binding mechanism we observed appears distinct from other well-known binding mechanisms in which the IDP undergoes a substantial conformational change upon binding by, e.g., an induced fit or conformational selection process (Csermely et al., 2010; Wright and Dyson, 2009). To include complexes where conformational changes are not substantial, we here collectively term those coupled reconfiguration-binding mechanisms.

These results, obtained for a variety of FG-Nup•NTR systems and species, might seem at odds with recent crystal structures showing a 117-amino acid (aa) fragment of FG-Nup214 (and analogously also observed for yeast) (Koyama et al., 2017) apparently docked in an extended state to the exportin CRM1 (Port et al., 2015). Ultimately one might expect this to translate into overall lower association rate constants than for a fuzzy complex, since substantial conformational changes are likely to add to the energetic barrier for binding and, depending on the binding mechanism, proper orientation of the complex might be required. Ultimately, this brings us back to the transport paradox mentioned above, which drew our attention. Crystallization might trap a specific structure, such that the X-ray structure shows a snapshot of an otherwise dynamic state. However, it is also true that FG-Nup214•CRM1•RanGTP behaves biochemically differently from other FG-Nup•NTR interactions. For instance, in many biochemical approaches (such as size exclusion, ultracentrifugation, and pull-down assays), many FG-Nup•NTR complexes cannot be stably captured, due to the high dynamics of the complex, while for FG-Nup214, it has been known that a stretch in the C-terminal domain can form what appears to be a more stable complex with CRM1 (Hutten and Kehlenbach, 2006; Koyama et al., 2017; Labokha et al., 2013).

To better understand the different experimental observations, it is thus important to know if the crystal structure trapped a specific formation of an archetypal-fuzzy complex or if the FG-Nup214•CRM1 behaves distinctly compared to the large array of FG-Nup•NTR shown to form archetypal-fuzzy complexes. In this work, we combined multi-parameter single-molecule fluorescence resonance energy transfer (smFRET), kinetic measurements, and all atom molecular dynamics (MD) simulations to obtain a more comprehensive understanding of the interaction between the FG domain of Nup214 and the NTRs. We found that this cytoplasmic Nup extensively expands upon engaging with CRM1, thus undergoing a coupled reconfiguration-binding mechanism when interacting with the NTR. Unexpectedly, we found strong indications that this unique feature of the FG-Nup214•CRM1 interaction is encoded not only into CRM1 but also into the FG-Nup214 itself. Indeed, we found that FG-Nup214 can undergo a coupled reconfiguration-binding mechanism also when bound to the canonical import receptor Importinβ. We discuss these results in the context of how the NPC exploits conceptually different binding mechanism for spatially different functions.

In addition, we know that IDPs are major targets of post-translational modifications (PTMs), which can cause a change in the structure, net charge, stability, or binding surface, regulating the interaction with their binding partners (Bah and Forman-Kay, 2016). However, the possible role that O-linked β-N-acetylglucosamine (O-GlcNAc) modifications may play in the binding of FG-Nups to NTRs is poorly understood so far. Here we provide further insights into the FG-Nup•NTR interaction mechanism of O-GlcNAc-modified FG-Nups. We observed that glycosylated FG-Nups have a more expanded conformation than the unmodified form. However, despite detectable changes to the native state, our results showed that both fundamental binding mechanisms (archetypal-fuzzy and coupled reconfiguration-binding) were not substantially altered by glycosylation, fitting into the framework that the basic transport mechanism is very robust and conserved across species (Holt et al., 1987; Labokha et al., 2013; Zhu et al., 2016).

RESULTS

Disordered FG-Nup214 Undergoes a Conformational Reconfiguration upon Binding with the NTR CRM1 and the CRM1•RanGTP Complex

We designed FG-Nup214 mutants for FRET measurements to probe the FG domain of Nup214 involved in CRM1 binding, as seen from the reported crystal structure (Port et al., 2015). FG-Nup214 was site-specifically labeled at a Cys introduced at residue position 1,905 with Alexa594-maleimide (acceptor dye) and at an incorporated unnatural aa *p*-acetylphenylalanine (AcF, aa 2,043) with Alexa488-hydroxylamine (donor dye; Figure 1A). SmFRET experiments were performed to monitor, among other parameters, changes in the FRET efficiency values (E_{FRET}), which report on the distance-dependent changes in the efficiency of energy transferred from the donor to the acceptor dye due to, for example, a conformational change (refer to the Supplemental Information for details). The 2D S versus E_{FRET} plots, obtained from pulse-interleaved excitation (PIE) (Müller et al., 2005), show the populations according to the stoichiometry (S) of the dyes (y axis, S = 1 for molecules labeled with donor only; S = 0.5 for molecules containing a donor and an acceptor dye with a 1:1 ratio; and S = 0 for molecules labeled with acceptor only). Donor- or acceptor-only population can arise from dye photophysics like bleaching and/or incomplete labeling. The population at

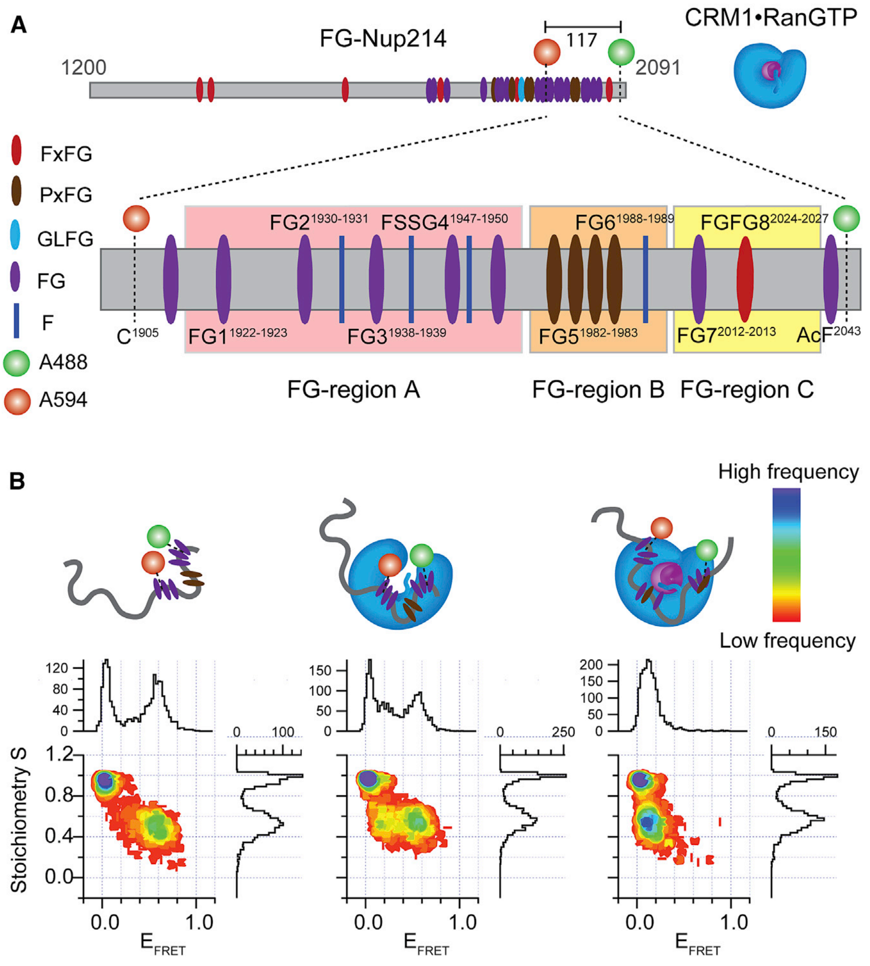

Figure 1. Conformational Change of FG-Nup214 upon Binding with CRM1 and RanGTP

(A) Scheme of FG-Nup214 construct and FG-Nup214[117], labeling sites, and CRM1•RanGTP complex. Eight FG motifs (FG1–FG8) binding to CRM1 pockets are indicated. The nomenclature of binding regions is adapted from Port et al. (2015). F residues are only shown in the zoom-in region.

(B) S versus E_{FRET} histograms of 50 pM FG-Nup214 in the absence and presence of CRM1 and CRM1•RanGTP (from left to right, at 1 μM for both CRM1 and RanGTP concentrations). In the presence of CRM1, the E_{FRET} population (at S = 0.5) shows two populations corresponding to bound and unbound forms. In the presence of RanGTP, one distinct conformation with very low E_{FRET} is seen, suggesting a much more expanded conformation than in the unbound state. See also Figures S1 and S2.

S = 0.5 is thus the one to monitor possible changes in the E_{FRET} values (x axis). E_{FRET} value shifting toward zero indicates an increase of the distance between the dyes, leading to a decrease in the efficiency of energy transferred from the donor to the acceptor dye.

Double-labeled FG-Nup214 molecules, in the absence of CRM1, showed a single FRET population with an E_{FRET} value of 0.6 (Figure 1B; Figure S1). The single peak was in line with the known fact that most IDPs are very dynamic, so that only an average distance of the rapidly fluctuating conformational ensemble was measured in smFRET (Mukhopadhyay et al., 2007). Surprisingly, if CRM1 was added in excess, we observed two FRET populations likely corresponding to an unbound (E_{FRET} = 0.6 and S = 0.5) and a bound (E_{FRET} = 0.2 and S = 0.5) state, as confirmed by photon distribution analysis (PDA; Figure S1A) (Kalinin et al., 2010). Apparently the unbound FG-Nup214 ensemble transitioned into an extended state upon binding CRM1. We note that, for signal-to-noise reasons, we could not further increase the concentration of CRM1 beyond ~4 μM in smFRET experiments. The bound fraction was substantially higher populated in the presence of RanGTP due to higher affinity of the Nup214•CRM1•RanGTP complex. The E_{FRET} value of the bound population as well as the results from the PDA were in line with the results from the crystal structure showing the probed Nup214 segment docked to the CRM1 surface (Port et al., 2015). This behavior was distinct from all of the previously measured smFRET data for various other FG-Nup•NTR complexes, which did not show any substantial change in the peak positions upon binding (see below and Milles et al., 2015). A core signature of the archetypal-fuzzy complex is, thus, not fulfilled, and this directly speaks for a binding mechanism involving a large-scale conformational change in the ensemble of the IDP, i.e., a coupled reconfiguration-binding mechanism.

Kinetic Rate Constants of FG-Nup214•CRM1 and FG-Nup214•CRM1•RanGTP Are Lower Than Other FG-Nup•NTR Binding Reactions

Stopped-flow kinetic measurements monitoring complex formation using anisotropy under pseudo-first order conditions (i.e., >10-fold excess of the NTR) were used to extract the association rate constant (k_{on}) of FG-Nup214 binding to CRM1 and CRM1•RanGTP (Figures 2A and S3, analogously to previously reported measurements for FG-Nup153•NTR; Milles et al., 2015). The observed rate (k_{obs}) at different NTR concentrations was obtained by fitting the measured traces with a single exponential decay function. The k_{on} was obtained from the slope of the k_{obs} versus NTR concentration plots. We extracted a k_{on} = (4.0 ± 0.9) × 10^7 $M^{-1}s^{-1}$ (Figures 2 and S3A) describing the binding of CRM1 with FG-Nup214. This was altered only slightly when RanGTP was pre-bound to CRM1 to (2.1 ± 0.2) × 10^7 $M^{-1}s^{-1}$ (Figure 2A). Using donor signal change for a FRET-labeled sample rather than anisotropy gave roughly consistent results ((3.0 ± 0.5) × 10^7 $M^{-1}s^{-1}$) (Figure S3C). Notably, these rate constants were roughly an order of magnitude lower than

OPEN ACCESS
CellPress

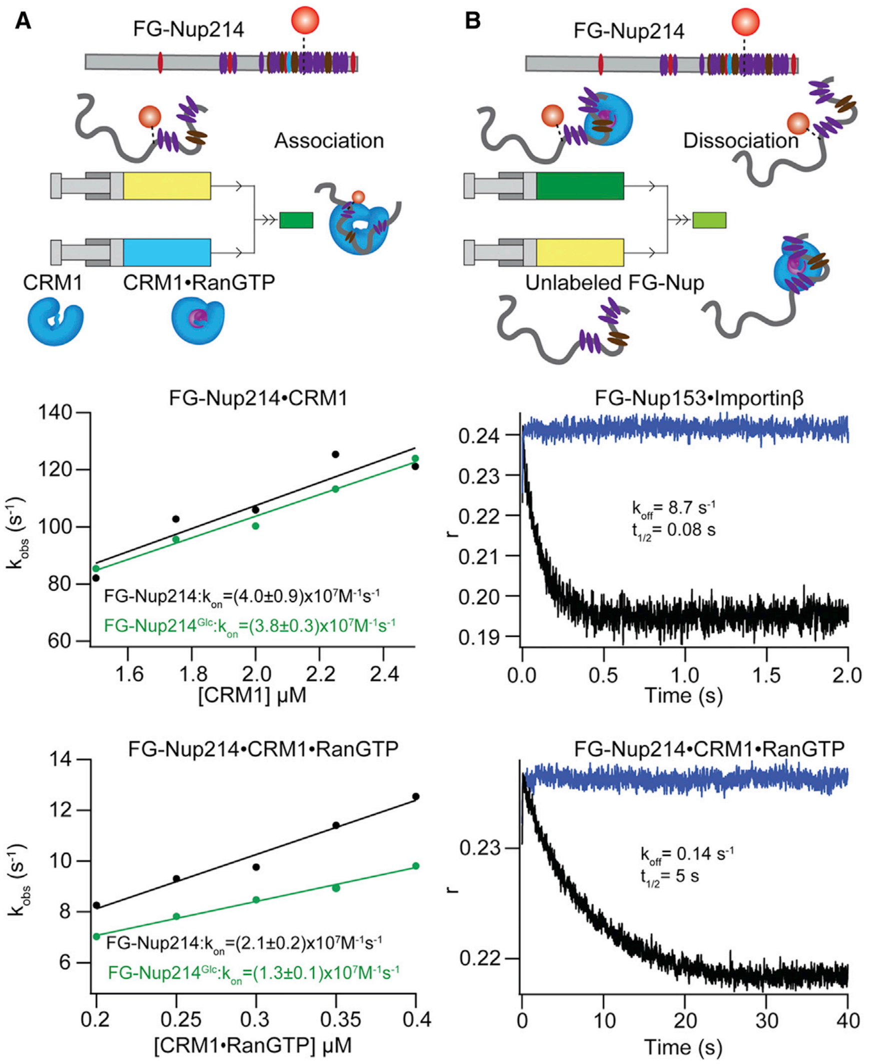

Figure 2. FG-Nup214 Has Lower Association and Dissociation Rate Constants Than FG-Nup153

(A) Kinetic association stopped-flow anisotropy measurements. Schematic drawings of mixing within stopped-flow fluorescence measurement are presented. Observed rates (k_{obs}) from anisotropy association experiments were plotted for different CRM1 concentrations, and the data were linearly fitted to obtain the association rate constant (k_{on}).

(B) Dissociation experiment. Kinetic traces obtained from the dissociation of preformed FG-Nup•NTR complex upon rapid mixing with 100× (2 μM) excess of unlabeled FG-Nup. The k_{off} for FG-Nup153•Importinβ and FG-Nup214•CRM1•RanGTP was of 8.7 s^{-1} and 0.14 s^{-1}, respectively. See also Figure S3.

the ones reported for most of the other FG-Nup•NTR interactions (~10^9 $M^{-1}s^{-1}$) (Milles et al., 2015).

We further studied the FG-Nup•NTR-binding mechanism by performing dissociation kinetic measurements (Figure 2B). The rate of complex dissociation obtained using 100× excess (2 μM) of unlabeled FG-Nup showed a k_{off} of 8.7 and 0.14 s^{-1} for our FG-Nup153•Importinβ and FG-Nup214•CRM1•RanGTP complexes, respectively, which corresponds to a ~60-fold difference in the complex half-life of 0.08 and 5 s under the same concentration of unlabeled FG-Nup (see Supplemental Information).

Glycosylated Nups Maintain Their NTR-Binding Mechanisms

In contrast to yeast, metazoan Nups are highly glycosylated. As NPC transport appears robust across species, the exact role of this omnipresent PTM remains to be better understood. Having identified two conceptually different binding mechanisms between NTRs and unglycosylated FG-Nups, we studied the influence of this PTM in the FG-Nup•NTR binding. We *in vitro* glycosylated FG-Nup214 (FG-Nup214^{Glc}) and FG-Nup153 (FG-Nup153^{Glc}) following a procedure previously developed for FG-Nup98 (Labokha et al., 2013). The *in vitro* glycosylation of FG-Nups was confirmed by SDS-PAGE, western blots, and peptide digest mass spectrometry (Figure S4).

We performed smFRET experiments under the same conditions of Figure 1 by using FG-Nup214^{Glc}. Figure 3 shows that, in particular, FG-Nup214^{Glc} (E_{FRET} = 0.3; FG-Nup153^{Glc} E_{FRET} = 0.5) had lower E_{FRET} compared to the unglycosylated FG-Nup in the unbound form, indicating expansion upon glycosylation. In contrast to the unglycosylated form, FG-Nup214^{Glc} in the presence of CRM1 yielded only a single E_{FRET} population, as validated by PDA (Figure S1A), which was similar to its unbound form (Figure 3A), indicating a reduced affinity of the complex (so that no bound fraction was populated under the chosen experimental conditions). In the presence of CRM1•RanGTP, we detected again a single population virtually identical to the unglycosylated and bound state. In contrast, FG-Nup153 yielded similar smFRET signals in the presence of Importinβ for both the glycosylated and unglycosylated forms (Figure 3B).

We then performed all measurements for the labeled fragment (117 aa, termed FG-Nup214^{117}), which probed the same region. Despite the full-length FG-Nup being approximately 55 kDa larger and containing 45 more Fs, the E_{FRET} values measured for the fragment (FG-Nup214^{117}) and the same region probed for full-length FG-Nup214 were remarkably similar in the glycosylated and unglycosylated forms (Figures 3A, S4A, and S4B). This showed that the detected effect was largely encoded into the region sandwiched between the FRET labels.

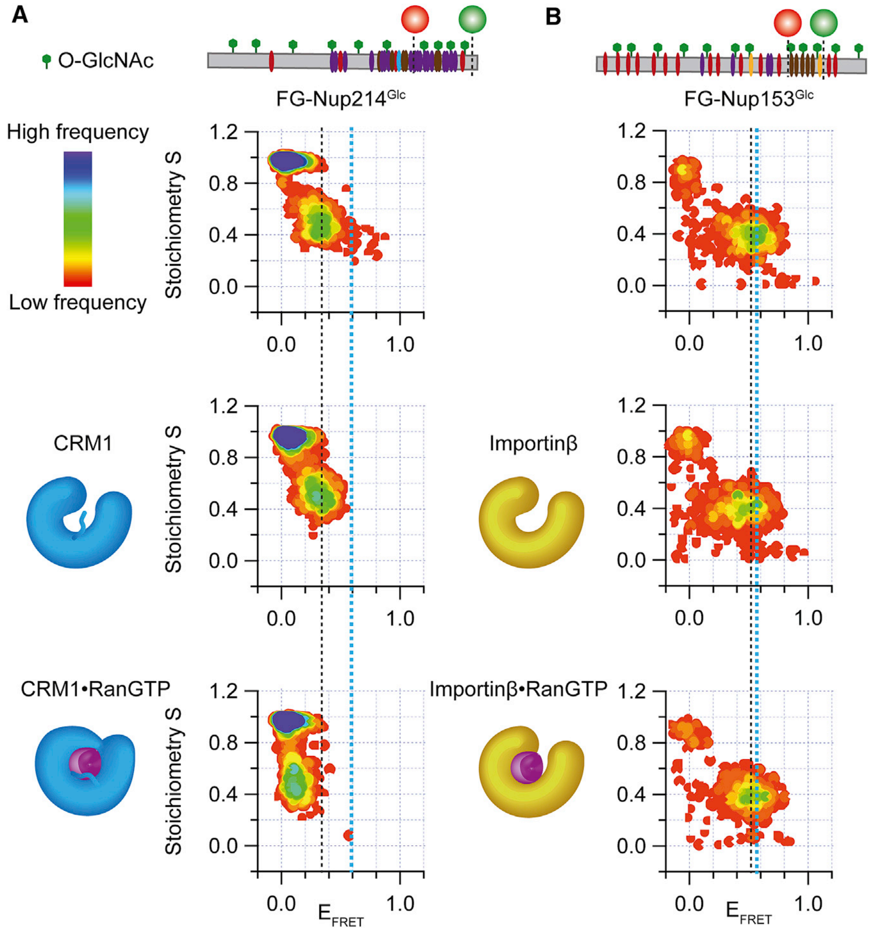

Figure 3. Conformational Features of Glycosylated FG-Nup upon Interaction with NTR

(A) E_{FRET} versus S histograms of FG-Nup214^{Glc} in the absence and presence of CRM1 and CRM1•RanGTP (from top to bottom, at 1 μM for both CRM1 and RanGTP concentrations). In the presence of CRM1, the E_{FRET} population had a similar E_{FRET} value to unbound FG-Nup. In presence of RanGTP, one distinct population with very low E_{FRET} was seen. The E_{FRET} value was remarkably similar to the bound unglycosylated case (compare to Figures 1 and 5).

(B) S versus E_{FRET} histograms of FG-Nup153^{Glc} in the absence and presence of Importinβ and Importinβ•RanGTP (from top to bottom, at 1 μM for both Importinβ and RanGTP concentrations). In particular for FG-Nup214^{Glc}, a lower E_{FRET} compared to the unglycosylated state (black dotted line versus blue dotted line) in the unbound form was detected, indicating that the glycosylated Nup is more extended. However, glycosylated and unglycosylated FG-Nups behave remarkably similarly in their respective binding modes, indicating that glycosylation only mildly tunes the binding mechanism between FG-Nups and NTRs. See also Figures S1 and S4.

We also compared the kinetics for FG-Nup214^{Glc} and FG-Nup153^{Glc} (Figure 2; Figures S3D–S3F) binding to NTRs. We obtained a k_{on} = (3.8 ± 0.3) × 10^7 $M^{-1}s^{-1}$ for the FG-Nup214^{Glc}•CRM1 interaction, (1.3 ± 0.1) × 10^7 $M^{-1}s^{-1}$ for the FG-Nup214^{Glc}•CRM1•RanGTP interaction, and (8.5 ± 1.1) × 10^8 $M^{-1}s^{-1}$ for the FG-Nup153^{Glc}•Importinβ interaction. These data indicated that there was no substantial difference between glycosylated FG-Nup and unglycosylated FG-Nup in terms of association rate constants with NTRs.

MD Simulations Support a Coupled Reconfiguration-Binding Mechanism between FG-Nup214^{117} and CRM1•RanGTP

Previous MD simulations were key in understanding the molecular architecture of the dynamic FG-Nup•NTR complexes (Milles et al., 2015; Raveh et al., 2016). To elucidate the role of different FG repeats and CRM1-binding pockets in determining the binding of the FG-Nup214^{117} to CRM1•RanGTP, we employed all-atom MD simulations. After reconstructing the parts of the complex that X-ray crystallography did not resolve (see the Experimental Procedures) (Port et al., 2015), FG-Nup214^{117} was simulated in isolation and in complex with the CRM1•RanGTP heterodimer. We performed all simulations with AMBER99sb*-ILDN and TIP4PD (Piana et al., 2015) and with the Kirkwood-Buff protein force field (Ploetz et al., 2010), both of which have been shown to yield dimensions of unfolded proteins or IDPs in line with experimental findings (Mercadante et al., 2015; Piana et al., 2015).

The analysis of the end-to-end distance (R_E) and radius of gyration (R_G) of FG-Nup214^{117} revealed a remarkable difference between the bound and unbound states for the two force fields (compare Figures S5A and S5C with S5B and S5D). Unbound FG-Nup214^{117} mostly assumed semi-compacted conformations when compared to the bound segment (Figures S5A–S5D). The expansion of the bound FG-Nup214^{117} directly agreed with the E_{FRET} increase observed upon binding of FG-Nup214^{117} to CRM1•RanGTP (Figure 1B). Our simulations confirmed that F1915, which lies at the N terminus of the FG-Nup214^{117} fragment, did not interact with CRM1, as also previously reported (Port et al., 2015). The unbound state included only a minority of conformers with R_E, R_G, and α-helical content (Figure S5E) overlapping with the range sampled in the bound state. Notably, the sequence-based prediction of intrinsic disorder for FG-Nup214^{117}, as profiled by a range of disorder predictors, suggested a lower tendency for disorder at the termini (Figure S5F). Concordantly, the X-ray crystal structure of the FG-Nup214^{117} bound to CRM1•RanGTP revealed a tendency to assume an α-helical conformation (Port et al., 2015). Our MD data corroborated the α helix propensity of FG3 in these regions in both bound and unbound states (25% versus 13%) (Figure S5G). We also found another stretch lying at the C terminus of FG-Nup214^{117}, ahead of F2024-G2025 (FG8), to form an α helix in both bound (~35%) and unbound (~15%) states

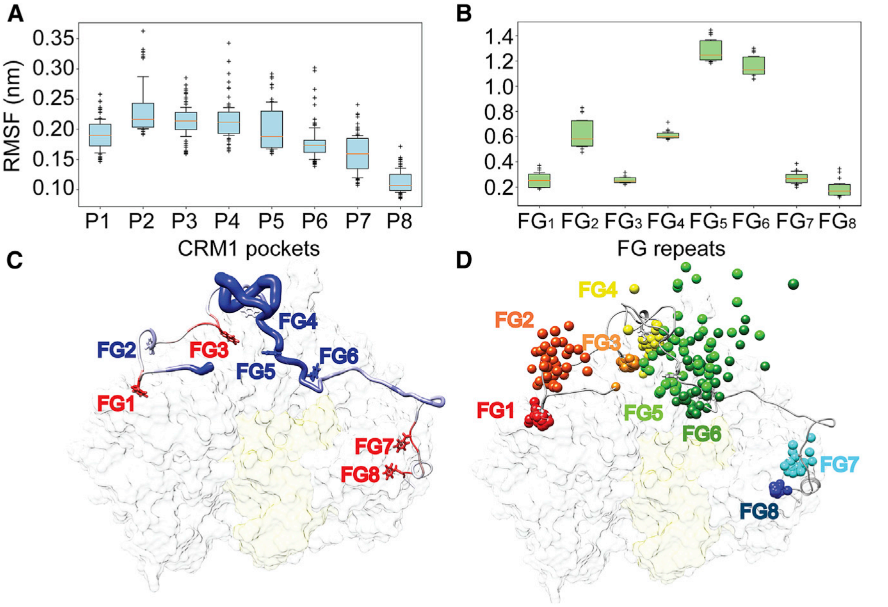

Figure 4. Dynamics of FG Repeats in the FG-Nup214^{117}•CRM1•RanGTP Complex

(A) Root-mean-squared fluctuations (RMSFs) of the CRM1 residues forming the pockets binding the FG-Nup214^{117} FG repeats.

(B) RMSFs of the FG repeats binding the CRM1 pockets (P1–P8) shown in (A). Each box indicates the range (interquartile range [IQR]) between the lower (Q1) and upper (Q3) quartile whereas the solid line represents the distribution's median. The whiskers report values that fall below Q1–Q1.5*IQR or above Q3 + 1.5*IQR. Outliers are shown as crosses below or above whiskers.

(C) Representative structure of the FG-Nup214^{117}•CRM1 complex. The width and coloring of the FG-Nup214^{117} backbone is proportional to the B factor as obtained from MD simulations, with blue for a low and red for a high B factor. The labels of the different FG repeats refer to the FG1–FG8 range described by Port et al. (2015).

(D) Dynamics of FG-Nup214^{117} FG repeats bound to CRM1. The positions of the Cζ atoms, which are part of the F rings, are represented as spheres in frames collected every 5 ns along the simulated trajectories. Spheres of different FG repeats are colored from red (N terminus) to blue (C terminus) as follows: F1922 (part of FG1), red; F1930 (part of FG2), dark orange; F1938 (part of FG3), orange; F1947 (part of FG4), yellow; F1982 (part of FG5), green; F1988 (part of FG6), dark green; F2012 (part of FG7), cyan; and F2024 (part of FG8), blue. See also Figures S5 and S6 and Movie S2.

(Figure S5G). Hence, both the FG3 and FG8 motifs experience strong conformational restraints because of the immediately flanking secondary structure elements of the otherwise disordered peptide.

MD Simulations Reveal Dynamics of FG Repeats in the FG-Nup214^{117}•CRM1•RanGTP Complex

In MD simulations of the FG-Nup214^{117}•CRM1•RanGTP complex, we observed a diverse dynamical behavior for the different pockets of CRM1 and interacting FG repeats. We observed the root-mean-squared fluctuations (RMSFs) of the CRM1 P1- to P8-binding pockets of the NTR (Port et al., 2015) to be significantly lower with the simulated fragment docked (Figure 4A), which is in line with the experimental B factors from previously collected X-ray crystallographic data (Port et al., 2015) (Figures S6A and S6B). The RMSF of the FG repeats (FG1–FG8) showed a similar trend, with FG1 and FG7/FG8 having low fluctuations and the FG repeats lying in the middle of the chain (FG4, FG5, and FG6) being characterized by a higher degree of dynamics (Movie S1). In particular, the hydrogen bond between the backbone carbonyl and amide groups of T1981 and G1984, respectively, which have been suggested to stabilize the docking of FG5 into P5 of CRM1 (Port et al., 2015), were not retained during the simulations (Figures S6C–S6F). It is worth noticing that the RMSF values recorded for FG2 suggested intermediate dynamics, compared with the FG repeats at the termini (FG1, FG7, and FG8) and FG5 or FG6 (Figure 4B), but were still incompatible with a stable binding of CRM1. This was in line with the previous observation that FG2 (F1931–G1932) interacted with CRM1 only superficially (Port et al., 2015). Conversely, FG3 (F1938–G1939) remained located in a deep pocket of CRM1, resulting in comparably little fluctuations. Fluctuations of this FG repeat were further diminished by a short α-helical segment involving this ^{1937}SFGE^{1940} stretch. Thus, only a distinct subset of FG repeats (FG1, FG3, FG7, and FG8) stably interacted with the NTR. For the others (FG2, FG4, FG5, and FG6), which retained highly fluctuating dynamics also in the bound state, we estimated $k_{off,individual}$ in the submicrosecond range and $K_{D,individual}$ in the range of 0.1–0.7 mM based on the bound and unbound fractions (Figure S6F). This range is comparable to the local $K_{D,individual}$ for FG-Nup153•Importinβ (Milles et al., 2015; Tu et al., 2013), and it underscores the dynamic nature of these FG motifs.

The FG-Nup Rather Than the NTR Dictates the Binding Mechanism

Our MD data indicated that specifics about the binding mechanism were encoded into the FG-Nup rather than into CRM1. To gain experimental support for this, we followed two approaches:

(1) The smFRET data (Figure 1B) showed a distinct conformational change of FG-Nup214 upon interacting with CRM1 and CRM1•RanGTP that did not occur in other pairs we tested (Figure 5B). We now extended our smFRET study by comparing the binding of FG-Nup153 to CRM1, as well as FG-Nup214 together with Importinβ and both with RanGTP (Figure 5A). Similar to CRM1, Importinβ also contains HEAT repeats and is a prototypical import receptor (Cook et al., 2007). Importantly, we detected a significant shift in the E_{FRET} value of FG-Nup214 when adding Importinβ, suggesting that it populated more expanded conformers. This observation is qualitatively similar to the results obtained for the FG-Nup214•CRM1 and FG-Nup214•CRM1•RanGTP complexes. In stark contrast to FG-Nup214, FG-Nup153 did

OPEN
ACCESS
CellPress

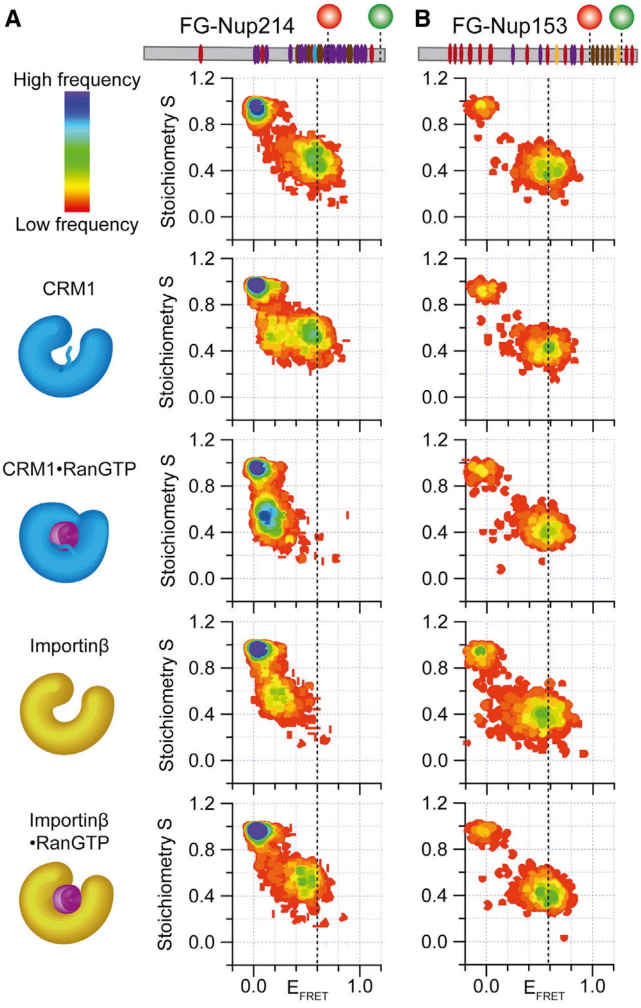

Figure 5. Conformational Feature of Different NTRs in the Presence and Absence of Different NTRs Probed by smFRET

(A and B) S versus E_{FRET} histograms of (A) FG-Nup214 and (B) FG-Nup153 in the absence and presence of different NTRs (with/without RanGTP, at 1 μM for both NTR and RanGTP concentrations). Left panel rows 1–3 are repeated from Figure 1 for comparative reasons. The black dotted line visualizes the shift of the E_{FRET} peak. The smFRET data show a distinct conformational feature of FG-Nup214 upon interaction with CRM1, Importinβ, and the CRM1•RanGTP complex. In contrast to FG-Nup214, FG-Nup153 does not undergo any conformational change, as detectable by smFRET, in the presence of Importinβ or CRM1. See also Figure S7.

not undergo any detectable conformational change in the presence of either Importinβ or CRM1, highlighting the differential behavior of FG-Nup153 and FG-Nup214 and the role of the Nups in influencing the binding mechanism to NTRs.

(2) FG-Nup sequences contain low-complexity regions and are evolutionarily not well conserved. As such, insights from bioinformatics analysis to reveal specific design features or assign motifs within FG domains are still limited compared to other classes of proteins (Ando et al., 2013; Schmidt and Görlich, 2015; Yamada et al., 2010). This is highlighted by the facts that physiological NPCs can tolerate even massive deletions of FG-Nups and encode a high level of functional redundancy (Hülsmann et al., 2012; Strawn et al., 2004). As such, mutational studies targeting only a few sites are unlikely to have a strong effect on FG-Nup function. However, prompted by the simulations, which pointed toward the relevance of α helicity in the unbound and bound states, we aimed to perturb any residual helical structure using helix-breaking mutations (by inserting proline) (Bienkiewicz et al., 2002; Schulman and Kim, 1996). We assessed the binding of FG-Nup214^{117} proline mutants A1927P/A2017P and A1927P/A2017P/A2019P, as shown in Figure 6A, to the CRM1•RanGTP complex using smFRET spectroscopy. Bound and unbound subpopulations were observed by smFRET measurements, and these subpopulations were directly monitored as a function of CRM1 concentration to obtain a K_D of the bound complex (Figure 6B). Although A2017$^{FG\text{-}Nup214}$ interacted with Y105^{CRM1} as shown by (Port et al., 2015), the double-proline mutant (A1927P/A2017P) showed only a slight increase in K_D value (from 35 ± 5 nM to 53 ± 7 nM). However, when A2019, which lies within the α helix structure, was further mutated (giving the triple mutant A1927P/A2017P/A2019P), K_D increased 5-fold over that for wild-type (WT) FG-Nup214^{117} (K_D = 222 ± 19 nM), in agreement with predictions by our MD simulations.

DISCUSSION

Active transport through the NPC requires at least three distinct steps: docking to the NPC, passage through the permeability barrier, and undocking. Export proceeds analogously in the other direction. Since electron tomography has provided a very detailed snapshot of the NPC scaffold and the stoichiometry of Nups constituting the NPC is known, the concentration of F residues in the roughly 30-nm-wide barrier is very high ($\gtrsim$ 50 mM; for reviews, see Aramburu and Lemke, 2017 and Beck and Hurt, 2017). This barrier can be passed within ~3–5 ms, as known from single-particle tracking studies that follow the trajectory of an individual NTR or cargo molecule and can, in principle, even distinguish docking, barrier passage, and undocking steps (Kubitscheck et al., 2005; Milles et al., 2015; Sun et al., 2013; Tu et al., 2013; Yang et al., 2004). Molecularly, barrier passage must thus require rapid formation and rapid breakage of Nup•NTR bonds.

Inside the permeability barrier, the concentration of Fs is so high that, even for a typical complex k_{on} of 10^5 $M^{-1}s^{-1}$, the NPC transport time will be almost independent of the motif $k_{on,individual}$. However, a naive estimate of 1 s^{-1} for k_{off}, based on typical *in vitro* K_D measurements of isolated Nup•NTR complexes (100 nM), and a k_{on} not exceeding 10^7 $M^{-1}s^{-1}$ cannot explain how even a single Nup•NTR bond can be broken during the 5 ms transport time. Recently, we found an ultrafast binding modality of FG-Nups involving multiple low-affinity binding motifs engaging in a highly dynamic manner with the different binding sites of the NTRs (Milles et al., 2015). The observed binding mechanism, termed archetypal-fuzzy, which was found for

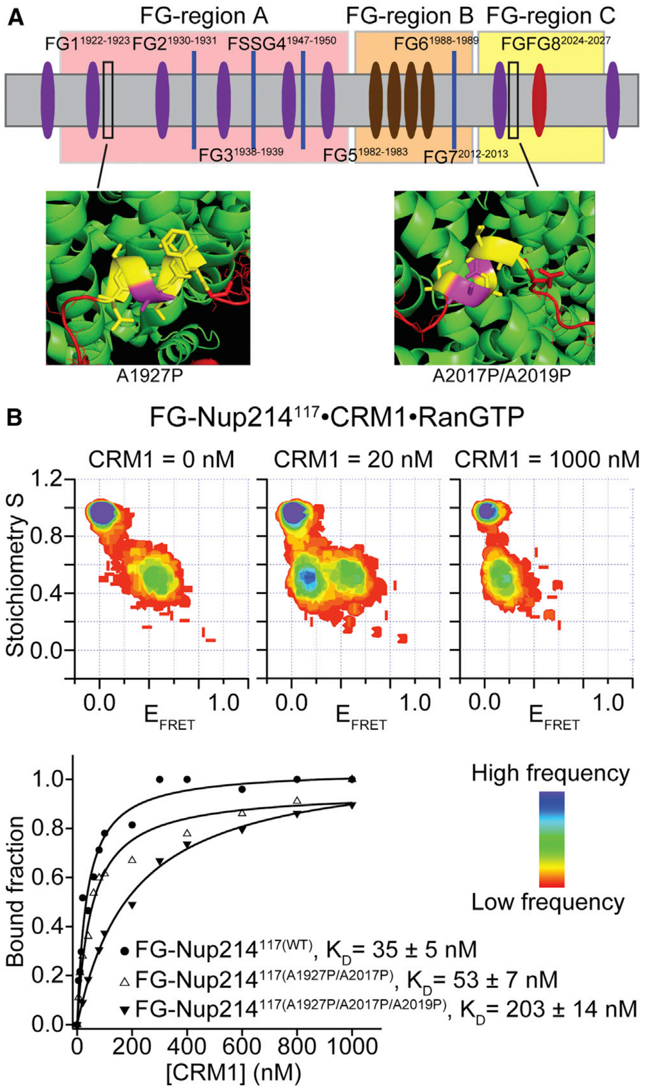

Figure 6. K_D Determination of FG-Nup214^{117} Proline Mutant CRM1•RanGTP Complex

(A) Scheme of FG-Nup214^{117} proline mutant construct and mutation sites of FG-Nup214^{117}(A1927P/A2019P) and FG-Nup214^{117}(A1927P/A2017P/A2019P).

(B) S versus E_{FRET} histograms of FG-Nup214^{117} in the presence of different concentration of CRM1 in the presence of (1 μM) RanGTP. In the presence of a low concentration of CRM1, two FRET populations (bound and unbound) were observed, and the fraction of these populations (low E_{FRET}, bound complex; high E_{FRET}, unbound complex) was directly monitored as a function of CRM1 concentration to obtain K_D of the bound complex, as shown in the lower panel.

diverse NTRs and FG-Nups across different species (human and yeast; Hough et al., 2015; Milles et al., 2015; Raveh et al., 2016), forms a dynamic archetypal-fuzzy complex in which any adopted conformation of the unbound FG-Nup is binding prone and can bind NTRs without observing a detectable change in the multiple rapidly interconverting conformations upon binding (shown as no change in the E_{FRET} values) (Milles et al., 2015). Remarkably, we observed association rate constants approaching the theoretical diffusion limit. This allows a higher k_{off} estimate inferred from the observed K_D. However, it is critical to take multivalency into account. FG-Nups like FG-Nup153 or FG-Nup214 have 60 and 62 Fs, respectively, and NTRs like Importinβ and CRM1 have 19 and 21 HEAT repeats tentatively capable of binding FG. Taking both the measured diffusion limited k_{on} and multivalency into account can bring estimates of $k_{off,individual}$ orders of magnitude higher than the measured global k_{off}, and it can explain how NTRs can pass through the permeability barrier of the NPC in millisecond timescales.

Such a fuzzy complex-binding mechanism could not have been deduced easily from available FG-Nup (peptide)•NTR crystal structures (Bayliss et al., 2000a, 2000b, 2002a, 2002b) as those rather showed snapshots of specific trapped states. Thus, a major question that now arose was, how does the detailed crystal structure showing a 117-aa Nup214 fragment docked to the CRM1•RanGTP complex fit into this picture? In this paper, we show that at least two distinct binding mechanisms exist on how an FG-Nup can interact with an NTR, i.e., an archetypal-fuzzy one and a coupled reconfiguration-binding mechanism.

Coupled Reconfiguration Binding versus Archetypal-Fuzzy Complex Formation in the NPC

In this work, we compared side by side the FG-Nup153•Importinβ with the FG-Nup214•CRM1 complex. Our smFRET data indicated that FG-Nup214 in the presence of CRM1 and the CRM1•RanGTP complex adopts a specific structure that is more extended (i.e., lower FRET and larger R_E in MD; Figures 1 and S5) than the average conformation of the disordered native ensemble of FG-Nup214 on its own. Notably, the probed 117-aa FG region behaves in this respect similarly independent if studied on its own, or within the context of the 699-residue-long region of the FG domain of Nup214 (Figures 1 and S4A). The detected differences in the smFRET data in the absence of RanGTP were due to a lower affinity of the short fragment for CRM1 (Figure S1). In the presence of CRM1 and RanGTP, a clear and distinct FRET shift occurs for both, the FG-Nup214 short fragment and full-length FG domain. This strong shift in the E_{FRET} signal was absent in all previously measured Nup•NTR complexes (Milles et al., 2015), and it argues against formation of an equally fuzzy complex.

Previously, we observed formation of a fuzzy complex characterized by extremely high association rate constants. Interestingly here, despite similar sizes and numbers of Fs, we did indeed observe a lower k_{on}. Perhaps more biologically interesting is any potential difference in the $k_{off,individual}$ that can be inferred from our experiments, since this is critical to permeability barrier passage time. The complex half-life of FG-Nup214•CRM1•RanGTP is more than 60-fold higher than FG-Nup153•Importinβ. $k_{off,global}$ is thus more than one order of magnitude lower in the case where structural rearrangements take place. This appears at odds with experimental data that suggest coupled folding and binding processes are generally typified by faster dissociation rates than those without structural rearrangement (Huang and Liu, 2009; Shammas et al., 2012). However, this comparison is with pairs of structured proteins; data for fuzzy complexes are too limited for comparison as of

yet. It is also important to note that only $k_{off,global}$ is accessible to us experimentally. This is much lower than any estimated $k_{off,individual}$, and highly dependent on the number of, and distances between, F-binding sites (see the Supplemental Experimental Procedures). Extreme caution should also be exercised when comparing rate constants for two different pairs of proteins. Our conclusion of two differential binding mechanisms is based on the synergistic results from different experimental technologies and evidences, rather than only from kinetic measurements.

MD simulations also captured the same conformational change of FG-Nup214 upon interacting with CRM1•RanGTP, providing further evidence that FG-Nup214 undergoes a conformational reconfiguration, which is substantially different from the other tested FG-Nup•NTR cases investigated before (Milles et al., 2015). Notably, the simulations (Figures S5A–S5E) show an overlapping conformational space both in terms of extent of collapse and residual secondary structure between the unbound and bound state ensembles. More specifically, already in the unbound state, FG-Nup214 samples to a minor extent also more extended conformations and structures with partial α helicity, both of which appear to be relevant for FG-Nup214•CRM1•RanGTP complex formation. We next tested with our MD simulations if anchorage and conformational propensity of FG-Nup214 at the distant CRM1 pockets might cause the observed expansion of the IDP ensemble. Indeed, the bound conformation showed differential dynamics across the binding interface, with lower dynamics and consequently stronger binding found for the moieties that anchor the terminal FG repeats of the simulated FG-Nup214 fragment. The large central part of the bound FG-Nup214 fragment seems to instead play a minor role in defining formation and stability of the complex. This dynamic and very heterogeneous central region also included the three repeats FG4–FG6, which were among those well resolved within CRM1 pockets in the X-ray structure (Port et al., 2015) (see Movie S2 and Figures S6C–S6F). This underlines that, despite all FG regions being resolved in the crystal structure, their dynamic behavior can be completely different. We conclude that coupled reconfiguration binding in the case of FG-Nup214 and CRM1 also includes fuzzy and transient FG•CRM1 interactions, with local $K_{D,individual}$ similar to those of FG-Nup153•Importinβ. Movie S2 visualizes how an FG motif in the middle of the 117-aa fragment constantly binds and unbinds on the very short nanosecond timescale, in agreement with ultrafast kinetics. The termini of the 117-aa Nup214 appear largely responsible for locking the peptide onto the CRM1 surface. The co-existence of these two binding modes in very close proximity probably compensates for the entropic penalty that may be generated from the reduced conformational freedom of FG-Nup214 upon CRM1 binding (Marlow et al., 2010; Tzeng and Kalodimos, 2012).

Spatial Segregation of Distinct Binding Mechanisms in the NPC

In the central channel of the NPC, where the permeability barrier is formed by high densities of FG-Nups, a tight clamping mechanism with its associated kinetics is not favorable, because it would reduce the transport efficiency of cargo passage substantially. This points to a unique role of the FG-Nup214•CRM1 interaction in the NPC mechanism in line with previous observations (Hutten and Kehlenbach, 2006; Labokha et al., 2013). FG-Nup214 localizes to the cytoplasmic side of the NPC, and it is most likely not a key component of the permeability barrier of the central channel at the NPC but rather may have other functionalities. For example, the N-terminal folded domain of Nup214 has been shown to be required for the recruitment of the DEAD-box helicase Ddx19 involved in the messenger ribonucleoprotein particles (mRNPs) remodeling (Napetschnig et al., 2009). Moreover, it has been suggested that Nup214 takes part in the last steps of the nuclear export of cargoes (Kehlenbach et al., 1999). In addition, depletion of Nup214 has been reported to cause the inhibition of some CRM1 export cargoes (Bernad et al., 2006), and RNAi downregulation of FG-Nups in S2 *D. melanogaster* cells showed that different FG domains play distinct roles in the nucleocytoplasmic transport (Sabri et al., 2007).

Altogether, we can speculate that free CRM1 can bind and unbind and move rapidly through Nups in the central channel. However, when CRM1 is forming part of the export complex (bound to RanGTP), it will specifically and tightly bind to the C-terminal region of FG-Nup214 once it reaches the cytoplasmic face of the NPC and form a longer-lived complex, which is in good agreement with our dissociation kinetic experiment. In this way, CRM1•RanGTP may dock on FG-Nup214 at the C-terminal position, and they may get in close proximity to Ran-binding protein 2 (RanBP2), which has also been shown to bind strongly to CRM1•RanGTP with two FG regions 300 aas apart (Ritterhoff et al., 2016), in order to undergo GTP hydrolysis and cargo release (Port et al., 2015). In addition, competition experiments have shown that the Ran-binding protein RanBP3 that facilitates the formation of the export complex is also able to displace FG-Nup214 from the CRM1•RanGTP complex (Port et al., 2015). Further studies showed that two FG regions located at the disordered domain of Yrb2p, the yeast homolog of RanBP3, bind to the C- and N-terminal sites of Xpo1p, the yeast homolog of CRM1 (Koyama et al., 2014). This might indicate that RanBP3 interacts with CRM1•RanGTP in a similar binding mechanism as FG-Nup214. Thus, CRM1 release from FG-Nup214 is subject to tight biochemical control instead of ultrafast spontaneous dissociation.

Coupled Reconfiguration Binding of Nup214 Is Not Unique to CRM1 Complex Formation

The NPC is made from several FG-Nups, but what sequence characteristics give rise to a specific function is not well understood, and, indeed, minimal functional pores can even be built from few FG-Nups (Hülsmann et al., 2012; Strawn et al., 2004). FG-Nups are only poorly evolutionarily conserved (Ando et al., 2013; Denning and Rexach, 2007; Schmidt and Görlich, 2015; Yamada et al., 2010), and many have large low-complexity regions. Identifying unique features in these large disordered molecules is thus a complex venture. Our MD simulations showed that one factor determining tight binding of FG repeats appears to be the formation of short helical structures adjacent to the FG (FG3 and FG8) in both bound and unbound states. This suggests that anchorage, i.e., coupled reconfiguration

OPEN
ACCESS
CellPress

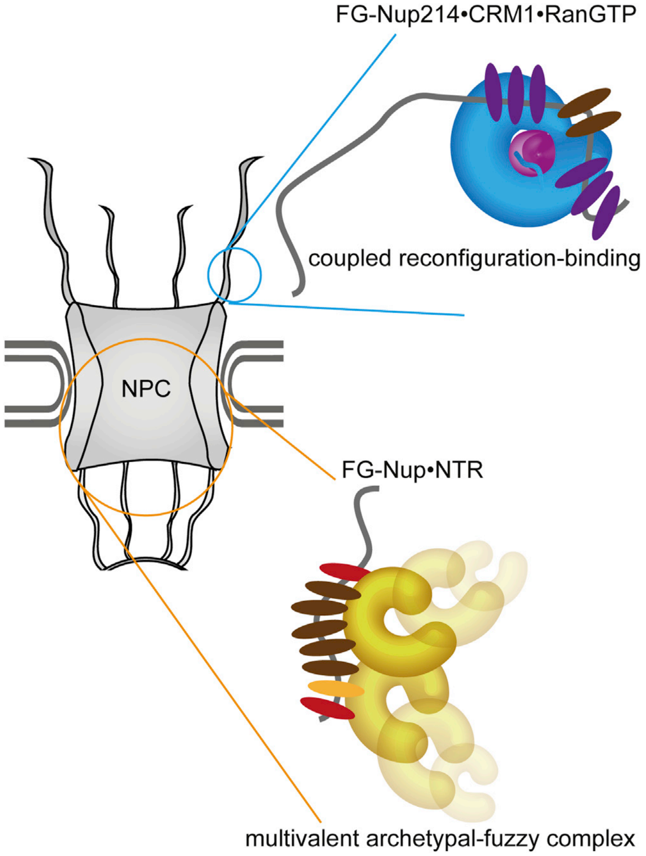

Figure 7. Differential Binding Modes of FG-Nup●NTR Interactions
Disordered FG-Nup214 undergoes a coupled reconfiguration binding with the NTR CRM1•RanGTP complex, in contrast to the previously reported archetypal-fuzzy binding mechanism of FG-Nup153•Importinβ, indicating that variations in the type and extent of individual FG•NTR interactions can drastically change the mechanism of binding between the FG-Nup and its NTR.

binding, might only to a minor extent depend on the nature of the CRM1 pockets but primarily be achieved through unique characteristics of the Nup214, such as a sequence-encoded propensity for transient secondary structure. Such a propensity in exploring α-helical conformations that would lock more tightly FG repeats at the terminal ends of the fragment bound to CRM1 is predicted based on the FG-Nup214 sequence as well as on the sampling of the bound and unbound state dynamics (Figures S5 and S6). The relevance of α-helical propensity in regions next to the two terminal FG motifs was further supported by our proline mutational analysis (Figure 6). While further experiments and ideally more systems need to be identified and compared, our current data put forward the notion that the FG-Nup and its sequence propensity more strongly dictate the binding mechanism than the nature of the NTR pockets. Indeed and unexpectedly, we observed that FG-Nup214 undergoes a conformational change also when binding to Importinβ (Figure 5A), yielding even a very similar E_{FRET} value as when binding to CRM1. In the Supplemental Experimental Procedures, we show further experiments that reveal that we can also detect time- and concentration-dependent aggregation phenomena in the FG-Nup•NTR complex (Figure S7), highlighting that, in such multivalent systems, more complex processes (such as phase separation) can occur as well. However, the very low concentrations (in the picomolar [pM] range) used in our single-molecule assays provide a means to deal with this complexity under the chosen experimental conditions.

The Coupled Reconfiguration-Binding Mechanism and the Archetypal-Fuzzy Complex Formation Are Robust with Respect to Glycosylation

Recent studies showed that glycosylation, an omnipresent PTM in metazoan NPCs but not yeast, may play a role in regulating nucleocytoplasmic transport, and FG-Nup214 has been shown to be heavily glycosylated in the NPC (Favreau et al., 1996; Khidekel et al., 2007; Zhu et al., 2016). If and how glycosylation affects the FG-Nup•NTR-binding mechanism is still not well understood. From the smFRET measurement shown in Figure 3, we observed that FG-Nup214^{Glc} and FG-Nup153^{Glc} in the unbound state are more expanded. This expansion upon glycosylation is likely due to an increased steric hindrance by the multiple bulky sugar chains, an effect that here overcompensates any impaired solvation of the semi-collapsed Nup214 (Cheng et al., 2010). Despite the conformational change upon glycosylation, a shift in the FRET population of FG-Nup214^{Glc} upon binding to CRM1•RanGTP was detected (Figures 3 and S4). This suggests that, while glycosylation might tune the affinity of the complex, the binding mechanism itself is robust. Analogously, we did not observe a substantial change in the data for the complex formation of FG-Nup153 to Importinβ (Figure 3B). We conclude that, for the study of the binding mechanism, unglycosylated proteins appear to be a reasonable mimic.

We summarize that in the NPC at least two fundamentally different binding mechanisms can exist between FG-Nups and NTRs (Figure 7). Formation of archetypal-fuzzy complexes is associated with fast yet selective transport through highly concentrated FG-rich channels. Potentially FG-Nup214, which forms a complex with CRM1•RanGTP by a coupled reconfiguration-binding mechanism, can help to achieve a stable spatial localization that helps undocking processes.

From an IDP biophysics perspective, our study sheds light on the diverse dynamics of IDPs, and it provides an example of how different FG-rich disordered stretches can use completely different binding mechanisms to bind to NTRs in order to gain a new functionality. However, it is not yet clear what is the basis of such biphasic behavior, whether it is subtleties in sequence, motif spacing, or other dynamic parameters. A better understanding of the architectural design of FG-Nups and what gives a certain sequence a specific role is currently complicated by our generally limited understanding of sequence space design. In particular, the high enrichment of low-complexity regions in FG-Nups and many other IDPs, which in many cases are also not evolutionarily conserved, makes identifying specific key residues in fact unlikely, as it is rather a collective property emerging for a specific area or even during assembly of a larger complex that can give rise to unique function. Larger systematic efforts in which many parameters are varied to find the essential ingredients that make an FG-Nup sequence special will be

needed. This will be complicated but also intriguing because physiological NPCs can tolerate massive deletions of FG-Nups and encode a high level of redundancy.

EXPERIMENTAL PROCEDURES

Protein Expression, Purification, Glycosylation, and Labeling

Proteins were expressed in *E. coli* BL21 DE3 AI cell and purified using standard Nickel affinity and size exclusion chromatography purification procedures. *In vitro* glycosylation and labeling were performed as described in the Supplemental Experimental Procedures.

smFRET Measurement

The smFRET experiments were performed using a home-built confocal-based microscope, and the data analysis was performed by using a custom written Igor Program (Wavemetrics, USA). The detailed information is described in the Supplemental Experimental Procedures.

Fluorescence Stopped-Flow Measurement

The kinetic experiments were performed using stopped-flow fluorescence spectroscopy (SFM-3000, Bio-logic, France) with the uFC-08 micro-cuvette accessory. Excitation was performed with a custom polarized LASER excitation source (532 nm), and polarized emission was detected using emission filters with a 538- to 642-nm bandwidth. The detailed information is described in the Supplemental Experimental Procedures.

Molecular Dynamics Simulations of FG-Nup214^{117} and FG-Nup214^{117}·CRM1·RanGTP Complex

The dynamics of the intrinsically disordered Nup alone and in complex with the CRM1•RanGTP complex were sampled using GROMACS 2016, as described in the Supplemental Experimental Procedures.

SUPPLEMENTAL INFORMATION

Supplemental Information includes Supplemental Experimental Procedures, seven figures, and two movies and can be found with this article online at https://doi.org/10.1016/j.celrep.2018.03.022.

ACKNOWLEDGMENTS

We are grateful for the fruitful discussions, comments, and feedback from Lemke group. P.S.T. is supported by a fellowship from the EMBL Interdisciplinary Postdoc (EIPOD) program under Marie Curie Actions COFUND program (grant number 291772). I.V.A. and E.A.L. acknowledge funding from the ERC grant SMPF v2.0 and from the Gutenberg Forschungskolleg. D.M. is supported by a BIOMS postdoctoral program. F.G. acknowledges the support of the Klaus Tschira Stiftung. S.L.S. is supported by an MRC Career Development Award (MR/N024168/1). We are grateful to the EMBL Proteomics core facility for help in sample analysis.

AUTHOR CONTRIBUTIONS

P.S.T., I.V.A., and E.A.L. designed and performed experiments and co-wrote the manuscript. D.M. and F.G. designed and performed simulations and co-wrote the manuscript. S.T., A.C., D.S., and S.L.S. contributed analytical tools and/or reagents. All authors discussed and analyzed data. F.G. and E.A.L. conceived the project and co-wrote the manuscript.

DECLARATION OF INTERESTS

The authors declare no competing interests.

Received: November 2, 2017
Revised: January 30, 2018
Accepted: March 6, 2018
Published: March 27, 2018

REFERENCES

Ando, D., Colvin, M., Rexach, M., and Gopinathan, A. (2013). Physical motif clustering within intrinsically disordered nucleoporin sequences reveals universal functional features. PLoS ONE *8*, e73831.

Aramburu, I.V., and Lemke, E.A. (2017). Floppy but not sloppy: Interaction mechanism of FG-nucleoporins and nuclear transport receptors. Semin. Cell Dev. Biol. *68*, 34–41.

Bah, A., and Forman-Kay, J.D. (2016). Modulation of Intrinsically Disordered Protein Function by Post-translational Modifications. J. Biol. Chem. *291*, 6696–6705.

Bayliss, R., Kent, H.M., Corbett, A.H., and Stewart, M. (2000a). Crystallization and initial X-ray diffraction characterization of complexes of FxFG nucleoporin repeats with nuclear transport factors. J. Struct. Biol. *131*, 240–247.

Bayliss, R., Littlewood, T., and Stewart, M. (2000b). Structural basis for the interaction between FxFG nucleoporin repeats and importin-beta in nuclear trafficking. Cell *102*, 99–108.

Bayliss, R., Leung, S.W., Baker, R.P., Quimby, B.B., Corbett, A.H., and Stewart, M. (2002a). Structural basis for the interaction between NTF2 and nucleoporin FxFG repeats. EMBO J. *21*, 2843–2853.

Bayliss, R., Littlewood, T., Strawn, L.A., Wente, S.R., and Stewart, M. (2002b). GLFG and FxFG nucleoporins bind to overlapping sites on importin-beta. J. Biol. Chem. *277*, 50597–50606.

Beck, M., and Hurt, E. (2017). The nuclear pore complex: understanding its function through structural insight. Nat. Rev. Mol. Cell Biol. *18*, 73–89.

Bernad, R., Engelsma, D., Sanderson, H., Pickersgill, H., and Fornerod, M. (2006). Nup214-Nup88 nucleoporin subcomplex is required for CRM1-mediated 60 S preribosomal nuclear export. J. Biol. Chem. *281*, 19378–19386.

Bienkiewicz, E.A., Adkins, J.N., and Lumb, K.J. (2002). Functional consequences of preorganized helical structure in the intrinsically disordered cell-cycle inhibitor p27(Kip1). Biochemistry *41*, 752–759.

Cheng, S., Edwards, S.A., Jiang, Y., and Gräter, F. (2010). Glycosylation enhances peptide hydrophobic collapse by impairing solvation. ChemPhysChem *11*, 2367–2374.

Cook, A., Bono, F., Jinek, M., and Conti, E. (2007). Structural biology of nucleocytoplasmic transport. Annu. Rev. Biochem. *76*, 647–671.

Csermely, P., Palotai, R., and Nussinov, R. (2010). Induced fit, conformational selection and independent dynamic segments: an extended view of binding events. Trends Biochem. Sci. *35*, 539–546.

Denning, D.P., and Rexach, M.F. (2007). Rapid evolution exposes the boundaries of domain structure and function in natively unfolded FG nucleoporins. Mol. Cell. Proteomics *6*, 272–282.

Favreau, C., Worman, H.J., Wozniak, R.W., Frappier, T., and Courvalin, J.C. (1996). Cell cycle-dependent phosphorylation of nucleoporins and nuclear pore membrane protein Gp210. Biochemistry *35*, 8035–8044.

Görlich, D., and Kutay, U. (1999). Transport between the cell nucleus and the cytoplasm. Annu. Rev. Cell Dev. Biol. *15*, 607–660.

Holt, G.D., Snow, C.M., Senior, A., Haltiwanger, R.S., Gerace, L., and Hart, G.W. (1987). Nuclear pore complex glycoproteins contain cytoplasmically disposed O-linked N-acetylglucosamine. J. Cell Biol. *104*, 1157–1164.

Hough, L.E., Dutta, K., Sparks, S., Temel, D.B., Kamal, A., Tetenbaum-Novatt, J., Rout, M.P., and Cowburn, D. (2015). The molecular mechanism of nuclear transport revealed by atomic-scale measurements. eLife *4*, e10027.

Huang, Y., and Liu, Z. (2009). Kinetic advantage of intrinsically disordered proteins in coupled folding-binding process: a critical assessment of the "fly-casting" mechanism. J. Mol. Biol. *393*, 1143–1159.

Hülsmann, B.B., Labokha, A.A., and Görlich, D. (2012). The permeability of reconstituted nuclear pores provides direct evidence for the selective phase model. Cell *150*, 738–751.

Hurt, E.C. (1988). A novel nucleoskeletal-like protein located at the nuclear periphery is required for the life cycle of Saccharomyces cerevisiae. EMBO J. *7*, 4323–4334.

Hutten, S., and Kehlenbach, R.H. (2006). Nup214 is required for CRM1-dependent nuclear protein export in vivo. Mol. Cell. Biol. *26*, 6772–6785.

Kalinin, S., Valeri, A., Antonik, M., Felekyan, S., and Seidel, C.A.M. (2010). Detection of structural dynamics by FRET: a photon distribution and fluorescence lifetime analysis of systems with multiple states. J. Phys. Chem. B *114*, 7983–7995.

Kehlenbach, R.H., Dickmanns, A., Kehlenbach, A., Guan, T., and Gerace, L. (1999). A role for RanBP1 in the release of CRM1 from the nuclear pore complex in a terminal step of nuclear export. J. Cell Biol. *145*, 645–657.

Khidekel, N., Ficarro, S.B., Clark, P.M., Bryan, M.C., Swaney, D.L., Rexach, J.E., Sun, Y.E., Coon, J.J., Peters, E.C., and Hsieh-Wilson, L.C. (2007). Probing the dynamics of O-GlcNAc glycosylation in the brain using quantitative proteomics. Nat. Chem. Biol. *3*, 339–348.

Koyama, M., Shirai, N., and Matsuura, Y. (2014). Structural insights into how Yrb2p accelerates the assembly of the Xpo1p nuclear export complex. Cell Rep. *9*, 983–995.

Koyama, M., Hirano, H., Shirai, N., and Matsuura, Y. (2017). Crystal structure of the Xpo1p nuclear export complex bound to the SxFG/PxFG repeats of the nucleoporin Nup42p. Genes Cells *22*, 861–875.

Kubitscheck, U., Grünwald, D., Hoekstra, A., Rohleder, D., Kues, T., Siebrasse, J.P., and Peters, R. (2005). Nuclear transport of single molecules: dwell times at the nuclear pore complex. J. Cell Biol. *168*, 233–243.

Labokha, A.A., Gradmann, S., Frey, S., Hülsmann, B.B., Urlaub, H., Baldus, M., and Görlich, D. (2013). Systematic analysis of barrier-forming FG hydrogels from Xenopus nuclear pore complexes. EMBO J. *32*, 204–218.

Marlow, M.S., Dogan, J., Frederick, K.K., Valentine, K.G., and Wand, A.J. (2010). The role of conformational entropy in molecular recognition by calmodulin. Nat. Chem. Biol. *6*, 352–358.

Mercadante, D., Milles, S., Fuertes, G., Svergun, D.I., Lemke, E.A., and Gräter, F. (2015). Kirkwood-Buff Approach Rescues Overcollapse of a Disordered Protein in Canonical Protein Force Fields. J. Phys. Chem. B *119*, 7975–7984.

Milles, S., Mercadante, D., Aramburu, I.V., Jensen, M.R., Banterle, N., Koehler, C., Tyagi, S., Clarke, J., Shammas, S.L., Blackledge, M., et al. (2015). Plasticity of an ultrafast interaction between nucleoporins and nuclear transport receptors. Cell *163*, 734–745.

Mukhopadhyay, S., Krishnan, R., Lemke, E.A., Lindquist, S., and Deniz, A.A. (2007). A natively unfolded yeast prion monomer adopts an ensemble of collapsed and rapidly fluctuating structures. Proc. Natl. Acad. Sci. USA *104*, 2649–2654.

Müller, B.K., Zaychikov, E., Bräuchle, C., and Lamb, D.C. (2005). Pulsed interleaved excitation. Biophys. J. *89*, 3508–3522.

Napetschnig, J., Kassube, S.A., Debler, E.W., Wong, R.W., Blobel, G., and Hoelz, A. (2009). Structural and functional analysis of the interaction between the nucleoporin Nup214 and the DEAD-box helicase Ddx19. Proc. Natl. Acad. Sci. USA *106*, 3089–3094.

Ori, A., Banterle, N., Iskar, M., Andrés-Pons, A., Escher, C., Khanh Bui, H., Sparks, L., Solis-Mezarino, V., Rinner, O., Bork, P., et al. (2013). Cell type-specific nuclear pores: a case in point for context-dependent stoichiometry of molecular machines. Mol. Syst. Biol. *9*, 648.

Piana, S., Donchev, A.G., Robustelli, P., and Shaw, D.E. (2015). Water dispersion interactions strongly influence simulated structural properties of disordered protein states. J. Phys. Chem. B *119*, 5113–5123.

Ploetz, E.A., Bentenitis, N., and Smith, P.E. (2010). Developing Force Fields from the Microscopic Structure of Solutions. Fluid Phase Equilib. *290*, 43.

Port, S.A., Monecke, T., Dickmanns, A., Spillner, C., Hofele, R., Urlaub, H., Ficner, R., and Kehlenbach, R.H. (2015). Structural and Functional Characterization of CRM1-Nup214 Interactions Reveals Multiple FG-Binding Sites Involved in Nuclear Export. Cell Rep. *13*, 690–702.

Raveh, B., Karp, J.M., Sparks, S., Dutta, K., Rout, M.P., Sali, A., and Cowburn, D. (2016). Slide-and-exchange mechanism for rapid and selective transport through the nuclear pore complex. Proc. Natl. Acad. Sci. USA *113*, E2489–E2497.

Ritterhoff, T., Das, H., Hofhaus, G., Schröder, R.R., Flotho, A., and Melchior, F. (2016). The RanBP2/RanGAP1*SUMO1/Ubc9 SUMO E3 ligase is a disassembly machine for Crm1-dependent nuclear export complexes. Nat. Commun. *7*, 11482.

Sabri, N., Roth, P., Xylourgidis, N., Sadeghifar, F., Adler, J., and Samakovlis, C. (2007). Distinct functions of the Drosophila Nup153 and Nup214 FG domains in nuclear protein transport. J. Cell Biol. *178*, 557–565.

Schmidt, H.B., and Görlich, D. (2015). Nup98 FG domains from diverse species spontaneously phase-separate into particles with nuclear pore-like permselectivity. eLife *4*, e04251.

Schulman, B.A., and Kim, P.S. (1996). Proline scanning mutagenesis of a molten globule reveals non-cooperative formation of a protein's overall topology. Nat. Struct. Biol. *3*, 682–687.

Shammas, S.L., Rogers, J.M., Hill, S.A., and Clarke, J. (2012). Slow, reversible, coupled folding and binding of the spectrin tetramerization domain. Biophys. J. *103*, 2203–2214.

Strawn, L.A., Shen, T., Shulga, N., Goldfarb, D.S., and Wente, S.R. (2004). Minimal nuclear pore complexes define FG repeat domains essential for transport. Nat. Cell Biol. *6*, 197–206.

Sun, C., Fu, G., Ciziene, D., Stewart, M., and Musser, S.M. (2013). Choreography of importin-α/CAS complex assembly and disassembly at nuclear pores. Proc. Natl. Acad. Sci. USA *110*, E1584–E1593.

Timney, B.L., Raveh, B., Mironska, R., Trivedi, J.M., Kim, S.J., Russel, D., Wente, S.R., Sali, A., and Rout, M.P. (2016). Simple rules for passive diffusion through the nuclear pore complex. J. Cell Biol. *215*, 57–76.

Tu, L.C., Fu, G., Zilman, A., and Musser, S.M. (2013). Large cargo transport by nuclear pores: implications for the spatial organization of FG-nucleoporins. EMBO J. *32*, 3220–3230.

Tzeng, S.R., and Kalodimos, C.G. (2012). Protein activity regulation by conformational entropy. Nature *488*, 236–240.

Wente, S.R., Rout, M.P., and Blobel, G. (1992). A new family of yeast nuclear pore complex proteins. J. Cell Biol. *119*, 705–723.

Wright, P.E., and Dyson, H.J. (2009). Linking folding and binding. Curr. Opin. Struct. Biol. *19*, 31–38.

Yamada, J., Phillips, J.L., Patel, S., Goldfien, G., Calestagne-Morelli, A., Huang, H., Reza, R., Acheson, J., Krishnan, V.V., Newsam, S., et al. (2010). A bimodal distribution of two distinct categories of intrinsically disordered structures with separate functions in FG nucleoporins. Mol. Cell. Proteomics *9*, 2205–2224.

Yang, W., Gelles, J., and Musser, S.M. (2004). Imaging of single-molecule translocation through nuclear pore complexes. Proc. Natl. Acad. Sci. USA *101*, 12887–12892.

Zhu, Y., Liu, T.W., Madden, Z., Yuzwa, S.A., Murray, K., Cecioni, S., Zachara, N., and Vocadlo, D.J. (2016). Post-translational O-GlcNAcylation is essential for nuclear pore integrity and maintenance of the pore selectivity filter. J. Mol. Cell Biol. *8*, 2–16.

Cell Reports, Volume *22*

Supplemental Information

Two Differential Binding Mechanisms

of FG-Nucleoporins and Nuclear Transport Receptors

Piau Siong Tan, Iker Valle Aramburu, Davide Mercadante, Swati Tyagi, Aritra Chowdhury, Daniel Spitz, Sarah L. Shammas, Frauke Gräter, and Edward A. Lemke

Figure S1

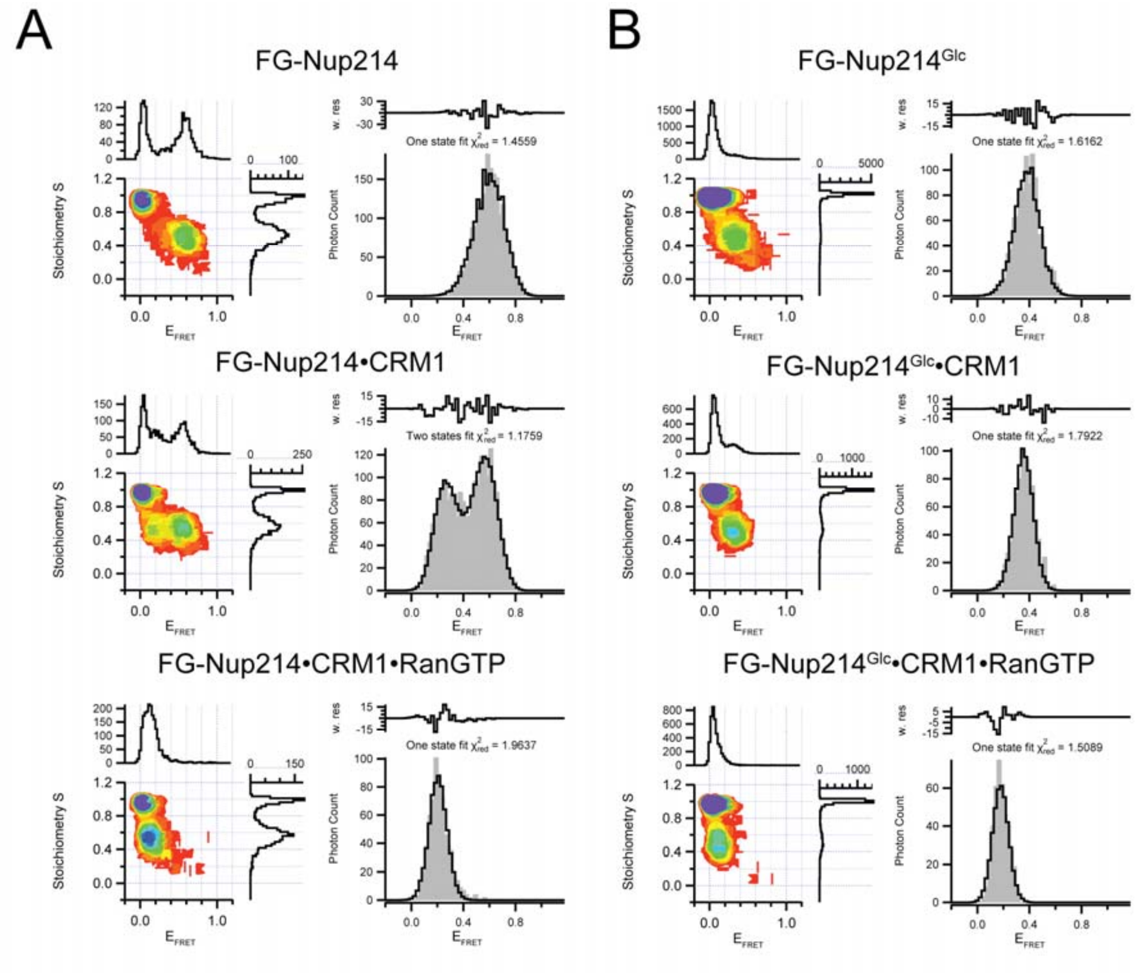

Figure S1. Photon distribution analysis (PDA) histograms, to estimate number of species corresponding to a FRET population, Related to Figure 1 and 3.

Photon distribution analysis (PDA) approach, a method that quantitatively and precisely describes the smFRET distribution, was implemented to analyse data with potentially coexisting multiple species (Kalinin et al., 2010). PDA histograms (expressed as uncorrected E_{FRET}) of (A) FG-Nup214 and (B) FG-Nup214^{Glc} in the presence of CRM1 and CRM1•RanGTP (from top to bottom). PDA confirms that two species are hidden behind the broad population of FG-Nup214•CRM1 interaction and a single species upon adding RanGTP. This suggests that FG-Nup214 undergoes a drastic conformational change upon binding the CRM1•RanGTP and the ensemble collapses into conformations with a rather fixed dye to dye distance in the bound state in agreement with the crystal structure of (Port et al., 2015). Upon interacting with CRM1, FG-Nup214^{Glc} shows a single population, which is similar to its unbound form, in terms of E_{FRET}, while in the additional presence of RanGTP, the same E_{FRET} is reached as for the unglycosylated case. This indicates lower affinity of FG-Nup214^{Glc} to CRM1, but that the overall binding mechanism is conserved.

Figure S2

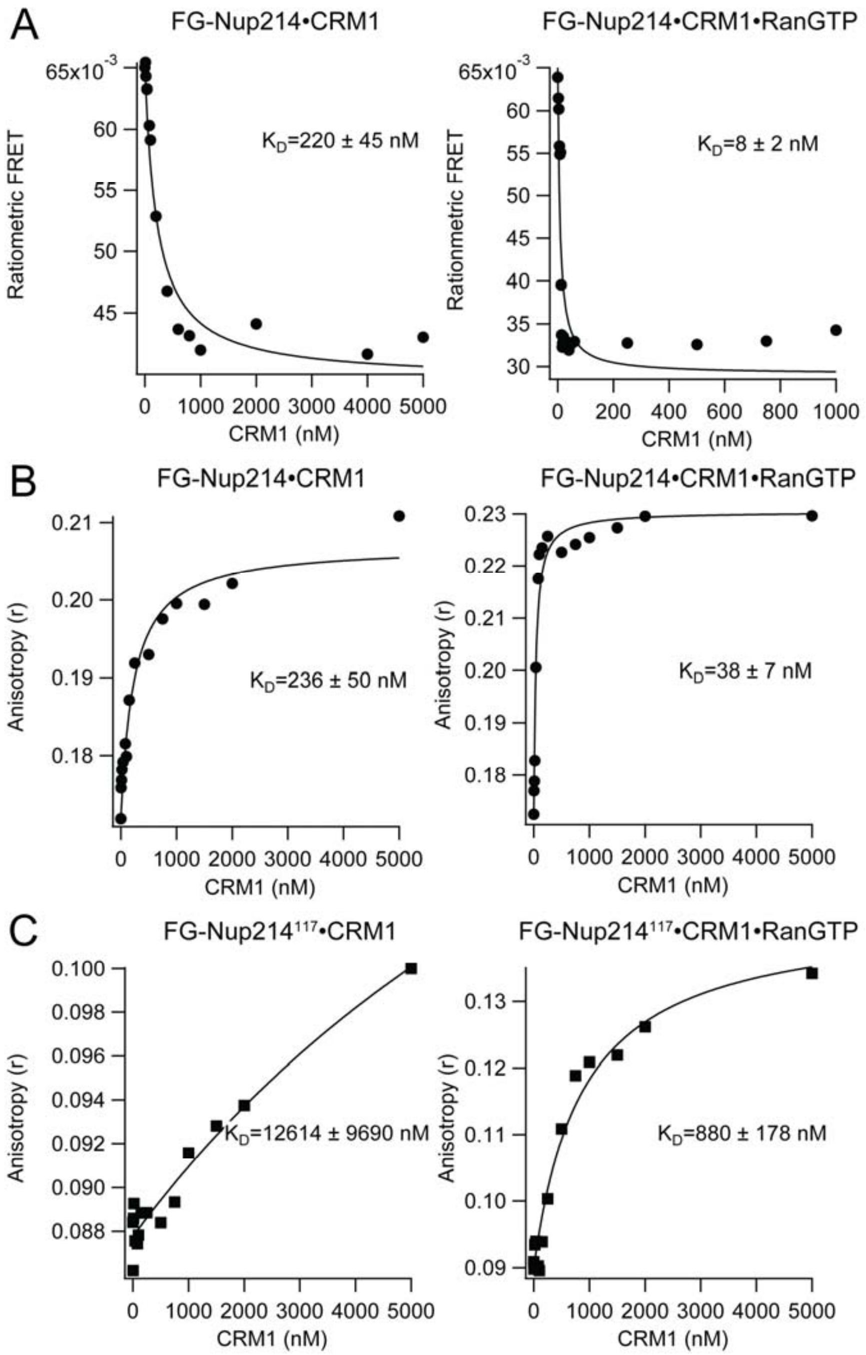

Figure S2. K_D determination of FG-Nup214 with CRM1 and CRM1•RanGTP complex, Related to Figure 1.

(A) FRET titration and (B) anisotropy titration for FG-Nup214. At a concentration of 10 nM FG-Nup214, CRM1 was titrated from 0 nM up to 5000 nM. Both measurements showed that RanGTP (added at a concentration of 1000 nM or same concentration of CRM1, whichever is higher) increases the binding affinity of FG-Nup214 to CRM1. This agrees well with previous findings that CRM1 forms a high affinity, RanGAP-resistant complex with RanGTP and FG-Nup214 (Hutten and Kehlenbach, 2006; Port et al., 2015), (C) Anisotropy titration for FG-Nup214^{117} showed that the affinity of CRM1 on its own for the short fragment is lower so that despite excess of CRM1, the majority of labeled FG-Nup214^{117} can be expected to be unbound in the smFRET experiments.

Figure S3

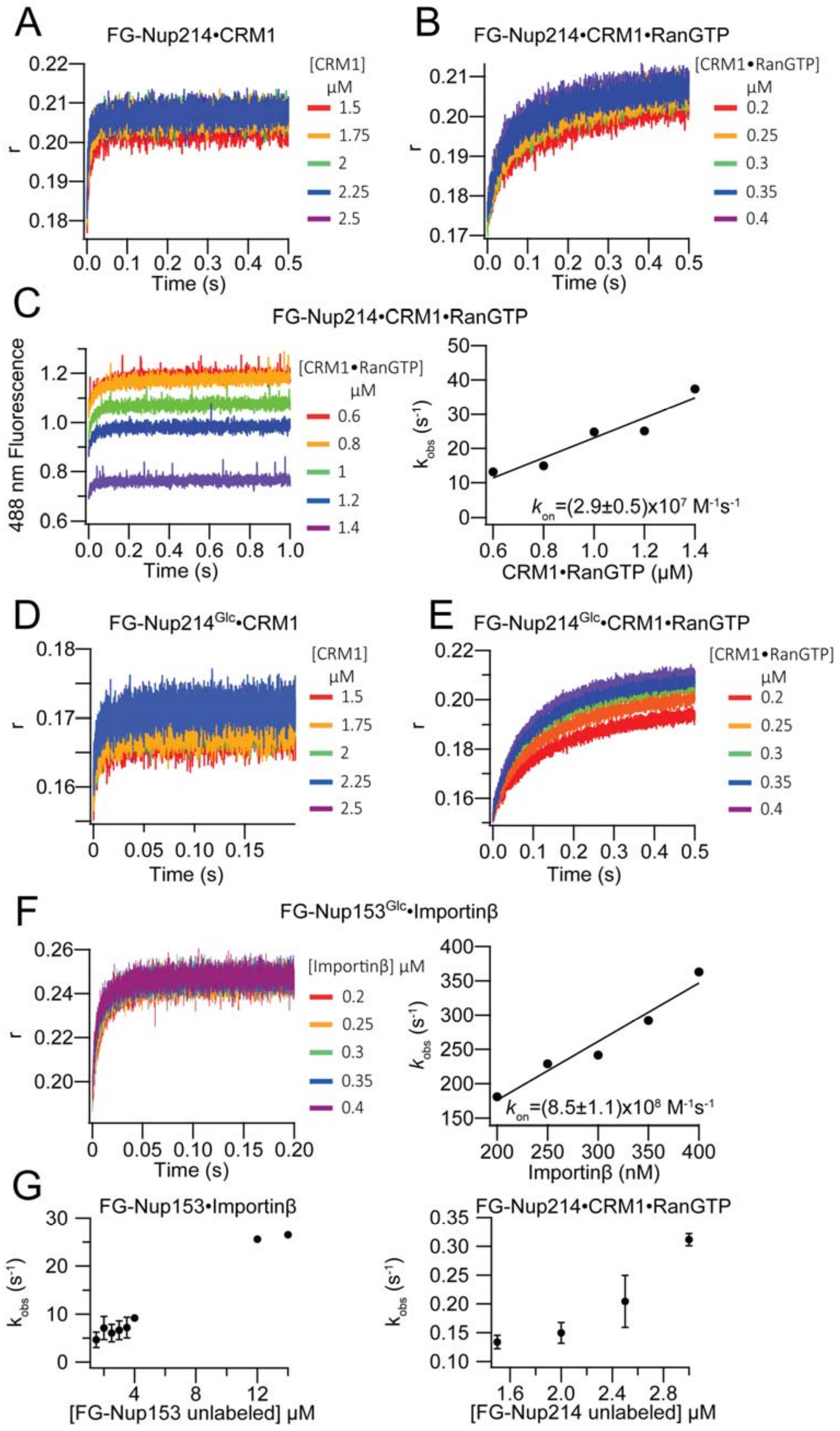
A
FG-Nup214•CRM1
[CRM1] μM
1.5
1.75
2
2.25
2.5
Time (s)
r
B
FG-Nup214•CRM1•RanGTP
[CRM1•RanGTP] μM
0.2
0.25
0.3
0.35
0.4
Time (s)
r
C
FG-Nup214•CRM1•RanGTP
488 nm Fluorescence
[CRM1•RanGTP] μM
0.6
0.8
1
1.2
1.4
Time (s)
k_{obs} (s^{-1})
k_{on}=(2.9±0.5)x10^7 $M^{-1}s^{-1}$
CRM1•RanGTP (μM)
D
FG-Nup214^{Glc}•CRM1
[CRM1] μM
1.5
1.75
2
2.25
2.5
Time (s)
r
E
FG-Nup214^{Glc}•CRM1•RanGTP
[CRM1•RanGTP] μM
0.2
0.25
0.3
0.35
0.4
Time (s)
r
F
FG-Nup153^{Glc}•Importinβ
[Importinβ] μM
0.2
0.25
0.3
0.35
0.4
Time (s)
r
k_{obs} (s^{-1})
k_{on}=(8.5±1.1)x10^8 $M^{-1}s^{-1}$
Importinβ (nM)
G
FG-Nup153•Importinβ
k_{obs} (s^{-1})
[FG-Nup153 unlabeled] μM
FG-Nup214•CRM1•RanGTP
k_{obs} (s^{-1})
[FG-Nup214 unlabeled] μM

Figure S3. Stopped-flow kinetic measurements, Related to Figure 2.

Raw anisotropy traces (r vs time) of 20 nM FG-Nup214 upon mixing with excess of (A) CRM1, (B) CRM1•RanGTP under pseudo-first order conditions. (C) Raw donor fluorescence vs time traces (left) of the double labeled FRET sample upon binding to CRM1•RanGTP, k_{obs} vs [CRM1•RanGTP] plot obtained from fitting the raw traces (right). Raw anisotropy traces (anisotropy vs time) of glycosylated FG-Nup214^{Glc} upon mixing with excess of (D) CRM1, (E) CRM1•RanGTP. (F) Raw anisotropy traces (anisotropy vs time) of FG-Nup153^{Glc} upon mixing with excess of Importinβ. k_{obs} extracted from single exponential fit to these traces are plotted as a function of NTR concentration (right) and the gradient used to obtain the association rate constants (k_{on}). (G) k_{obs} vs [unlabeled FG-Nup] plots, the k_{obs} values were obtained from the stopped-flow dissociation experiments of FG-Nup153•Importinβ (left) and FG-Nup214•CRM1•RanGTP (right) at different concentrations of unlabeled FG-Nup153 and FG-Nup214 respectively. All NTRs were titrated with the range of values as shown in their corresponding colour legend in each experiment. Error bars indicate the standard deviation from the mean of 3 replicates under the same NTR concentration. We note, that for FG-Nup214 for experimental reasons (i.e. aggregation) it was not possible to increase the concentration any higher. For FG-Nup153 at concentrations above 4 μM only single data points are shown (i.e. no error bars), as each point consumed ~24L of expression culture.

Figure S4

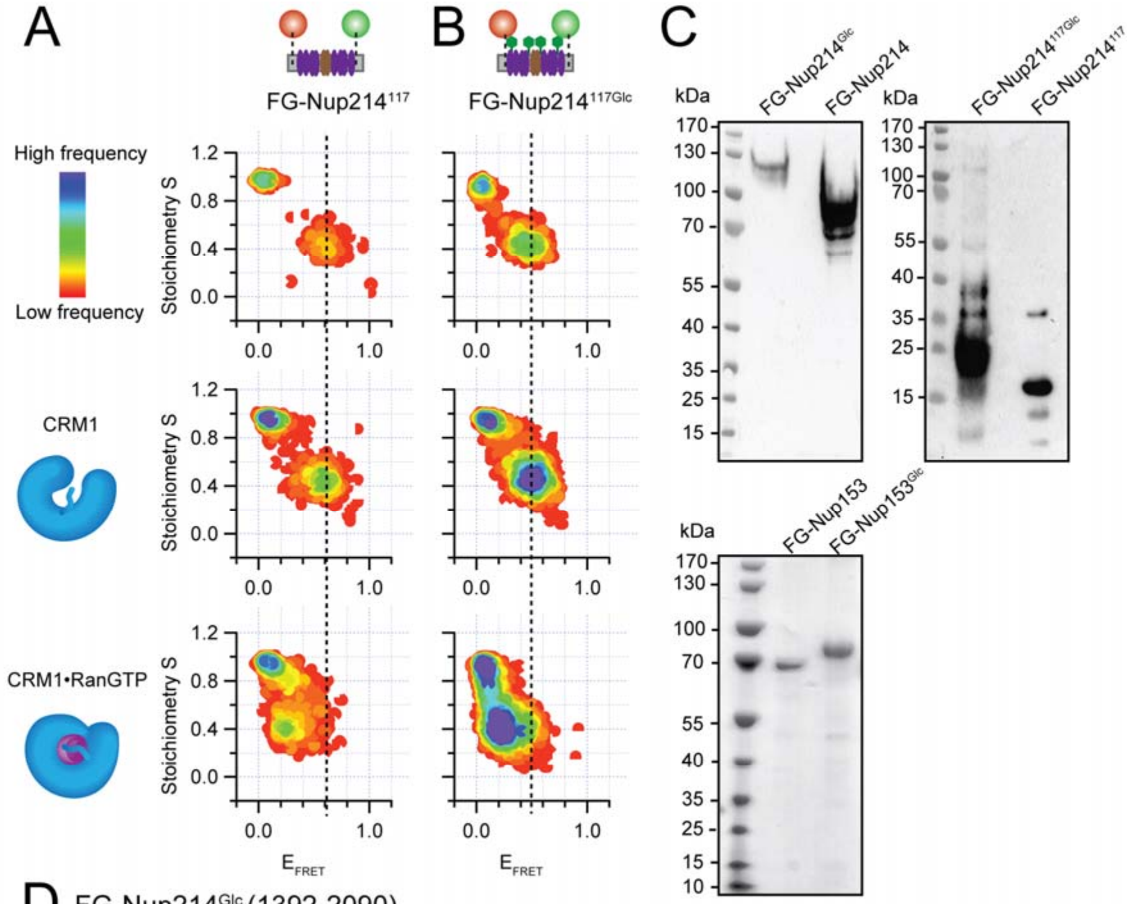

D FG-Nup214^{Glc} (1392-2090)

ATTTSVAPPAATSTSSTAVFGSLPVTSAGSSGVISFGGTSLSAGKTSFSFGSQQTNSTVPPSAPPPTTAATPLPTSFPTLSFGSLLS
SATTPSLPMSAGRSTEEATSSALPEKPGDSEVSASAASLLEEQQSAQLPQAPPQTSDSVKKEPVLAQPAVSNSGTAASSTSLVAL
SAEATPATTGVPDARTEAVPPASSFSVPGQTAVTAAAISSAGPVAVETSSTPIASSTTSIVAPGPSAEAAAFGTVTSGSSVFAQPPA
ASSSSAFNQLTNNTATAPSATPVFGQVAASTAPSLFGQQTGSTASTAAATPQVSSSGFSSPAFGTTAPGVFGQTTFGQASVFGQ
SASSAASVFSFSQPGFSSVPAFGQPASSTPTSTSGSVFGAASSTSSSSSFSFGQSSPNTGGGLFGQSNAPAFGQSPGFGQGG
SVFGGTSAATTTAATSGFSFCQASGFGSSNTGSVFGQAASTGGIVFGQQSSSSSGSVFGSGNTGRGGGFFSGLGGKPSQDAA
NKNPFSSASGGFGSTATSNTSNLFGNSGAKTFGGFASSSFGEQKPTGTFSSGGGSVASQGFGFSSPNKTGGFGAAPVFGSPPT
FGGSPGFGGVPAFGSAPAFTSPLGSTGGKVFGEGTAAASAGGFGFGSSSNTTSFGTLASQNAPTFGSLSQQTSGFGTQSSGF
SGFGSGTGGFSFGSNNSSVQGFGGWRS

E FG-Nup214^{117Glc} (1906-2042)

ASGGFGSTATSNTSNLFGNSGAKTFGGFASSSFGEQKPTGTFSSGGGSVASQGFGFSSPNKTGGFGAAPVFGSP
PTFGGSPGFGGVPAFGSAPAFTSPLGSTGGKVFGEGTAAASAGGFGFGSSSNTTSFGTLASQNA

F FG-Nup153^{Glc} (875-1475)

SAKPGTKSGFKGFDTSSSSSNSAASSSFKFGVSSSSSGPSQTLTSTGNFKFGDQGGFKIGVSSDSGSINPMSEGFKF
SKPIGDFKFGVSSESKPEEVKKDSKNDNFKFGLSSGLSNPVSLTPFQFGVSNLGQEEKKEELPKSSSAGFSFGTGVIN
STPAPANTIVTSENKSSFNLGTIETKSASVAPFTCKTSEAKKEEMPATKGGFSFGNVEPASLPSASVFVLGRTEEKQQE
PVTSTSLVFGKKADNEEPKCQPVFSFGNSEQTKDENSSKSTFSFSMTKPSEKESEQPAKATFAFGAQTSTTADQGAA
KPVFSFLNNSSSSSSTPATSAGGGIFGSSTSSSNPPVATFVFGQSSNPVSSSAFGNTAESSTSQSLLFSQDSKLATTSS
TGTAVTPFVFGPGASSNNTTTSGFGFGATTTSSSAGSSFVFGTGPSAPSASPAFGANQTPTFGQSQGASQPNPPGFG
SISSSTALFPTGSQPAPPTFGTVSSSSQPPVFGQQPSQSAFGSGTTPNSSSAFQFGSSTTNFNFTNNSPSGVFTFGAN
SSTPAASAQPSGSGGFPFNQSPAAFTVGSNGKNVFSSSGTSFSGRKIKTAVRRRK

Figure S4. Conformational change of FG-Nup214^{117} and FG-Nup214^{117Glc} in the presence and absence of CRM1 and CRM1•RanGTP probed by smFRET, Related to Figure 1 and 3.

Analog to Figure 1 and 3, this figure shows the same experiments for the fragment FG-Nup214^{117}. (A) unglycosylated and (B) glycosylated in the absence and presence of 1000 nM CRM1 and CRM1•RanGTP (at 1000 nM for each protein). The dotted line visualizes the shift of the E_{FRET} peak. FG-Nup214^{117Glc} does not show a conformational change upon interacting to CRM1. However, upon addition of CRM1•RanGTP there is a shift in the double labeled population (S= 0.5) showing an E_{FRET} value (= 0.2). A similar E_{FRET} change takes place in the unglycosylated and glycosylated form. This indicates overall conservation of the binding mechanism independent of the glycosylation status. (C) Western blot analysis of FG-Nup214 probed with mAB414 and SDS-PAGE of FG-Nup153. (D-F) The results of a LC-MS/MS analysis underlining the high amount of putative O-glycosylated sites. Protein sequence of (D) FG-Nup214^{Glc}, (E) FG-Nup214^{117Glc} and (F) FG-Nup153^{Glc} showing both FG-Nup214^{Glc} and FG-Nup153^{Glc} were heavily glycosylated and several potential glycosylation sites were identified. Proteins were digested using chymotrypsin in order to receive best coverage of the protein regions of interest. The respective proteins could be identified with a high confidence. The sequences for which peptide evidence were detected are underlined. Further, during the database search using Mascot, O-glycosylation of serine and threonine were selected and putative modified sites are indicated in red color. In order to receive a higher confidence in the predicted sites of modification the Mascot ion score was raised to 32.

Figure S5

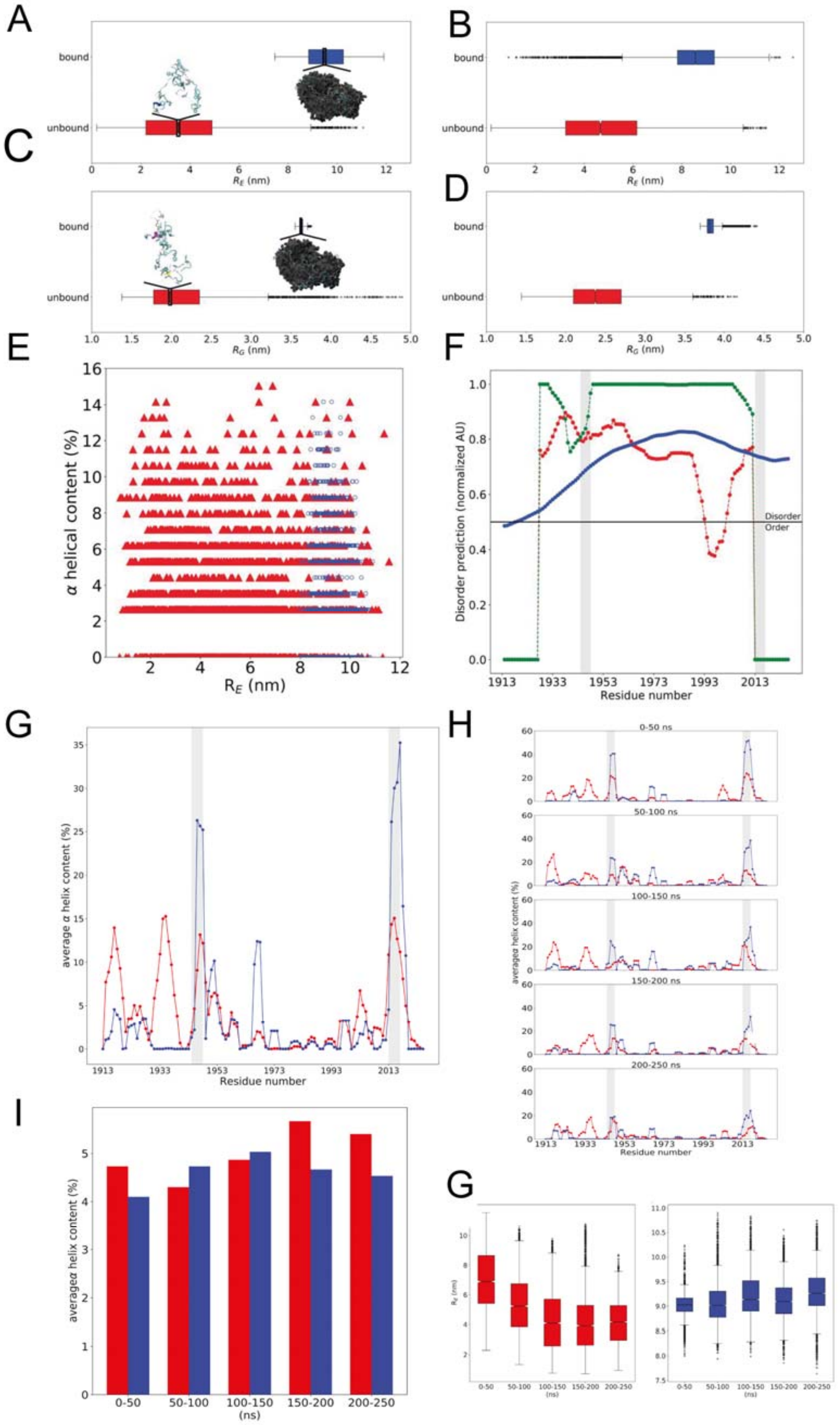
A
B
C
D
E
F
G
H
I
G
bound
unbound
R_E (nm)
R_G (nm)
α helical content (%)
R_E (nm)
Disorder prediction (normalized AU)
Disorder
Order
Residue number
average α helix content (%)
0-50 ns
50-100 ns
100-150 ns
150-200 ns
200-250 ns
averageα helix content (%)
0-50
50-100
100-150
150-200
200-250
(ns)

Figure S5. Analysis of FG-Nup214^{117} dimensions in its bound and unbound state and computationally sampled and predicted secondary structure content and overall dimensions of FG-Nup214^{117}, Related to Figure 4.

Box and whisker plots showing the computed end-to-end (R_E) distance (A-B) and radius of gyration (R_G) (C-D) distributions for FG-Nup214^{117} in the unbound (red) and bound states (blue) sampled with AMBER99-sb*-ILDN-TIP4PD (A and C) and KBFF-SPC/E (B and D) force fields and water models. The distributions were calculated considering the last 150 ns of a total of 250 ns production runs. Each box indicates the range (interquartile range; IQR) between the lower (Q1) and upper (Q3) quartile whereas the solid line represents the distribution's median. The whiskers report values that fall below Q1 – 1.5*IQR or above Q3 + 1.5*IQR. Outliers are shown as crosses below or above whiskers. (E) α-helical content of the FG-Nup214 protein as a function of the R_E for the unbound (red) and bound states (blue). (F) Sequence-based prediction of disorder-propensity for the simulated FG-Nup214 peptide using the XL1-XT (red (Romero, 1997)), VL3-BA (blue (Kehlenbach et al., 1999)) and CaN-XT (green (Garner, 1999)) algorithms available in PONDR (http://www.pondr.com/). The arbitrary threshold distinguishing ordered and disordered sequences is reported at 0.5 intrinsic disorder prediction score and shown as a horizontal black line. (G) Secondary structure analysis of the amount of α-helical conformation for each residue as found along the simulated trajectories of FG-Nup214^{117} in isolation (red) and in complex to CRM1•RanGTP (blue). (H) Percentage of α-helical content along the FG-Nup214^{117} sequence is reported for five different 50 ns-long time windows for the unbound (red) and bound (blue) state, collected for each of the 10 replicates. (I) Average α-helical content, shown in percentage per time window, for the FG-Nup214^{117} bound and unbound state. (J) R_E distributions as a function of five different 50 ns-long time windows collected for each of the 10 replicates for the unbound (red) and bound (blue) state of FG-Nup214^{117}.

Figure S6

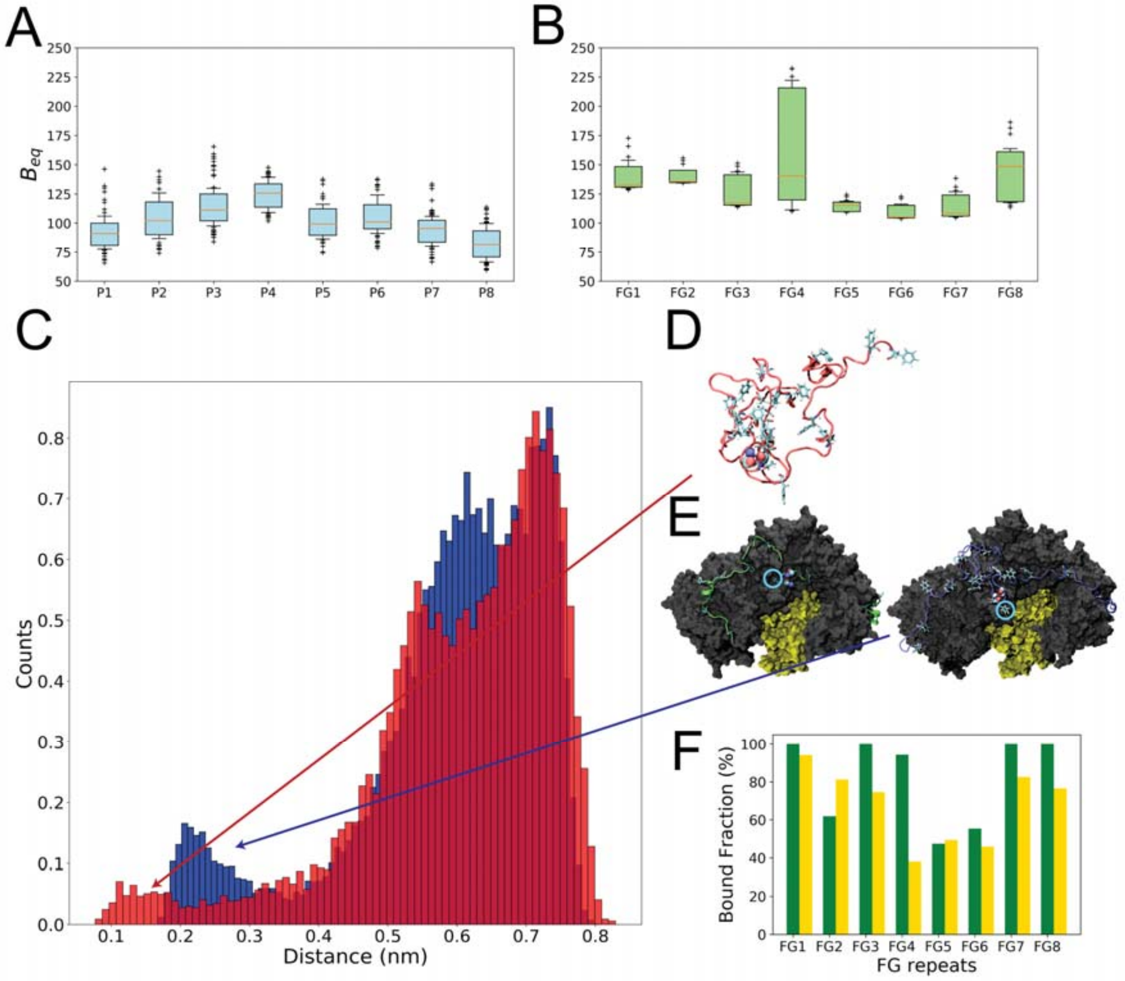

Figure S6. Equal B-factor (B_{eq}) of CRM1 FG-binding pockets/ co-crystallized FG-Nup214[117] FG repeats and distance distributions of the T1981-G1984 backbone hydrogen bond and fraction of FG-Nup214[117] FG repeats bound to CRM1, Related to Figure 4.

(A) B_{eq} distributions for the residues composing the CRM1 binding pockets allocating the eight FG repeats (FG1-FG8) as observed by (Port et al., 2015) and for which the B_{eq} distributions are reported in (B). Each box indicates the range (interquartile range; IQR) between the lower (Q1) and upper (Q3) quartile whereas the solid line represents the distribution's median. The whiskers report values that fall below Q1 – 1.5*IQR or above Q3 + 1.5*IQR. Outliers are shown as crosses below or above whiskers. Distributions have been computed considering the B_{eq} obtained for each atom of the residues, which are described as a part of the analysed pocket or FG repeats. The red lines show the median of each distribution. (C) Distribution of distances between the T1981@O---G1984@H hydrogen bond suggested to be functional to the binding of the FG5 (F1982-G1983) to CRM1. The distribution of distances sampled during MD simulations is reported for both the unbound (red) and bound states (blue) of FG-Nup214[117]. We note that the experimental B-factors for FG repeats follow neither the trend from simulations nor the trend of the B-factors of CRM1 pocket residues, which might be attributed to the challenge in the X-ray refinement of broad conformational ensembles (Kuzmanic et al., 2014). (D) A representative conformation recording the lowest distance for the analysed bond; in the majority of cases such a low distance is found in globule-like conformations, which are far from the bound-prone semi-extended pose observed in the complex. (E) Conformations of the FG-Nup214[117]•CRM1•RanGTP complex co-crystallized by Port *et al.* (left panel) and corresponding (right) to the lower end of the h-bond distance distribution sampled in the bound state (blue) (Port et al., 2015). F1982 is highlighted by a cyan circle. (F) The fraction of FG-Nup214[117] FG repeats bound to CRM1 is shown as observed from the simulations performed using the

AMBER99-sb*-ILDN (green bars) or the KB (yellow bars) force fields. The bound fraction of each FG repeat was calculated considering the number of contacts between a repeat and any atom of CRM1 within a radius of 0.4 nm. Frames in which the number of contacts were different from 0 were considered as ‘bound’, whereas frames in which no contacts were observed were considered as ‘unbound’. The percentage of bound fraction has then been retrieved by normalising the fraction of frames reporting binding over the total number of simulated frames.

Figure S7

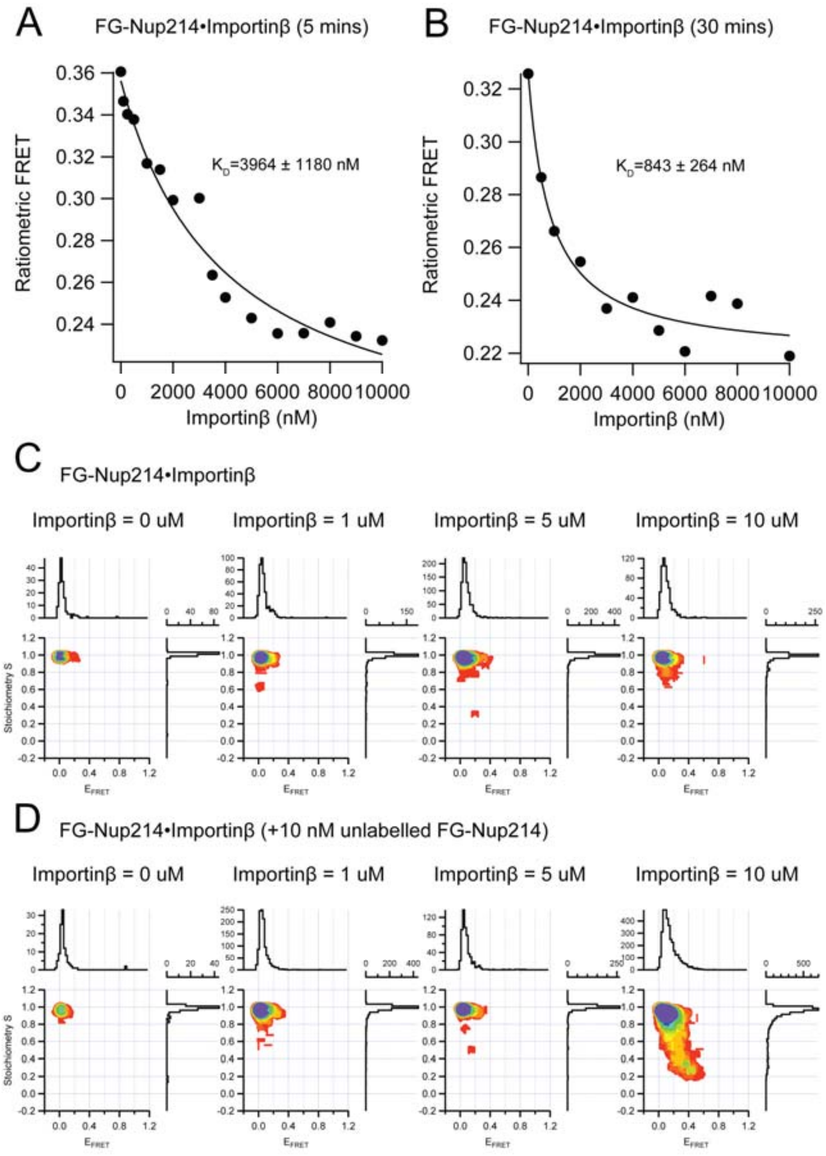

Figure S7. Time and concentration dependence of FG-Nup214•Importinβ, Related to Figure 5.

Ensemble FRET measurements to study incubation time dependent K_D of FG-Nup214•Importinβ interactions. We observed different K_D value for different titrations of Importinβ (ranging from 0 to 10000 nM) interacting with 10 nM FG-Nup214 performed after different incubations times of (A) 5 minutes and (B) 30 minutes. Next, we separately labeled FG-Nup214 with Alexa488 and Alexa594 and created 1:1 mixture at a total protein concentration of 50 pM. SmFRET measurements to detect coincide of the two dyes (i.e. population of S>0) were performed with 10 nM unlabeled FG-Nup214 (D) and without (C). Under both conditions Importinβ was titrated in ranging from 0 to 10 µM. Plots show clearly an appearance of a population with FRET and S higher than 0 in the present of high concentration of Importinβ ~(10 µM) when interacting with nM concentration of FG-Nup214. This clearly indicates aggregation, in which donor and acceptor dye (tentatively from different molecules) come even closer than 10 nm together and can undergo FRET. We learn from this that under smFRET conditions used in Figure 1 and 5, FRET is not originating from aggregation. However, FG-Nup214 and Importinβ has also a more complex phase diagram, so that at other time points and or higher concentrations, also aggregation phenomena can occur and lead to FRET, in line with previous observations for other Nup and NTR combinations (Kehlenbach et al., 1999; Milles et al., 2013).

Supplemental Experimental Procedures

Protein Expression, Purification and Labeling

FG-Nup214/ FG-Nup214^{117}: The codon-optimized (Mr. Gene, Regensburg, Germany) FG-Nup214 disordered region (amino acids 1392 to 2090 and 1906 to 2042 for FG-Nup214^{117}, numbering with respect to the full length protein Uniprot: P35658 protein) was recombinantly expressed in *Escherichia coli* (*E.coli*) strain BL21(AI) (Invitrogen, Carlsbad,CA). For 117 aa long FG-Nup214 fragment, the final purified sequence was 137 aa (1906 to 2042) to incorporate the FRET dye pair. Plasmids from the labeling mutants were cotransformed with pEvol-AcF, which encodes for the noncanonical amino acid p-acetylphenylalanine (AcF) Amber suppressor tRNA/tRNAsynthetase pair (Lemke, 2011). Cultures were grown in Terrific Broth (TB) medium at 37ºC in the presence of 1 mM AcF. Expression was induced at OD_{600}= 1 with 0.02% arabinose and 1 mM IPTG. The detailed protocol for FG-Nup214 purification was as previously described for FG-Nup153 in (Milles and Lemke, 2011).

CRM1: CRM1 was recombinantely expressed in *E.coli* BL21 (AI) from a pQE60-CRM1-intein-12His vector. Cultures were grown in TB medium at 37ºC and induced with 1 mM IPTG. After induction, temperature was reduced to 18ºC and incubated for 18 hours. *E.coli* cells were harvested and purified under native conditions according to standard purification protocols for His-tagged proteins.

RanQ69L (1-180): RanQ69L protein was expressed and purified in the same way as CRM1. His-tag was cleaved with by a TEV protease. GTP loading of RanQ69L was performed by incubating the purified protein with the non-hydrolyzable GTP (γ-S-GTP) nucleotide in a ratio 1:20 (protein: nucleotide) in presence of 10 mM EDTA, 1 µl of alkaline phosphatise per 10 nmoles protein. The reaction was incubated at 20^{0}C for 2 hours. The sample was centrifuged (13,000 rpm, 15 minutes) to remove aggregates. Then the supernatant was supplemented with $MgCl_2$ to a final concentration of 15 mM to stabilize the nucleotide bound to RanQ69L protein.

OGT: O-Linked N-Acetylglucosamine (GlcNAc) Transferase tagged with a 12His was cloned into a pTXB3 vector and expressed in BL21 (AI cells). Cells were induced at OD_{600}: 0.6-0.8 and OGT were expressed at 18ºC overnight under shacking conditions. Cells were pelleted, resuspended and lysed on lysis buffer (50 mM Tris, pH 7.4, 150 mM NaCl, 5 mM imidazole and the buffer was supplemented with 0.2 mM TCEP and 1 mM PMSF) After centrifugation for 1 h at 18.000 rpm the supernatant was incubated with Ni-beads. After extensive washes with 10-20 mM imidazole the protein was eluted with 400 mM imidazole. Eluted OGT1 was then concentrated and run over size exclusion chromatography (adapted protocol from (Labokha et al., 2013)). The selected fractions where then concentrated and buffer exchanged with 50 mMTris, pH 7.4, 150 mM NaCl, 0.2 mM TCEP ,1 mM PMSF and 20 % glycerol. The samples where flash frozen and kept at -80ºC.

Labeling of FG-Nup214/ FG-Nup214^{117} were performed by using standard protocol to site-specifically introduce Alexa488 as a donor and Alexa594 as an acceptor as described in (Milles and Lemke, 2011). Single cysteines and Amber stop codons (TAG) were introduced into the FG-Nup214 and sequence by site-directed mutagenesis. The purified labeling mutants were site-specifically labeled with Alexa594-maleimide as acceptor and the AcF was labeled with Alexa488-hydroxylamine. The labeling positions of FG-Nup214 were determined based on the architecture of the CRM1•SPN1•RanGTP•FG-Nup214 complex (PDB: 5DIS). Labeling of FG-Nup214 for stopped-flow measurements was performed with Cy3B-maleimide.

In vitro glycosylation of FG-Nup

FG-Nup214 and FG-Nup153 were glycosylated with UDP-GlcNAc (Sigma: U4375), which were attached to serine and threonine residues and the process was catalysed by the O-GlcNAc transferase (OGT1). The *in vitro* glycosylation was performed following the protocol described by (Labokha et al., 2013). FG-Nup from cell lysate where washed with washing buffer (as described in the Nucleoporin purification section mentioned above), then washed with glycosylation buffer (50 mM Tris, pH 7.5, 200 mM NaCl, 20 mM $MgCl_2$, 0.2 mM TCEP and 1 % Tween20). The beads where then resuspended in 20 bead volumes of glycosylation buffer containing 5 µM OGT and 1 mM UDP-GlcNAc. The beads where rotated at room temperature for 16 h. The beads where washed with glycosylation buffer and subsequently with the washing buffer used in the FG-Nup purification protocol.

Molecular modelling and molecular dynamics simulations of the FG-Nup214^{117} and FG-Nup214^{117}•CRM1•RanGTP complexes

Simulations were started after modelling parts of the bound partners that couldn't be resolved by X-ray crystallography. The lack of any known conformation of FG-Nup214^{117} made the modelling of the protein starting from homology to a template impossible. Therefore, the missing segments of FG-Nup214 were modelled in a random conformation subsequently equilibrated during the first 150 ns of the molecular dynamics run. The nucleoporin were modelled by adding each residues in the most populated rotameric population according to the Dunbrack library (Dunbrack, 2002), making sure that any atom of the added conformers would not clash with any resolved atoms of the X-ray crystal structure. The complexes featuring the modelled Nup214^{117} were then prepared for simulations as described below. Firstly, hydrogen atoms were added and molecular topologies were built adopting the parameters defined by the AMBER99-sb*-ILDN force field (Lindorff-Larsen et al., 2010). Molecules were then placed into a dodecahedron box filled with TIP4PD water molecules (Piana et al., 2015). The TIP4PD water model has been explicitly parameterized to avoid the artificial over-collapse of intrinsically disordered proteins but, at the same time, it has shown to avoid an excessive destabilization of structured proteins such as, in this case, CRM1 and RanGTP. Additionally, we performed simulations also employing the Kirkwood-Buff force field (Ploetz et al., 2010) and compared the results obtained for the FG-Nup214^{117} ensembles. Importantly, the combination of AMBER99-sb*-ILDN with TIP4PD has been shown to yield realistic dimensions of IDPs and to destabilise structured proteins by only ~8.4 kJ mol^{-1} (Piana et al., 2015). Na^+ and Cl^- Ions were added to the solutions by replacing water molecules in the box to finally achieve systems' neutrality and a total ionic concentration of 0.1 M. The systems were then energy minimised using a steepest descent algorithm with a step size of 0.001 nm, stopped when the force was lower than 1 kJ mol^{-1}. Subsequently, solvent equilibration around the molecules was achieved in two steps. Firstly, a 0.5 ns-long run was performed at a constant number of particles, volume and temperature (NVT). In this step, temperature was coupled to a 300 K using a V-rescale thermostat (Bussi et al., 2007) and starting velocities were randomly generated for each particle following a Brownian distribution at 300 K. Secondly, a 0.5 ns-long NpT step, was carried out by coupling both pressure and temperature at 1 atm and 300 K respectively, using a Parrinello-Rahman barostat (Parrinello and Rahman, 1981) and a V-rescale thermostat. Pressure and temperature were coupled every 2.0 and 0.1 ps respectively. In both NVT and NpT steps, proteins were restrained in the three dimensional space by applying on each particle a potential of 1000 kJ mol^{-1}. Subsequently to equilibration, production runs were performed on 10 different replicates, differing for the initial random velocities assigned in the NVT equilibration step and each step was run for 125 million steps using a time step of 2 fs, finally yielding a production run time of 250 ns for each replicate and the first 150 ns were discarded for analysis as considered equilibration time. Interactions between the particles were computed using the Verlet scheme (Páll and Hess, 2013) for neighbouring search and a cutoff for Lennard-Jones and Coulomb interactions of 1 nm. The analysis of the trajectories was achieved using tools available within the GROMACS suite (Van Der Spoel et al., 2005) and routines implemented in the analysis suite called MDAnalysis (Michaud-Agrawal et al., 2011). Visualization and rendering of the structures was performed using visual molecular dynamics (VMD) version 1.9.3 (Humphrey et al., 1996). Data plotting and visualisation was finally performed using matplotlib (Hunter, 2007). Disorder predictions were performed using PONDR web server (http://pondr.com) and employing three main algorithms available in it: VL3-BA (Milles et al., 2013), CAN_XT (Garner, 1999) and XL1-XT (Romero, 1997).

SmFRET experiment and data analysis

The smFRET experiments were performed using a confocal-based microscope with a 1.27 numerical aperture (NA) and a 60x water immersion objective (Nikon). The fluorescence emission signal was split into donor and acceptor channels, with both parallel and perpendicular polarization orientation. The fluorescence signal was then detected on photon counting detectors (APD and hybrid PMA, Picoquant, Berlin) and directed to counting electronics (HydraHarp400, Picoquant, Berlin). The fluorescence intensities were used to calculate the FRET efficiencies (E_{FRET}) by the corrected FRET equation: $E_{FRET} = I_A/(I_A + \gamma I_D)$, where γ is the correction factor which accounts for the differences in quantum yield and detection efficiency between the donor and the acceptor, I_A and I_D are the acceptor and donor intensities, respectively. The data analysis was performed by a custom written Igor-Program (Fuertes et al., 2017).

The 2D S vs E_{FRET} plots obtained from pulse interleaved excitation (PIE) (Muller et al., 2005) show the populations according to the stoichiometry of the dyes (y axis) on the disordered protein being S= 1 for the molecules labeled with donor only dye and S= 0.5 for the molecules containing a donor and an acceptor dye

with 1:1 ratio and S= 0 for the molecule labeled with acceptor dye only. Donor or acceptor only population can arise from dye photophysics and/or incomplete labeling. The population at S=0.5 is thus the one in which we monitored possible changes in the E_{FRET} values (x axis). E_{FRET} value shifting towards 0 indicates an increase of the distance between the dyes, leading to a decrease of the efficiency of energy transferred from the donor to the acceptor dye. All the smFRET experiments in this paper were performed at 50 pM FG-Nup and 1 μM of excess NTR. Concentration ranges are given in the respective data sets. Excess of the NTR does not necessarily indicate that binding could be saturated. In particular in smFRET, due to the stringent signal to noise requirement, to high NTR concentration can lead to large background that would compromise signal quality too much.

Fluorescence stopped-flow measurement

Association kinetics were monitored by following the fluorescence anisotropy change of 20 nM FG-Nup214 labeled with Cy3B (at the position 1905 in relation to the full length) upon mixing with different concentrations, ranging from 0.2-2.4 μM, of CRM1 and RanGTP under pseudo-first order conditions. Due to the higher affinity, kinetic experiments were performed only for full length FG-Nup214 domain in all experiments. Traces were fit to a single exponential function and the concentration dependent observed rates (k_{obs}) were then plotted against their corresponding NTR concentration. From the slope of the linear fit the association rate constant (k_{on}) was obtained. Analogous to FG-Nup214, 20 nM of FG-Nup153 labeled at position 1391C with Cy3B was used for the association and dissociation measurements of FG-Nup153 and Importinβ. The dissociation experiments were performed with 20 nM FG-Nup and 0.5 μM CRM1/RanGTP or 1 μM Importinβ. The preformed complexes were then rapidly mixed with 1.5 to 14 μM unlabeled FG-Nup (see below). The estimated dissociation rate constants were obtained by fitting an exponential decay to the observed anisotropy traces.

Dissociation rate constant (k_{off}) determination in multivalent systems

The kinetic dissociation measurements show a k_{off} at least one order of magnitude difference between FG-Nup153•Importinβ and FG-Nup214•CRM1•RanGTP complexes. While we do acknowledge that some differences can also occur from different effects of multivalency and the intrinsic property of segmental motion of IDPs in anisotropy measurements (Milles and Lemke, 2014), FG-Nup153 has 60 Fs and FG-Nup214 has 62 Fs and the recorded complex half-life is at least 60 fold higher for the CRM1•RanGTP case (Figure 2B). Thus another major biophysical parameter (k_{off}) is different for the two FG-Nup•NTR complexes.

Our dissociation kinetic experiments of multivalent proteins like FG-Nup153 and FG-Nup214 report on the $k_{off,global}$ value which corresponds to when ~all of the binding motifs of the disordered nucleoporin are unbound from the multivalent NTR, and not on $k_{off,\ individual}$. The relationship between $k_{off,\ individual}$ and $k_{off,global}$ depends on multivalency, linker length and effective concentration. In a multivalent system such as ours, the $k_{off,global}$ value obtained from a kinetic dissociation experiment will thus be orders of magnitude lower than those for each motif ($k_{off,\ individual}$) (Kramer and Karpen, 1998). Taking multivalency into account is of paramount importance if one aims to understand NTR transport and interactions with FG-Nups.

In addition, due to the reversible binding of FG-Nup•NTR complexes, we performed the dissociation kinetic experiments under different concentrations of unlabeled FG-Nup (Figure S3G). The obtained k_{obs} increased with increasing concentrations of unlabeled FG-Nup. Due to experimental constraints we were not able to reach the conditions at which the k_{off} of both systems reached a plateau since at very high concentrations of unlabeled protein, these long disordered proteins tend to aggregate. For this reason it is possible that the differences in the complex half-life are larger than the reported 60 fold obtained at 2 μM (100x excess) of unlabeled FG-Nup.

Supplemental references

Bussi, G., Donadio, D., and Parrinello, M. (2007). Canonical sampling through velocity rescaling. J Chem Phys *126*, 014101.

Dunbrack, R.L. (2002). Rotamer libraries in the 21(st) century. Curr Opin Struc Biol *12*, 431-440.

Fuertes, G., Banterlea, N., Ruff, K.M., Chowdhury, A., Mercadante, D., Koehler, C., Kachala, M., Girona, G.E., Milles, S., Mishra, A., *et al.* (2017). Decoupling of size and shape fluctuations in heteropolymeric sequences reconciles discrepancies in SAXS vs. FRET measurements. Proceedings of the National Academy of Sciences of the United States of America *114*, E6342-E6351.

Garner, E., Romero, P., Dunker, A.K., Brown, C. & Obradovic, Z (1999). Predicting Binding Regions within Disordered Proteins. Genome Inform Ser Workshop Genome Inform *10*, 41-50.

Humphrey, W., Dalke, A., and Schulten, K. (1996). VMD: visual molecular dynamics. J Mol Graph *14*, 33-38, 27-38.

Hunter, J.D. (2007). Matplotlib: A 2D graphics environment. Computing In Science & Engineering *9*, 90-95.

Hutten, S., and Kehlenbach, R.H. (2006). Nup214 is required for CRM1-dependent nuclear protein export in vivo. Molecular and Cellular Biology *26*, 6772-6785.

Kalinin, S., Valeri, A., Antonik, M., Felekyan, S., and Seidel, C.A.M. (2010). Detection of Structural Dynamics by FRET: A Photon Distribution and Fluorescence Lifetime Analysis of Systems with Multiple States. Journal of Physical Chemistry B *114*, 7983-7995.

Kehlenbach, R.H., Dickmanns, A., Kehlenbach, A., Guan, T.L., and Gerace, L. (1999). A role for RanBP1 in the release of CRM1 from the nuclear pore complex in a terminal step of nuclear export. Journal of Cell Biology *145*, 645-657.

Kramer, R.H., and Karpen, J.W. (1998). Spanning binding sites on allosteric proteins with polymer-linked ligand dimers. Nature *395*, 710-713.

Kuzmanic, A., Pannu, N.S., and Zagrovic, B. (2014). X-ray refinement significantly underestimates the level of microscopic heterogeneity in biomolecular crystals. Nature Communications *5*, 3220.

Labokha, A.A., Gradmann, S., Frey, S., Hulsmann, B.B., Urlaub, H., Baldus, M., and Gorlich, D. (2013). Systematic analysis of barrier-forming FG hydrogels from Xenopus nuclear pore complexes. Embo Journal *32*, 204-218.

Lemke, E.A. (2011). Site-specific labeling of proteins for single-molecule FRET measurements using genetically encoded ketone functionalities. Methods in molecular biology *751*, 3-15.

Lindorff-Larsen, K., Piana, S., Palmo, K., Maragakis, P., Klepeis, J.L., Dror, R.O., and Shaw, D.E. (2010). Improved side-chain torsion potentials for the Amber ff99SB protein force field. Proteins *78*, 1950-1958.

Michaud-Agrawal, N., Denning, E.J., Woolf, T.B., and Beckstein, O. (2011). MDAnalysis: a toolkit for the analysis of molecular dynamics simulations. J Comput Chem *32*, 2319-2327.

Milles, S., Huy Bui, K., Koehler, C., Eltsov, M., Beck, M., and Lemke, E.A. (2013). Facilitated aggregation of FG nucleoporins under molecular crowding conditions. EMBO reports *14*, 178-183.

Milles, S., and Lemke, E.A. (2011). Single Molecule Study of the Intrinsically Disordered FG-Repeat Nucleoporin 153. Biophysical Journal *101*, 1710-1719.

Milles, S., and Lemke, E.A. (2014). Mapping multivalency and differential affinities within large intrinsically disordered protein complexes with segmental motion analysis. Angew Chem Int Ed Engl *53*, 7364-7367.

Muller, B.K., Zaychikov, E., Brauchle, C., and Lamb, D.C. (2005). Pulsed interleaved excitation. Biophysical Journal *89*, 3508-3522.

Páll, S., and Hess, B. (2013). A flexible algorithm for calculating pair interactions on SIMD architectures. Computer Physics Communications *184*, 2641-2650.

Parrinello, M., and Rahman, A. (1981). Polymorphic transitions in single crystals: A new molecular dynamics method. J Appl Phys *52*, 7182-7190.

Piana, S., Donchev, A.G., Robustelli, P., and Shaw, D.E. (2015). Water dispersion interactions strongly influence simulated structural properties of disordered protein States. The journal of physical chemistry B *119*, 5113-5123.

Ploetz, E.A., Bentenitis, N., and Smith, P.E. (2010). Developing Force Fields from the Microscopic Structure of Solutions. Fluid phase equilibria *290*, 43.

Port, S.A., Monecke, T., Dickmanns, A., Spillner, C., Hofele, R., Urlaub, H., Ficner, R., and Kehlenbach, R.H. (2015). Structural and Functional Characterization of CRM1-Nup214 Interactions Reveals Multiple FG-Binding Sites Involved in Nuclear Export. Cell Rep *13*, 690-702.

Romero, O.D., K (1997). Sequence Data Analysis for Long Disordered Regions Prediction in the Calcineurin Family. Genome Inform Ser Workshop Genome Inform *8*, 110-124.

Van Der Spoel, D., Lindahl, E., Hess, B., Groenhof, G., Mark, A.E., and Berendsen, H.J. (2005). GROMACS: fast, flexible, and free. J Comput Chem *26*, 1701-1718.
